# Soundscape in Times of Change: Case Study of a City Neighbourhood During the COVID-19 Lockdown

**DOI:** 10.3389/fpsyg.2021.570741

**Published:** 2021-03-24

**Authors:** Sara Lenzi, Juan Sádaba, PerMagnus Lindborg

**Affiliations:** ^1^DensityDesign Lab, Department of Design, Politecnico di Milano, Milan, Italy; ^2^Department of Architecture, Universidad del País Vasco, San Sebastián, Spain; ^3^School of Creative Media, City University of Hong Kong, Kowloon, Hong Kong

**Keywords:** soundscape, urbanism, perception and cognition, COVID-19, pandemic, social response, case study

## Abstract

The coronavirus disease 2019 (COVID-19) lockdown meant a greatly reduced social and economic activity. Sound is of major importance to people’s perception of the environment, and some remarked that the soundscape was changing for the better. But are these anecdotal reports based in truth? Has traffic noise from cars and airplanes really gone down, so that more birdsong can be heard? Have socially distanced people quietened down? This article presents a case study of the human perception of environmental sounds in an urban neighborhood in the Basque Country between 15 March and 25 May 2020. The social restrictions imposed through national legislation divided the 69-day period into three phases. We collected observations, field audio recordings, photography, and diary notes on 50 days. Experts in soundscape and architecture were presented with the recordings, in randomized order, and made two separate perceptual analyses. One group (*N* = 11) rated the recordings for pleasantness and eventfulness using an adapted version of the Swedish Soundscape Quality Protocol, and a partly overlapping group (*N* = 12) annotated perceived sound events with free-form semantic labels. The labels were systematically classified into a four-level Taxonomy of Sound Sources, allowing an estimation of the relative amounts of Natural, Human, and Technological sounds. Loudness and three descriptors developed for bioacoustics were extracted computationally. Analysis showed that Eventfulness, Acoustic Complexity, and Acoustic Richness increased significantly over the time period, while the amount of Technological sounds decreased. These observations were interpreted as reflecting changes in people’s outdoor activities and behavior over the whole 69-day period, evidenced in an increased presence of Human sounds of voices and walking, and a significant shift from motorized vehicles toward personal mobility devices, again evidenced by perceived sounds. Quantitative results provided a backdrop against which qualitative analyses of diary notes and observations were interpreted in relation to the restrictions and the architectural specifics of the site. An integrated analysis of all sources pointed at the temporary suspension of human outdoor activity as the main reason for such a change. In the third phase, the progressive return of street life and the usage of personal mobility vehicles seemed to be responsible for a clear increase in Eventfulness and Loudness even in the context of an overall decrease of Technological sounds. Indoor human activity shared through open windows and an increased presence of birdsong emerge as a novel characteristic element of the local urban soundscape. We discuss how such changes in the acoustic environment of the site, in acoustic measurements and as perceived by humans, point toward the soundscape being a crucial component of a comprehensive urban design strategy that aims to improve health and quality of life for increasingly large and dense populations in the future.

## Introduction

“Media that emphasize space are apt to be less durable and light in character… such as sounds, for the true character of sound in shaping societies is in its spatial spread… and the real paradox is that although sounds are pronounced in time, they are also erased by time” (Schafer, 1977 p. 162). How does the soundscape change over time? In *The Tuning of the World*, Schafer (1977) describes measuring a collection of fire engine sirens covering seven decades. He found that their signal had gotten louder by “nearly half a decibel per year on average” (idem p. 186). This observation supported his general thesis that urban noise levels have increased in industrialized societies, to the detriment of animals and human inhabitants alike. In what may seem as a reply, Arana (2010) analyzed a large number (876,480) of noise measurements taken in the Spanish cities of Pamplona and Madrid between 1999 and 2003. Contrary to Schafer’s results, he found a statistically significant decrease in the overall sound level. The findings were translated to inspire politicians and designers; for example, the “remarkable reduction” of noise that had been observed in one district was attributed to the implementation of pedestrian areas.

Such investigations are part of a larger movement. The approach to sound and listening that Schafer pioneered in the mid-1960s has broadened out, in particular through the World Soundscape Project and the World Forum for Acoustic Ecology (see Truax, 2019 for a history), and has become a multifaceted field that is deeply connected with urban planning, policy, health, architecture, activism, and art. Interdisciplinarity has suffused research in the past decade, such as the Soundscape Support to Health program (Berglund and Nilsson, 2007) and the Positive Soundscapes Project (Davies et al., 2013). Viewed from this perspective, soundscape studies have a natural affinity with environmental psychology, even if goals and methodologies are sometimes different.

From the perspective of urban and city planning, as the concern for a more active awareness on the perception of environmental sound grows in the 1960s, perhaps influenced by soundscape studies, urban designers propose a more subjective and qualitative approach to the city. In the United States, Jacobs (1961) advocates in *The Death and Life of Great American Cities* (1961) that while looking at real cities “you might as well also listen, linger and think about what you see.” Not far in time, Gehl in Europe claims for a closer attention to the *Life Between Buildings* (1971). The perceivable, intangible aspects of the city environment are linked with physical, tangible components of the architecture as well as the urban design of our human ecosystem. We consider the soundscape as one of the intangible layers of the city. Ultimately, the way we arrange the invisible linkages will determine crucially the form of the city. It will revert to us as a society and will shape behaviors and habits. Thereby, the urban ecosystem we design today is intended for future generations. As shown by Arana (cit.), soundscape research can contribute to improving people’s quality of life through urban planning initiatives.

There are few examples of experimental soundscape studies of the kind found in, e.g., medicine or psychology (but see Aletta et al., 2016b, for a covert intervention study). It would be impractical or unethical, or both, to try to implement a double-blind study on the physical scale of cities or nations, or on the temporal scales of decades that Schafer imagined. That being said, we find ourselves today in an extreme situation, with the coronavirus disease 2019 (COVID-19) pandemic sweeping through human societies in every country and every city. It offers an unusual opportunity to put the acoustic environment to a test, almost as if the pandemic lockdown restrictions in various places were conditions in a social experiment at huge geographical and temporal scales. From the urban design point of view, the situation triggered by the COVID-19 outbreak has opened up possibilities to observe how the city environment changes under extreme circumstances. “For a few weeks, the world has rehearsed a post-carbon world, a world not dependent on the car, a world that only consumes what is necessary, a world that only produces the essential, a contained and self-limited world, a world that understands what it is socially relevant and productive” (Fernandez, 2020). Such a context can be used as a testbed not only of environmental changes but also of a desired potential future city with other types of mobility (maybe the city without petrol cars) and different behaviors and proxemics (social distancing). The soundscape is a significant first intangible tester of these changes in a new imposed situation as it happens to be the COVID-19 pandemic. Until now, the tangible–intangible duality has been used for two purposes, not far from the city ecosystem. One is in the field of marketing, in the form of tangible and intangible assets. Another is the field of cultural heritage, which involves physical constructions that intertwine with intangible values and social traditions. In the latter, the “intangible” has already earned a certain rank as a formal category. Our goal should be to broaden the term and to define as “intangible” any feature of the city fabric that is not directly physical. This includes the soundscape.

The present study responds to calls for contextual specificity in soundscape research (Hong and Jin, 2015). Through a case study of the Getxo site, we aim to identify how people’s activities and their perception of the acoustic environment can determine whether some aspects of the soundscape have indeed changed significantly during the time of the pandemic lockdown.

We framed the study within the overarching idea that, due to the pressures of the COVID-19 lockdown restrictions, humans and animals would respond by changing their activities and behavior and that the soundscape would indicate the character and magnitude of those changes. This directed our attention toward five assumptions formulated at the beginning of the data collection. Firstly, that loudness would decrease; secondly, sounds linked to machinery and human interaction would decrease; thirdly, human outdoor activity would decrease; and fourthly, birdsong would increase. A fifth assumption emerged from observations, namely, that personal mobility devices (scooters and bikes) would be increasing.

The next section recapitulates the development of the pandemic and lockdown restrictions in response and describes the site of our study. This is followed by a *Methods* section in four parts; outlining the procedures for collecting audio recordings; making diary observations; conducting two analyses by an expert group, which allowed the construction of a Taxonomy of Sound Sources; and extracting computational soundscape descriptors. We report integrated results and make comparisons in relation to the lockdown phases. In the *Discussion*, we return to the five assumptions and attempt to provide answers.

### Pandemic Lockdown Response Phases

In early March 2020, cases of COVID-19 started to be detected in Spain. Within 1 week, there were 589 confirmed cases and 10 deaths, and within 2 weeks, Spain recorded 7,753 cases and 288 deaths (Kassam and Burgen, 2020). The accelerating severity of the situation pushed the national government to proclaim a state of emergency. Learning from nearby countries that were “ahead of the curve,” notably Italy, strict measures were introduced on 14 March. The project presented in this article started immediately thereafter, on 15 March at noon, on the first day of official application of confinement measures in Spain. In the following weeks, the country moved through different phases of restriction to activity and mobility (see Table 1). At around noon every day, the first author made a 5-min audio recording of the sonic environment and made observations from the window of her residency.

**TABLE 1:** Description of each phase of lockdown in Spain.

Phase	Duration (date)	Duration (days)	Outline
Phase 1	15-03 to 29-03	Day 1 to Day 16	Restrictions to mobility and to activity.
Phase 2	30-03 to 22-04	Day 17 to Day 39	Further restrictions to mobility, all non-necessary activity suspended.
Phase 3	23-04 to 21-06	Day 40 to Day 100	Progressive release of mobility and activity restrictions, starting with children allowed outdoors 1 h a day.

Undoubtedly, the coronavirus causes death and a great deal of psychological suffering. The restrictive responses inflict traumatic changes to human lives, with businesses closing, plans being canceled, and the stress of being confined to staying at home. These changes tend to affect those most vulnerable in society the most. By the end of April 2020, most countries in the world had declared partial or total lockdown regimes, causing half of the world population to live in confinement (Sandford, 2020). These measures impose a drastic reduction in human activity, “causing multiple cities across the globe to simultaneously go into hibernation” (Weston, 2020).

There are subtle changes underway that may have less dramatic yet deep and long-lasting influence. The soundscape is both an indicator of environmental quality and a component of cultural identity. For example, one newspaper stated that “life has changed, too: The city no longer sounds the same. And that realization is as jarring as the sight of empty streets” (Bui and Badger, 2020). Others have commented how animals react to the changing environment, such as “birdsong, for instance, seems louder than ever before. Some birds are actually likely to be lower in pitch than before, since they have fewer cars, planes, jackhammers, and leaf-blowers to compete with” (Ro, 2020). As for marine life, “evidence of a drop in underwater noise pollution has led experts to predict [that] the crisis may […] be good news for whales and other sea mammals” (McVeigh, 2020). Reduced human activity affects not only the living conditions for animals but also might even impact the crust of planet Earth itself, as “seismologists are reporting less seismic noise, or vibrations in the Earth’s crust” (Ro, 2020).

As mentioned, Spain entered a several-weeks-long sequence of different lockdown phases on 15 March. Citizens are generally confined in their residencies; schools, shops, and other services are suspended; leisure and sports activities are forbidden. Restrictions to local, national, and international mobility vary. In this article, we refer to three phases defined by the severity of the restrictions. Table 1 briefly illustrates the restrictions to mobility and activity applied in each phase. Phase 1, which lasted approximately 2 weeks, initially allowed citizens’ mobility to reach their workplace while suspending any other activity. Phase 2 saw an increase in the severity of the measures with the total suspension of any non-necessary activity. We identify the beginning of Phase 3 with the ease of the most restrictive measures – notably, the permission for kids to spend up to 1 h a day outdoors – which preceded the launch of the so-called “Plan de Desescalada” (Consejos de Ministros de España, 2020) on 28 April.

A detailed analytical overview of the phases with restrictions applied in each phase as adapted by the government of the Basque Country (Mallo,2020a,b) is available in the Supplementary Material.

### Site of the Case Study

**FIGURE 1:**
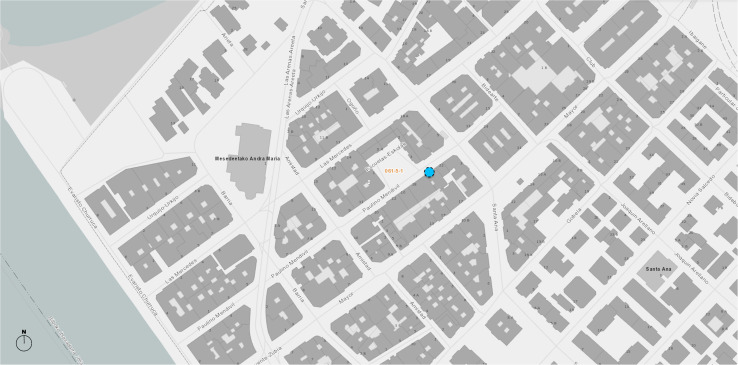
Map of the area surrounding the site.

The case study took place at Plaza de las Escuelas (coordinates 43.325777, −3.014046^1^). Our observation point (see Figure 1) is located in Las Arenas, a neighborhood in Getxo, which is a municipality on the estuary of the River of Bilbao. Getxo, a traditionally residential agglomeration with 77,000 inhabitants, lies 12 km from Bilbao, the largest town of the Basque Country. The area is gentrified with few high-rise buildings. Following a trend that is common all over Europe, it is home to an aging population where almost a quarter of the inhabitants are 65 years old or above. The economy of Getxo is essentially based on the third sector (services), which accounts for 92.4% of the turnover of the municipality, with a very weak presence of production from the primary and secondary sectors (EUSTAT, 2020).

In order to be able to analyze the soundscape variations from an urban spot that is representative of the city as a whole, we need an observation point that can record different layers of activity, granting that none of them overlaps and covers totally the others. From a mobility point of view, coexisting light traffic [pedestrian, bicycles, and personal mobility vehicle (PMV)] and traditional heavy traffic (cars and freight) must be present. As for human activity, public space should offer both a static gathering space (plaza or alike) and a transit. Regarding economic activities, the spot ought to host shops and bars, mirroring the usual premises of an average residential street of Getxo, including delivery services. Recordings should, in an optimal scenario, be taken from a first floor to avoid distortions and to better engage with street activity.

Paulino Mendívil is a pedestrian road, allowing only for limited vehicle access (08:00 to 12:00 for deliveries) in all its 180-m length. It intersects with Andres Larrazabal street, forming a pedestrian cross that is encircled by the wheel traffic of the surrounding roads. Our observation point is halfway (95 m) to both wheel traffic roads and on the point of convergence (gradient) of the isophonic lines defined by the “Noise White Paper” of the municipality of Getxo (see *Acoustic Environment* section). Hence, we can observe the consecutive layers of sound coming from heavy traffic but also record and analyze dynamic and static public space occupation on the plaza together with commercial activity on the ground floors. The observation point also gives the opportunity to record human activity related to work and leisure at a wide range of time without dealing with a too-dominating traffic noise. Nonetheless, being our observation point halfway to both extremes of the road (where car traffic is allowed), we still can identify motor vehicles flow if existing. The residence of the first author is also on a first floor, which enabled a perfect reference observation point for the study.

**FIGURE 2:**
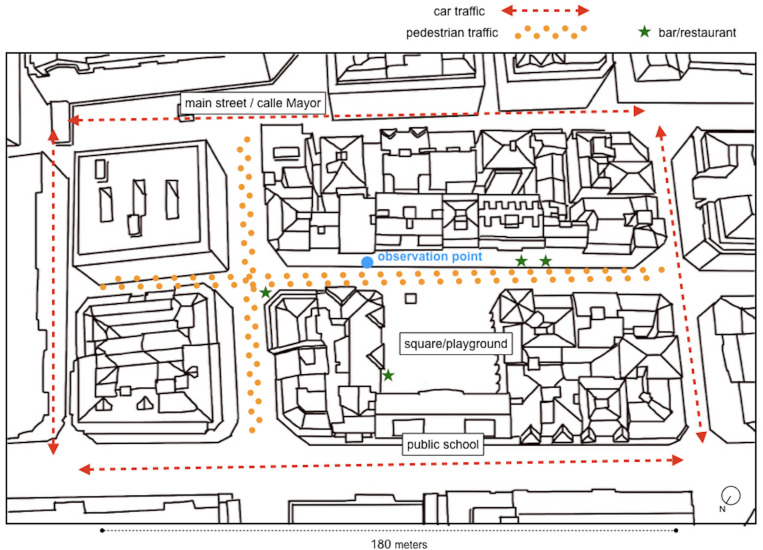
Urban blocks and traffic at the site. The site is mainly residential, with a public school overlooking the plaza. Almost half its area is dedicated to a playground.

**FIGURE 3:**
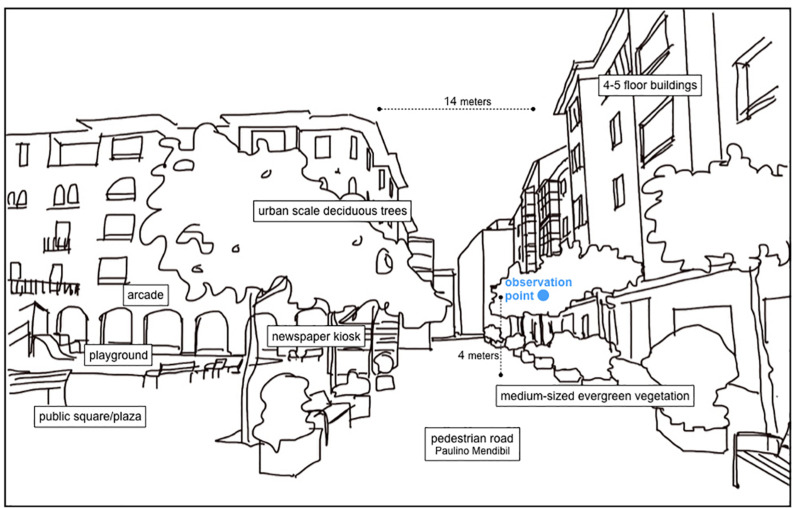
Urban elements and proportions at the site. The buildings in the street are mid-20th century, showing brick and stone constructions with a balanced composition of walls and voids. The average height of the building is five to six floors, including the ground floor.

With the exception of the children’s playground (see Figure 2), covered with rubber flooring, the rest of the surfaces are stone slabs. It is important to take these materials into consideration. Materials affect the acoustic properties and hence perception of sound (see, e.g., Lindborg, 2015). A double line of trees at both sides of the road offers a sound and visual cushion (Figure 3). This changing green environment also affects the cushioning and filtering of sound that the tree leaves account for. The present study takes place during the growing of leaves and blooming of trees, starting from no leaves at all in mid-March to full coverage at the end of May.

**FIGURE 4:**
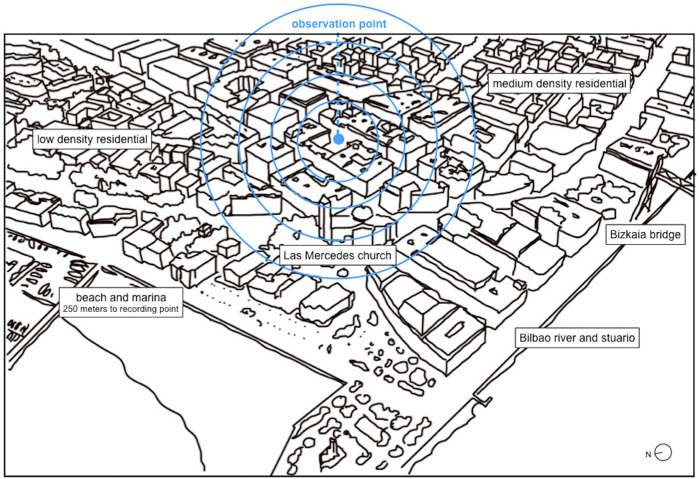
Spatial location of the observation point.

As Figure 4 illustrates, the site is only 250 m from the sea, which provides wind and the sonic presence of seagulls, waves, and boats. There is also a church tower with bells nearby, and the main activity road of the neighborhood (Calle Mayor, see Figure 2) is one block apart. The surrounding larger area is mainly residential.

The weather in Getxo is mild, with temperatures oscillating between a minimum of 6°C and a maximum of 22°C on a yearly average. The quantity of yearly rain precipitation is not low, going from a minimum of 50 to 170 mm. This means that outdoor activities are limited by rain and that architecture responds to this aspect, as we can see in the main plaza (next to the observation point, Figure 3) with an arcade (echoing sounds) surrounding the space and allowing urban life during rainfalls. Social life is intense, but squares and plazas are not constantly occupied, and usually people do not interact from their windows or balconies. Office work starts at 08:00–09:00, retail opens at 10:00, and lunchtime is late by European standards: at 14:00 on working days and 15:00 on weekends. Dinnertime also is late evening, at 21:00–22:00. Children attend school all day until around 17:00, when they join extracurricular activities or go to the playground if the weather allows. The local habit of joining family, friends, and colleagues for pre-lunch and pre-dinner drinks is particularly well established. Given the presence, at the site, of several bars and restaurants as well as an outdoor playground, the area of the case study tends to be crowded with children and adults during the hours that precede lunchtime and dinnertime.

### Acoustic Environment

The municipality of Getxo has shown great concern with the acoustic environment. The “Noise Map” (AENA, 2013) and the “Acoustic Zonification of Getxo” (Municipal de Getxo, 2016) are two white papers emanating from the “Basque Government Law of Noise” (Jefatura del Estado, 2003). The two white papers provide a benchmark on the acoustic environment in Getxo, focusing mainly on sound levels and noise. In particular, the second document identifies the location of the site of the present study as residential. For each type of zone, there is an acoustic quality objective (AQO). Three measures are defined for different parts of the day. Ld is the A-weighted level-equivalent sound pressure level (SPL) (in dB *re* 20 μPa) during daytime, 07:00 to 19:00. Similarly, Le is for evening time, 19:00 to 23:00, and Ln is for nighttime, between 23:00 and 07:00. For the neighborhood of Las Arenas, AQO is set at Ld = 65 and Le = Ln = 55. The goal of the municipality is to lower these limits by another 5 dB in future residential developments.

**FIGURE 5:**
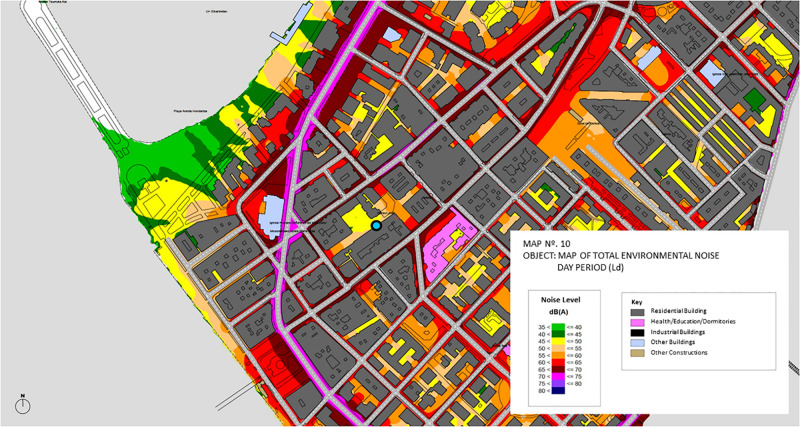
Excerpt from Map 3 of the Acoustic Zonification document, showing the ambient levels in the neighborhood around the site. Colors of blocks indicate zoning (residential, health/mixed, industrial, commercial, and other). Colors of urban arteries indicate noise levels. Image used with permission.

The Acoustic Zonification includes a “’Noise Map of Getxo” that indicates sound levels (Total Ambient Noise) and zoning in different parts of the city. A part of the map is shown in Figure 5. The observation point is inside an area where Ld is indicated to be in the range of 45–50. It is equidistant from areas characterized by much higher noise levels (Ld = 60–65) and areas with higher (55–60) or slightly higher (50–55) levels of total ambient noise. However, in measurements at 50 midday recordings over the 69-day period, we found the daytime level to be 56.3 dB (A-weighted level-equivalent SPL), while the nighttime level (mean across four separate recordings toward the end of the period) was 40.0 dB. Further research might show if the slightly higher daytime levels reflect a general effect of the lockdown on changed behaviors by users.

## Materials and Methods

### Audio Recordings

Audio recordings were made at the observation point (see *Site of the Case Study* section). We used a Zoom H4 recorder with integrated stereo microphones positioned at a 90° angle, with a sample rate of 48 kHz and bit depth of 24 bits. The recorder was placed in the same spot every day, at a windowed balcony, with the window open. The pre-amplifier level was set at mark 68 for the first 10 days and then adjusted to mark 65. No other position or amplifier adjustments were made. Calibration recordings and SPL measurements were made to account for this slight difference in the computational extraction of loudness (Section “Computational Descriptors” below).

Forty-two of the 50 recordings were taken between 11:45 and 14:00, six between 16:00 and 18:00, and two between 19:15 and 22:00. While there might be reasons to exclude the late-hour recordings, initial analysis revealed that the statistical results did not change in any significant way by their exclusion. Therefore, the analysis proceeded with the full set. The audio files were scrutinized and trimmed, so that a clean section of adequate duration could be taken from the beginning of each file. The excerpts needed to be sufficiently short to avoid fatiguing the volunteers in the annotation exercise (see below) yet long enough to provide representative data. We decided on a target duration of 120 s, though in three cases shorter files (90, 45, and 38 s) were deemed acceptable for inclusion. Compressed versions of the 50 soundscape recordings are included as Supplementary Material to the article.

### Diary Notes

Diary notes, in the form of short catchphrases, were written at the time of soundscape audio recordings by the author of the recordings as a spontaneous collection of traces inspired by the experience itself. Such observational field notes (Flick, 2014) express the “researcher’s own thoughts, feelings, impressions and insights” (Maharaj, 2016) and highlight elements that from time to time appeared to be the most striking. Occasionally, they serve as a poetic deepening of the acoustic and visual experience. Furthermore, they tend to express overall changes in the neighborhood as witnessed by the researcher that neither visual nor sonic material in their own fully capture. Gehl and Svarre (2013) identify several tools to extract a deeper knowledge of the use of public space in urban research. Among others, they describe the action of keeping a diary that can “register details and nuances about the interaction between public life and space, noting observations that can later be categorized and/or quantified” (idem, page 24).

The diary notes and recordings have been continuously shared on the blog of the first author (Lenzi, 2020) and via social media channels. The complete list of diary notes is included as Supplementary Material.

### Soundscape

We are interested in the relationship between perceived sound sources and the perception of the soundscape as a whole (ISO, 2014). In order to capture the human perception of the acoustic environment at the site, we gathered a group of 14 experts in soundscape, music, and architecture and tasked them to analyze the set of soundscape recordings. Two separate procedures were carried out: evaluations of soundscape quality and annotations of sound sources. The three authors participated as well, two of whom having knowledge about the site. Among the others, six were professionals in architecture or music and five were graduate students in these fields. The median age in the group was 40 years, in a range between 24 and 53, with equal numbers of men and women. Having received full information all declared consent in writing before commencing, the two tasks were completed several weeks apart. The instructions are available in Supplementary Material to this article. The collection of diary notes and soundscape recordings did not require an ethics approval from the institution of the first author. The procedures for evaluation and annotation were approved by the Research Ethics Committee of City University of Hong Kong (ref. 13-2020-08-E).

#### Evaluations of Soundscape Quality

**FIGURE 6:**
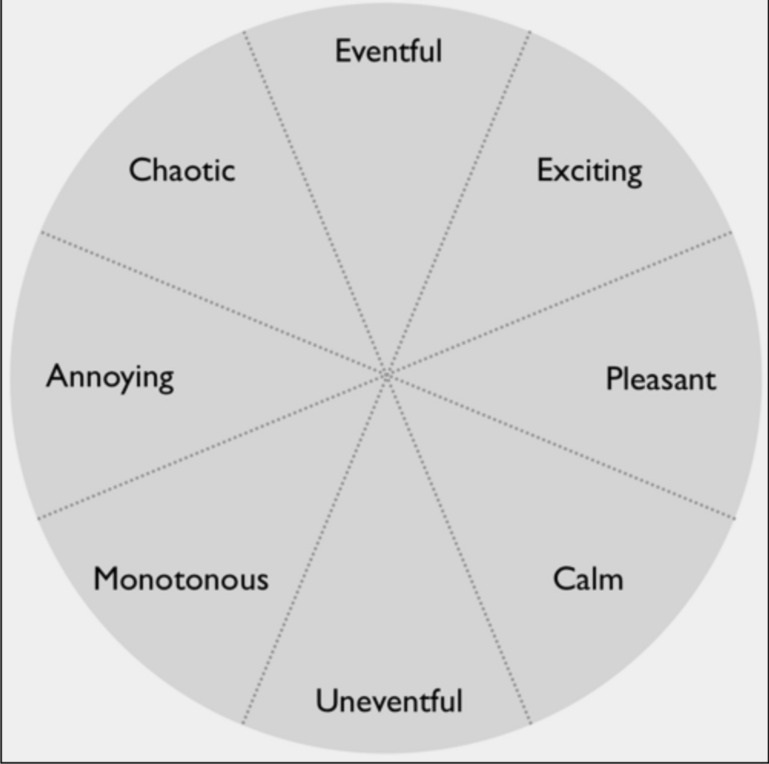
Graphical user interface for the ratings of soundscape quality. The eight adjectives in the circumplex yield two dimensions, Pleasantness and Eventfulness, which are considered to be orthogonal.

Eleven experts rated the 50 soundscape recordings using an adapted version of the Swedish Soundscape Quality Protocol (SSQP; Axelsson et al., 2010). This task calls for a mode of semantic listening (Chion and Gorbman, 2009; Lindborg, 2019) and took just under 2 h to complete. The SSQP includes eight adjectives: pleasant, exciting, eventful, chaotic, annoying, monotonous, uneventful, and calm. These words were originally selected for representing equidistant and equally strong semantic concepts, spanning a circumplex with the horizontal axis labeled Pleasantness and the vertical axis Eventfulness. In our adapted version, the circumplex and adjectives are presented as shown in Figure 6. While listening to the recordings (in individually randomized order), the rater continuously pointed with the computer mouse to the adjective that “best described what you feel the soundscape is like” (Axelsson et al., 2010). From the response time series, the mean angle and distance from the center were calculated for each of the 50 soundscapes across raters. This yielded values for Pleasantness and Eventfulness for each soundscape recording. The agreement was good among the expert raters (*N* = 11), as indicated by Cronbach’s alpha = 0.87 for Pleasantness and 0.83 for Eventfulness.

#### Annotations of Perceived Sounds

Twelve experts scrutinized the same set of soundscape recordings to identify individual sound events and describe them according to their perceived source. Labels were recorded as free-form text, almost all consisting of three words or less, along with begin and end times. This task called for a mode of causal listening (Chion and Gorbman, 2009; Lindborg, 2019) and was considerably more time-consuming than the previous task. Three of the experts completed the whole set of 50 soundscapes in 4–5 h. Others completed on average 26; no one less than 20. Each made between 7 and 22 labels per recording (median = 14), producing a total of 5,581 annotations of perceived sounds.

The labels were pre-processed by removing non-letter symbols (such as question marks, citations, parentheses, and trailing spaces) and transcribing to lowercase. There were a total of 10,441 individual words, out of which less than a thousand were unique. The 23 most common were as follows: birds (4.3%); voice (3.3%); door (3.0%); bird (2.8%); car (2.6%); voices (2.5%); dog (2.2%); talking (2.0%); human (1.7%); distant, child (1.6%); traffic (1.5%); chirping (1.4%); man, noise, male (1.3%); woman (1.2%); barking (1.1%); footsteps, closing (1.0%); and passing, female, children (0.9%).

**FIGURE 7:**
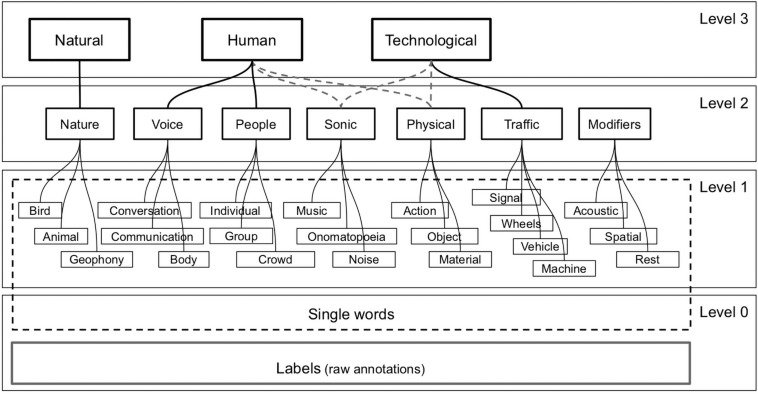
Schematic representation of the Taxonomy of Sound Sources.

A taxonomic classification with interconnected levels can serve as a bridge between a detailed description and a holistic description. To build a taxonomic classification of perceived sounds, we chose an empirically grounded approach (Scott-Ram, 1990; Atkinson et al., 2000; see also Lindborg, 2016). With the frequency counts in mind, we sorted each of the original 5,581 annotations within exactly one of the following 22 categories constituting Level 1: bird, animal, geophony, conversation, communication, body, individual, group, crowd, music, onomatopoeia, noise, action, object, material, signal, wheels, vehicle, machine, acoustic, spatial, and rest. Keywords for inclusion or exclusion speeded up the process so that ∼55% could be automatically matched, while the remainder required individual attention. Next, we developed Level 2 of the taxonomy and automatically sorted (by keywords) each of the Level 1 categories into exactly one of the Level 2 categories: nature, voice, people, sonic, physical, traffic, and modifiers. Finally, we defined three categories in Level 3 Natural, Human, and Technological, to correspond to the typology for sounds in soundscape first advanced by Schafer (1977) and developed by, e.g., Krause (2008) and Axelsson et al. (2010). This process yielded the taxonomic classification illustrated in Figure 7, with examples given in Table 2. The agreement was very good among the expert raters (*N* = 12), in terms of the proportions of Natural, Human, and Technological sounds in their annotations of soundscapes, as indicated by Cronbach’s alpha = 0.92, 0.95, and 0.93 for the three categories, respectively.

**TABLE 2:** Part of the Taxonomy.

Annotations, raw (random sample)	Keywords/inclusion	Keywords/exclusion	Level 1	Level 2	Level 3
Birds and voices almost muttered, bird chirps, dog roaming, bird chirp, chirp birds, bird chirps, birds plenty, birds intense, maybe it is raining, cat meow, dog yelp, dog’s footsteps, birds faint, cocorita, bird chirping, slightly differently, dog walking,	animal, bark, bees, bids, bird, birs, brids, burds, cat, chirp, cocorita, crow, dog, flap, fly, gull, gust, gut, insect, miaow, mosquito, nightingale, pigeon, rain, sea, seagul, seagull, trill, tweet, waves, wind, wings, yelp	Crowd, train, winding, window	Bird (856), animal (269), geophony (36)	Nature (1189)	Natural
Chatting + children voices, people talking and birds, woman talking with man, voice, female, child talking, human male voices chatting, adult footsteps, steps, mam speak with her children, Girl shout, woman or child humming, kid’s voice, playful, woman voice, children voices, clear conversation, Man talks, kids voices (playing, shouting), female voices,	adieu, burp, bye, chat, chuckl, clap, complain, convers, coo, cough, count, creaing, cry, dialog, exclaim, exhalation, foot, footstep, giggl, heel, humm, laugh, nose, running, scream, screem, shout, shriek, singing, sneez, sole, speak, step, stpes, talk, tantrum, throat, ululat, vioce, vocal, voice, walk, whin, whisper, whist	Passing, passing, closing, rising, creasing, machine, music, thump, traffic, scooter, wheel, cart, passing	Conversation (358), communication (826), body (265)	Voice (1490)	Human
children, indistinct voices, baby babbling, little scream, rather loud female voice, many voices, human voice (close), whining/seagulls, distant, young child, human voice, kids, shouting, voice, male, close, kid’s voice (faint), amplified female, children chasing, human voices, close, human whistle, man sings	adult, adults, anthropic, babbl, babies, baby, backg, boy, chid, child, children, crowd, din, faemale, family, father, femail, female, folk, girl, group, humans, kid, kids, male, man, market, mather, men, mom, mother, mumbl, murmur, neighbors, neighbors, owner, parents, people, person, police, somebody, someone, women	Human, instrument, movement, winding, descending, sliding, bounding, receding	Individual (922), group (147), crowd (91)	People (1356)	
Click, dops-like, human activity sounds, starts music, creaking, blinds closing sound, human activity/dishes, melody continues, clicking, sound by TV, scan sound, background buzz, clacking, radio, indistinct human and non-human noise, squeaking, melody, nice, like playing background music, indistinct non-human, clink, distant	accordeon, airflow, bang, bash, boom, boum, bump, burst, buzz, carillon, choir, chor, chorale, clack, clang, clash, classic, click, cling, clink, cordion, crash, creak, crink, cump, doum, drip, flute, guitar, harmonica, howl, hum, instrument, jing, jingl, knoc, major, melod, music, nois, organ, patter, pjoff, puff, radio, rhythm, rumbl, sbam, scan sound, schreech, scratch, screech, shot, shrill, slam, snap, song, sound, sqee, squeak, squee, squirr, squoink, swish, teardrop, thud, thump, tick, tone, trumpet, tv, undefined, undetermined, unidentified, vent, whir, whosh, wind, woosh		Music (116), onomatopoeia (517), noise (475)	Sonic (1108)	NA
Door slam, bash car door, doorlock again, cutlery plates, gate, Something swipes, rattling, high pitch, unlocking, car door, hit, indoor, keys jingling, hits (glass falling), objects bashing (faint), scratching of something on the ground and, pluck, cups, plates, 2 rattles, hammering	activ, ball, bike, blind, bottle, bounc, break, buck, can, chain, chair, crockery, cup, cut, cutlery, dish, door, drag, driv, drop, fall, flutt, gate, glass, hammer, hit, house, iron, item, key, kick, kitchen, knick, lagguage, lock, material, metal, moving, newspaper, object, paper, pladtic, plastic, plate, play, pluck, pull, rattl, roll, rubb, saw, scissor, scrap, shak, shaker, shuffl, shut, shutter, sifting, solder, something, spoon, start, steel, stomp, stone, strap, sunblid, swip, tennis, throw, tool, toy, trunk, water, wood, work, wrapper	Scan	Action (557), object (564), material (106)	Physical (1243)	NA
Stroller wheels, van parked, engine still running, bus slowing down, beep, bike in water puddle? Tram arrival, engine truck close, ambulance siren, indistinct voices/busy people chatting, siren faint, bus whistling breaks(?), trolley little wheels - metallic, plane, car brake, battery car, bip, no-human, gear, playing with skate	alarm, ambulance, atm, beep, bell, bicycle, bike, bip, brakes, braking, bus, calls, car, cart, chart, chopper, church, clacson, claxon, construction, delivery, drone, electr, engine, gear, hawking, honk, horn, jet, machine, mechanic, message, motocycle, motor, motorbike, motorcycle, mower, parked, phone, plane, raffic, revs, revv, ringtone, road, scooter, signal, siren, skate, skatebaord, skating, stroller, traffic, train, tram, trolley, trolly, truck, van, vehicle, warning, weel, wheel	Atmosphere, motorbike, cart, door, key, carrousel, carillon	Signal (259), wheels (162), vehicle (562), machine (33)	Traffic (1062)	Technological
Mic handling noise, bump on mike, microphone manipulation, around eight beats, regular on a pitched drum (blank), noise on microphone, undetermined urban noise, croak/fart	accelerat, acoustic, approach, arrival, audible, away, backg, behind, between, bit, blank, circular, city, clear, clos, creasing, departure, distanc, distant, doppler, echo, exotic, faint, far, foregr, freq, from, front, hard, heavy, high, hollow, indistinct, indoor, intense, large, light, little, loud, louder, lound, low, medium, mic, mike, mobile, movement, moving, muffl, near, open, passing, past, pitch, quiet, reced, reverse, soft, soundscape, sparse, street, strong, surface, sustain, toward, undistinct, undistinguished, urban, very, volume, weak	Rhythmic	Acoustic (445), spatial (636), rest (45)	Modifiers (1129)	NA

*In this example, rows correspond to the seven categories of Level 2. The leftmost column shows a random sample of the labels exactly as written. They are filtered through Level 0 (not shown) and auto-matched via the inclusion and exclusion keywords to aggregate into Level 1. The procedure is repeated to yield Levels 2 and 3, shown in the last two columns. Numbers in parentheses are counts.*

Note that unique associations between categories in Level 2 and Level 3 could not be made. For example, a perceived sound with a label including the word “music” might refer to a recording played on a radio and thus sort under Technological (Axelsson et al., 2010). Or it could be someone playing an instrument or singing and thus evidence of someone’s action with an object or their own body and thus sort under Human. Interpretative challenges such as these point to the difficulty of marrying a cladistic taxonomic classification (bottom-up) with a previously given typology (top-down). A datafile including all the annotated labels and levels of the Taxonomy of Sound Sources is available in the Supplementary Material.

From the taxonomy, we report results on four descriptors.

*Natural*, which relies on annotation of perceived sound sources that are related to birds of different kinds, as well as insects and geophony, water, waves, and so forth. Despite there being good reasons why domestic animals should not be categorized as part of the biophony (Schafer, 1977), we decided to include dog barks in this category to keep the taxonomy parsimonious.

*Human*, which covers a large range of sounds perceived to be produced by the human body, i.e., footsteps, speaking, and other vocalizations such as coughing and laughter. Please note that we decided to exclude music instruments and indeed any kind of sonic objects that might be manipulated by humans, since the source of such sounds is outside of the human body.

*Technological*, which includes sounds produced by machines, tools, cars, traffic, and so forth, a.k.a., technophony. While being propelled by a motor or an external energy source is a main characteristic of this category, we also included sounds from non-motorized wheeled vehicles such as bicycles, skateboards, and delivery carts.

*Perceptual normalized difference soundscape index* (pNDSI). We introduce a perceptual counterpart to NDSI (Kasten et al., 2012; see below), defined as

(1) pNDSI=(Natural-Human)/(Natural+Human)

where each variable is a time series generated by the taxonomy. With Natural and Human in the range [0–1], pNDSI is in the range [−1 to 1]. A value close to −1 indicates that the soundscape is dominated by sounds associated with humans, and a value close to 1 indicates prevalence of natural sounds.

### Computational Descriptors

We used six computational indices from soundscape and bioacoustics research (Sueur, 2018; Kang et al., 2019) obtained in R (R Core Team, 2020).

*NDSI* (Kasten et al., 2012; Sueur et al., 2019) estimates the level of anthropogenic disturbance on a natural environment with the ratio:

(2) NDSI = (b-a)/(b + a)

where b is the sound intensity in the 2–8 kHz range (where biogenic sounds are prevalent) and a is the intensity in the 1–2 kHz range (where anthropogenic or mechanical sounds are most prevalent). NDSI is scaled between −1 and 1, with 1 indicating pure biophony (cf. Sueur, 2018 p. 491–2; Remote Environmental Assessment Laboratory [REAL], 2020).

*Loudness* (N). The Loudness model (Zwicker and Fastl, 2013) was originally limited to sounds of short duration initiated, has been extended to model the perception of sounds with time-varying and complex spectra, and has been widely employed in soundscape studies (Axelsson et al., 2010; Davies et al., 2013; Lindborg, 2015; Aletta et al., 2016a; Lindborg, 2016; Anikin, 2017; Kang et al., 2019).

*SPL*. A-weighted SPL in dB *re* 20 μPa is reported to facilitate comparisons with other research.

*Loudness variability* (N_10__–__90_) is the difference between the loudness exceeded 10% of the time and that exceeded 90% of the time. While the former captures short and loud sounds, the latter captures the background. The range indicates the amount of foreground sources emerging from the background (Axelsson et al., 2010; Lindborg, 2015, 2016).

*Acoustic richness* (AR) is calculated from amplitude (M) and acoustic entropy (Ht) and ranked over several files (Sueur et al., 2019). M is scaled between the median Hilbert amplitude and the maximum. Ht increases with signal entropy, or heterogeneity, so that a higher value indicates a richer acoustic environment.

*Acoustic complexity* (ACI) is an index based on the “observation that many biotic sounds, such as bird songs, are characterized by an intrinsic variability of intensities, while some types of human generated noise (such as car passing or airplane transit) present very constant intensity values” (Pieretti et al., 2011 p. 869; Sueur et al., 2019).

## Results

Table 3 gives mean values of the soundscape descriptors determined from evaluations and annotations by the expert group (*N* = 15) and extracted computationally. Cross-correlations for Pleasantness and Eventfulness against other variables were calculated using Spearman’s rank-order correlation (since variable distributions were non-normal) and interpreted after Dunn–Šidák correction for each pairwise comparison significance level (α = 0.0022, for 23 comparisons at α_*FWE*_ = 0.05). For Pleasantness, there was a significant positive association with the amount of perceived Natural Sounds (ρ = 0.44, *p* = 0.0013) and negative associations with technological sounds (ρ = −0.44, *p* = 0.0015), SPL (ρ = −0.53, *p* = 0.0001), and Loudness Variability (ρ = −0.51, *p* = 0.0002; larger range correlated with less pleasant soundscape). Eventfulness was associated with SPL (ρ = 0.44, *p* = 0.0016), Loudness (ρ = 0.50, *p* = 0.0003), and Loudness Variability (ρ = 0.48, *p* = 0.0005). No other correlations were significant at the predetermined level. These findings are in line with previous soundscape research (e.g., Cain et al., 2008; Axelsson et al., 2010; Davies et al., 2013).

**TABLE 3:** Mean values for 13 descriptors of 50 soundscapes.

Days	Lockdown	Pleasantness	Eventfulness	Natural	Human	Technological	NDSI	pNDSI	Wheels vs. Vehicle	Loudness (sone)	SPL (dBA)	Loudness variability	Acoustic complexity	Acoustic richness
1	Phase 1	–0.62	–0.07	0	0	1	–0.13	0	–1.00	2.22	62.4	4.61	12,993	0.02
2	Phase 1	–0.02	–0.58	1.13	0	0.25	–0.35	1	0	0.79	55.6	0.36	12,762	0.49
3	Phase 1	–0.48	0.33	0.2	0.3	0.3	0.67	–0.20	–0.10	1.09	58.3	2.4	13,651	0.08
4	Phase 1	–0.62	0.08	0	1	0.29	–0.12	–1.00	–0.29	1.03	57.6	0.76	13,315	0.61
5	Phase 1	–0.44	0.28	0	1.17	0.33	–0.60	–1.00	–0.17	0.85	55	0.32	13,028	0.08
6	Phase 1	–0.51	–0.31	0	0.57	0.57	–0.66	–1.00	–0.57	0.65	57.8	0.34	13,028	0.26
7	Phase 1	–0.53	–0.21	0	1	0.17	–0.71	–1.00	–0.17	0.71	55.4	0.75	12,963	0.17
8	Phase 1	0.46	–0.27	1	1.14	0	–0.49	–0.07	0	0.52	51.3	0.26	13,409	0.11
9	Phase 1	–0.37	0.45	1	0.33	0.56	–0.63	0.5	–0.44	1.88	58.9	1.63	13,470	0.28
10	Phase 1	–0.60	–0.14	0.63	0.88	0.63	0.51	–0.17	0	0.59	53.2	0.9	13,314	0.11
11	Phase 1	–0.46	0.4	0.89	0.22	0.56	–0.19	0.6	–0.44	1.81	56.1	0.93	14,993	0.11
12	Phase 1	–0.56	–0.01	0.13	1.25	0.13	–0.44	–0.82	0	0.74	56.4	0.38	13,442	0.47
13	Phase 1	–0.53	–0.16	0.25	0.88	1	–0.43	–0.56	0	0.6	53.1	0.74	13,292	0.02
14	Phase 1	0.31	–0.55	1.2	0	0.8	–0.41	1	–0.80	0.36	50.7	0.33	12,980	0.05
15	Phase 1	0.07	–0.53	0	0.56	0.22	–0.27	–1.00	–0.22	0.9	50.9	0.51	14,294	0.08
16	Phase 1	–0.58	0.08	0.57	0	0.86	–0.74	1	0.43	1.79	61.3	1.73	13,126	0.49
17	Phase 2	0.02	–0.61	0.63	0	0.5	–0.56	1	–0.25	0.56	53.6	0.22	12,744	0.35
18	Phase 2	–0.50	0.25	0.11	0.33	0.78	–0.57	–0.50	0	1.65	60.4	2.04	13,150	0.24
19	Phase 2	–0.23	–0.60	0	0	0.64	–0.44	0	–0.64	0.28	50.9	0.62	12,839	0.01
20	Phase 2	0.37	–0.46	0.3	0.9	0.2	–0.27	–0.50	–0.20	0.26	49.7	0.14	13,408	0.05
21	Phase 2	–0.14	–0.55	0.57	0.57	0.43	–0.52	0	–0.29	0.79	52.8	0.27	13,469	0.07
22	Phase 2	0.2	–0.54	1	0.5	0.17	–0.24	0.33	0	1.21	53.6	0.45	14,548	0.17
23	Phase 2	–0.58	0.25	0.8	0.7	0.2	–0.24	0.07	–0.20	1.66	57.4	0.98	13,754	0.37
24	Phase 2	0.47	–0.35	0.3	0.6	0.2	–0.45	–0.33	–0.20	1.5	56.2	0.64	13,660	0.02
25	Phase 2	–0.09	0.57	0	0	0	–0.54	0	0	0.71	52.1	0.37	13,277	0.04
26	Phase 2	0.31	–0.53	0.29	0.71	0	–0.30	–0.43	0	0.16	47.4	0.07	12,845	0.02
27	Phase 2	–0.36	–0.46	0.71	1	0	–0.60	–0.17	0	0.5	51.7	0.39	13,477	0.1
28	Phase 2	0.64	–0.23	1	0.71	0	0.67	0.17	0	0.27	49.2	0.22	13,736	0.03
29	Phase 2	–0.57	–0.08	0.29	1.29	0.57	0.24	–0.64	–0.43	0.57	53	0.36	13,833	0.15
30	Phase 2	–0.22	–0.59	0.33	1.33	0.67	–0.27	–0.60	–0.17	1.22	54.1	0.44	13,700	0.27
31	Phase 2	–0.40	–0.41	0.57	0.57	0.43	–0.50	0	–0.43	1.44	56.8	0.6	12,950	0.08
34	Phase 2	–0.10	–0.59	0.22	0.22	0.44	–0.46	0	–0.44	0.72	55.5	0.34	13,507	0.44
35	Phase 2	0.55	0.31	0.83	0.33	0	–0.11	0.43	0	0.61	52.9	0.21	13,475	0.23
37	Phase 2	–0.49	–0.21	0	0.44	0.56	–0.39	–1.00	–0.33	0.76	56.9	0.31	13,307	0.65
38	Phase 2	0.41	0.46	1	0	0.43	0.53	1	–0.29	0.76	54.9	0.41	13,837	0.38
39	Phase 2	0.47	–0.33	0.86	0.57	0.14	–0.02	0.2	–0.14	0.66	52.3	0.42	13,849	0.15
41	Phase 3	–0.52	0.33	0.5	0.13	0.88	–0.54	0.6	0.13	2.15	62.2	2.31	13,415	0.13
46	Phase 3	–0.12	0.61	1	1	0	0.01	0	0	1.63	57.3	0.78	13,968	0.44
47	Phase 3	–0.53	0.4	0	1.38	0.38	–0.26	–1.00	0.25	7.55	66.7	6.89	14,424	0.22
48	Phase 3	–0.30	0.55	0.17	1.17	0.17	–0.51	–0.75	–0.17	3.09	60.2	2.94	14,860	0.38
49	Phase 3	–0.64	0.37	0	0.33	0.67	–0.59	–1.00	0.67	2.3	60.6	2.3	13,871	0.45
51	Phase 3	–0.39	–0.49	0	1	0.11	–0.29	–1.00	0	1.61	57.2	0.76	13,987	0.61
52	Phase 3	–0.62	0.03	0	0.67	0.11	–0.24	–1.00	0	0.72	49.8	0.43	14,499	0.04
54	Phase 3	–0.36	0.44	0	0.7	0.1	–0.61	–1.00	0	1.35	55.3	0.86	13,701	0.14
56	Phase 3	–0.31	0.48	0	1	0.29	–0.55	–1.00	–0.14	1.01	55.6	0.57	13,266	0.35
60	Phase 3	–0.30	0.45	0.33	1	0.33	–0.47	–0.50	0.17	1.43	57.1	0.94	13,549	0.29
62	Phase 3	–0.45	0.41	0.25	1	0.13	–0.43	–0.60	0	2.1	56	0.65	13,968	0.45
64	Phase 3	0.02	0.54	0.13	1	0	–0.22	–0.78	0	0.69	53.2	0.39	14,037	0.26
65	Phase 3	–0.56	0.15	0.33	0.56	0.33	–0.25	–0.25	–0.22	0.61	52.5	0.2	13,769	0.21
69	Phase 3	–0.33	0.54	0	1.14	0.29	–0.49	–1.00	–0.29	2.3	59.6	1.25	13,964	0.58

*Pleasantness and Eventfulness (evaluations by expert group, *N* = 11) are in the range [−1 to + 1]. Natural, Human, Technological, pNDSI, Wheels vs. Vehicles (annotations by expert group, *N* = 12), SPL in A-weighted dB re 20 μPa, Loudness and Loudness Variability in sone. For Acoustic Complexity and Richness, see Section 2.5.*

**FIGURE 8:**
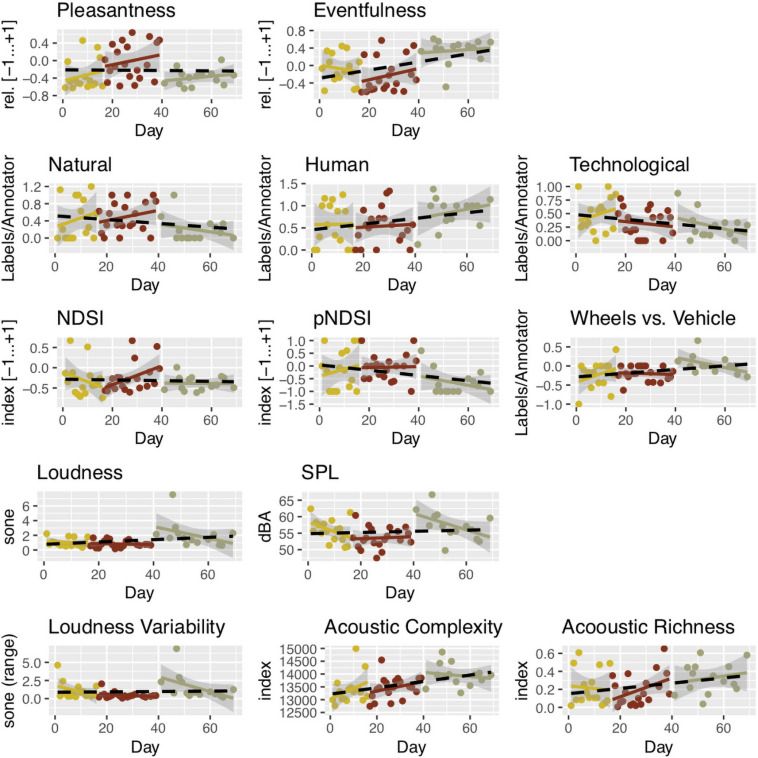
Six computational and seven perceptual descriptors over the 69-day period under study. Loess regressions are given for each of the three lockdown Phases, with smoothing *f* = 0.67 and 95% confidence interval. To facilitate interpretation, a black dotted line indicates the overall linear regression, but note that non-parametric statistics were used in evaluations of descriptor change over time. Image produced with ggplot2 (Liu and Kang, 2016; Wickham et al., 2016).

We proceeded with an analysis of the change over time for the 13 descriptors. A multivariate analysis of variance (MANOVA) revealed that there were differences in the data [Pillai’s trace = 0.52, *F*(13) = 3.09, *p* = 0.0037^∗∗^]. Spearman’s ρ was used to evaluate univariate correlations against a dummy variable for time, i.e., the 69 days. Results are given in Table 4, and Figure 8 plots the development of the variables against the three main phases of the lockdown.

**TABLE 4:** Correlations between 13 perceptual and computational soundscape descriptors and a dummy variable for time.

Descriptor	S	ρ	p
Pleasantness	18,580	0.108	0.45
Eventfulness	12,942	0.379	0.007**
Natural	23,587	–0.133	0.36
Human	15,342	0.263	0.065.
Technological	26,751	–0.285	0.045*
NDSI	19,872	0.0458	0.75
pNDSI	25,947	–0.246	0.085.
Wheels vs. Vehicle	14,637	0.297	0.036*
Loudness (sone)	16,442	0.21	0.14
SPL (dBA)	20,736	0.00427	0.98
N10m90	19,764	0.0509	0.72
Acoustic Complexity	9,382	0.549	0.00005***
Acoustic Richness	15,053	0.277	0.051.

S is the value for Spearman’s test; ρ is the rank-order correlation; p is the probability, if the null hypothesis of independence were true, of a result as extreme as the one obtained. By convention, a *p*-value lower than 0.05 indicates a significant association between variables. Asterisk codes for degree of significance: ***p < 0.001; **p < 0.01; *p < 0.05.

On-site diary notes were compared with perceived sound sources in Level 0 (original annotations) and Level 3 (categories of Natural, Human, Technological sounds) of the Taxonomy. This highlighted correspondences between the elements of the soundscape as they emerge from annotations and the subjective impressions as they were noted by the first author during the recording process throughout the different phases. Additionally, notes were compared with the evolution of the perceived quality of the soundscape (see *Evaluations of Soundscape Quality* section) over time and during specific days. See Table 5, which shows details for soundscapes captured on 8 of the 69 days.

**TABLE 5:** Correspondences between diary notes and annotations on selected days.

Day	Diary Note	Level 0	Level 3	Evaluation
4	I never noticed how much human voice resonated in the little plaza in front of our window. Interesting how the still image always look the same, day after day, while soundscape is so varied.	Birds (6), close loud hitting (3), whistling, bird chirps, people talking, voices, conversation between man and woman	Human (28), natural (15), technological (12)	Extremely annoying
14	Near silence.	Traffic passing (6), car driving by (3), bird (2), voices faint	Technological (28), natural (16), human (9)	Extremely calm
16	Making noise is feeling alive.	Birds (5), trolley, cart (4), stroller wheels (3), beep (2), dog bark, hits and bumps, music	Technological (32), natural (13), human (7)	Clearly annoying
19	In the silence, someone’s getting ready for lunch.	Child (3), glass bottles, object on a surface, car (2), child voice, clacking, hit plate	Human (23), technological (20),	Extremely uneventful
28	Birds, birds, birds.	Child (5), child voice (4),bird (3), birds, birds chirping	Human (39), natural (19), technological (7)	Extremely pleasant
39	Starting Sunday children will be allowed out. Is it excitement I am hearing in the air?	Birds (6), children talking, dog, human voices distant	Human (27), natural (26), technological (14)	Strongly chaotic
48	It’s Labor Day, little by little, people are taking back the streets.	Birds (8), bird (6), female, bird chirping (5), footsteps, child (4), talking, children shouting (3), children voices	Human (87), natural (23), technological (4)	Strongly eventful
60	After 2 months of (almost) daily recording the soundscape of the little plaza in front of my window, as the Basque Country rolls out what in Spain is called “Phase 1,” with small retailers, hairdressers, hospitality open and people happily and maybe unwisely taking the road, I decided to stop publishing - even though I’ll keep recording as we move toward “the new normality”. Good luck everybody, who knows what a brave new world is awaiting us out there!	Bird (8), child (6), bird chirping (5), children, children shouting, footsteps (4), kids scooter (3), baby (2)	Human (58), technological (21), natural (20)	Clearly chaotic

**FIGURE 9:**
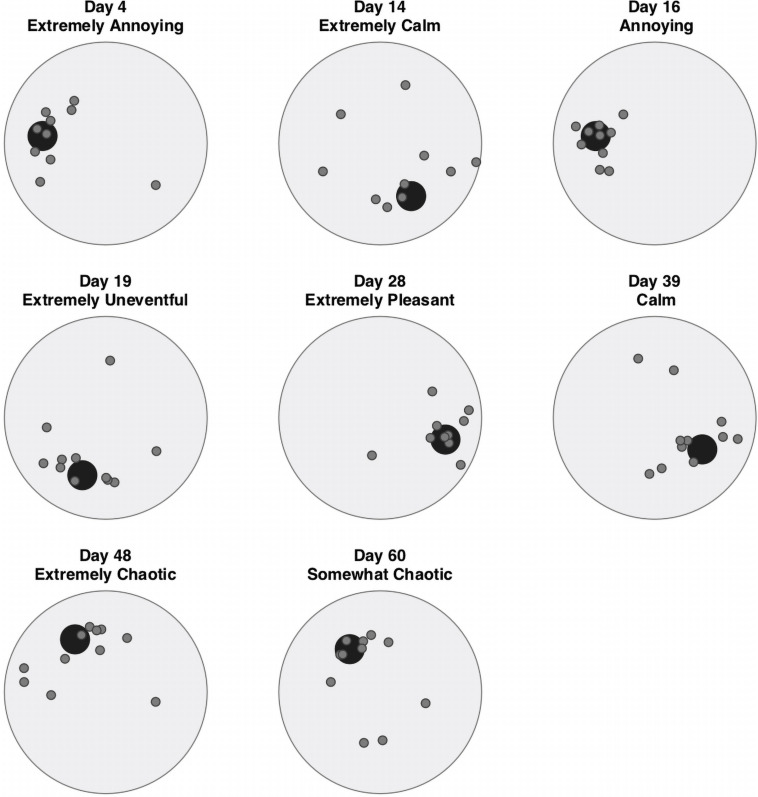
Pleasantness–Eventfulness circumplex for select days that are discussed in the article. See Supplementary Material for plots of all the days in the study. Small gray circles are the mean evaluations for each expert evaluator (*N* = 11) calculated as the circular mean of the continuous response and its mean distance from the center. The large black circle is the overall mean. The words in each subtitle indicate in which adjective sector the overall mean is located and its strength.

We found several correspondences between the author’s and evaluators’ perception of the soundscape in terms of Pleasantness and Eventfulness. Such correspondences are also sustained by the Taxonomy obtained from the annotations. On Day 4 of the confinement, the first author notes “I never noticed how much human voice resonated in the little plaza in front of our window. […]” Interestingly, in a Phase 1 marked by restrictions to human mobility and activity, the predominant perception of the soundscape of Day 4 is “extremely annoying” (see Figure 9). Annotations for the same day register a clear predominance of Human (annotated as a source of sound 28 times) over Natural (15) and Technological (12) sounds with the indication, at Level 0 of the taxonomy, of words such as “people talking,” “voices,” “whistle,” “human conversation,” “human voices,” and “dialog between man and woman.” On Day 14, an “Extremely Calm” day for soundscape Evaluation is described as “near silence” in the author’s notes, while annotations show a prevalence of technological sounds further described as “traffic,” “distant traffic,” and “car passing distant” (or “medium distance”). Day 16 stands out in the diary notes, in the context of the restrictions imposed by Phase 1 (“making noise is feeling alive”) as well as in the evaluators’ assessment (“clearly annoying”). Annotations register a clear predominance of technological (32) over Natural (13) and Human (7) sounds. As mentioned, Phase 2 was characterized by a strengthening of restrictive measures both to mobility and human activity. Day 19, the third day of Phase 2, is described as “Extremely Uneventful” by evaluators, while annotations highlight both human and technological sounds, the latter mainly referring to indoor activity (“glass bottle,” “objects on surface,” “hit plate,” etc.). Notably, the diary note for that day read “In the silence, someone’s getting ready for lunch.” Day 28 registers a clear predominance of Human (39) over Natural sounds. On the contrary, diary notes define it as a fully natural experience (“Birds, birds, birds.”). Evaluators describe it as “Extremely Pleasant,” leaving us in the doubt as to the perceptual dimension that is responsible for the pleasantness. The first day of Phase 3, when restrictions start to be lifted with kids being allowed outdoors for 1 h a day, marks a turning point in the lockdown diary notes (“Is it excitement I am hearing in the air?”). Evaluators define the same day as “Extremely Chaotic,” while Human and Natural sounds appear almost equally in the annotations (27 to 26). As restrictions are progressively lifted, Human sounds emerge as the prevailing source in the soundscape evaluations, as well in the diary notes. On Day 48, the author notes that “{…} little by little, people are taking back the streets.” Human sources are clearly predominant (87) over Natural (23) and Technological (4) sources. Evaluators define the soundscape as “Strongly Eventful.” On Day 60, toward the end of our recordings and with most of the restrictions lifted, with “[…] small retailer, hairdressers, hospitality open and people happily and maybe unwisely taking the road […]” and the soundscape is “clearly chaotic” with a prevalence of Human and Technological sounds over Natural source.

The results obtained through quantitative and qualitative analyses were interpreted in relation to the five assumptions we had previously made, to recall, (1) loudness will decrease; (2) machinery and human interaction will decrease; (3) human outdoor activity will decrease; (4) birdsong will increase; and (5) transport will shift from fuel vehicles to light mobility.

**FIGURE 10:**
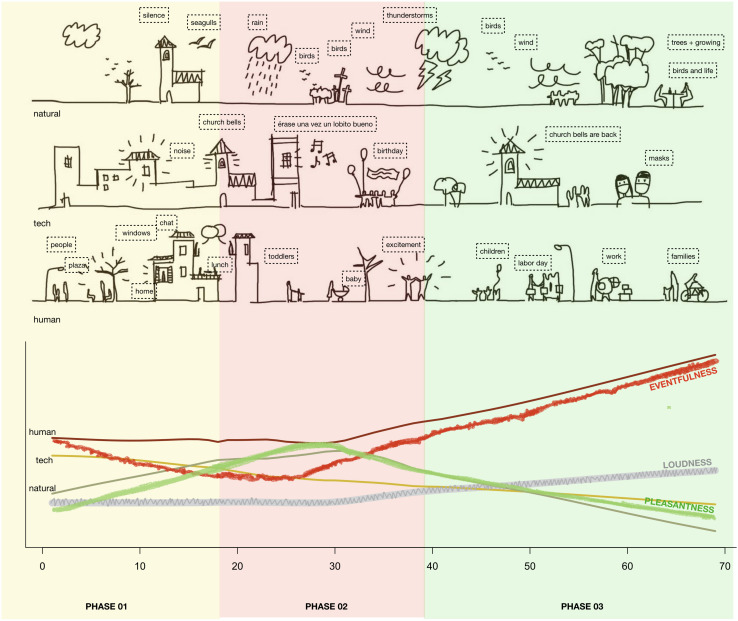
Timeline integrating select descriptors, condensed diary notes, and lockdown phases.

1. Over the 69 days covered in the study, overall Loudness did not significantly change (ρ = 0.21, *p* = 0.14 n.s.; likewise for SPL), contrary to expectations. Across the whole period, the average daytime sound level was 56.3 dBA, which is in line with the pre-lockdown municipality measurements of urban noise levels at the site (see *Acoustic Environment* section). The stable Loudness, contrary to expectations, might be explained by changes in people’s behavior, evidenced in the significant decrease in Technological sounds (ρ = −0.285, *p* = 0.045^∗^) being to a sufficient extent compensated by the increase in Human sounds (ρ = 0.263, *p* = 0.065). See Table 3 and Figure 8 (fourth row).
2. The decrease in Technological sounds during the time period, which was expected, indicates that the mandated restrictions on traffic circulation and human activities (with, for example, the temporary suspension of all construction works) had a noticeable influence on the soundscape at the site. Despite the site being a pedestrian area, the nearby main road is characterized by both private and public traffic circulation that can be heard from Calle Paulino Mendibil. Additionally, and perhaps due to the absence of direct sources in traffic noise at the site, the occasional construction works have a notable impact on the local soundscape. See Figure 8 (second row, right).
3. Human activity increased at the site during the studied period, as evidenced both in the diary notes and by the slight increase in sounds from humans (ρ = 0.25, *p* = 0.076). Human is a category in Level 3 that aggregates annotations from two Level 2 categories: Voice and People. The former showed no change over time (ρ = 0.16, *p* = 0.25 n.s.), while the latter increased (ρ = 0.35, *p* = 0.013^∗^). A closer look to the evolution in the perception of Human sounds during the different phases can help explain the results. Figure 8 (second row, middle) suggests that people’s activity level increased steadily throughout the time period, as evidenced by Human sounds, and was the highest in Phase 3. The diary notes substantiated this interpretation. The word “children” progressively appears from Phase 3 onward, when children started to be allowed outdoors after having been confined indoors in the first two phases.
4. The results in regard the expected increase in birdsong were not conclusive. We have already noted during the development of the Taxonomy that the most commonly annotated word overall was “bird,” which (together with “birds”) appeared in ∼7% of the original labels. The amount of perceived bird sounds did not change significantly over time (ρ = −0.09, *p* = 0.54 n.s.), as evidenced by annotations in the Level 1 category Bird. Neither did the Level 3 category Natural show a significant trend overall. However, inspecting Figure 10, there was an increase during Phases 1 and 2, followed by a much lower level in Phase 3. This might be explained by factors regarding avian activity: seasonal shifts, mating periods, and noon being something of a siesta time for birds See Figure 8 (second row, left). As for the computational descriptors, AR increased slightly over time (ρ = 0.28, *p* = 0.051), while Loudness Variability did not change appreciably (see Figure 8, fifth row), and the two NDSIs (NDSI and the proposed perceptually based pNDSI) showed similar patterns and no significant change overall (Figure 8, third row, left and middle).
5. Regarding a possible shift from fuel vehicles to light mobility (non-fuel vehicles), we analyzed two categories in the taxonomy, “Vehicle” and “Wheels,” both in Level 1. Recall from Table 2 that the former aggregates annotations about sounds from motorbikes, cars, and traffic, while the latter tracks sounds associated with bicycles, skateboards, and carts. The difference score (medians across 50 recordings) was significantly different from zero (Wilcoxon signed rank test *V* = 490, *p* = 0.0002^∗∗∗^), and there was a significant trend over time (ρ = 0.31, *p* = 0.03^∗^), giving evidence for the assumption of an increase of activities involving non-motorized “wheels” such as bicycles vs. motorized mobility. See Table 3 and Figure 8 (third row, right).

To sum up, we found that during the 69-day period during the lockdown at the site, overall loudness remained stable. There was a reduction in perceived sounds of machinery, especially traffic, and a shift from fuel vehicles to light mobility. Contrary to our expectations, human outdoor activity increased. There was no appreciable change to the amount of birdsong.

### Integrated Analysis

Finally, we present an integrated analysis of qualitative and quantitative results, diary notes, and phases of lockdown. It takes the form of the diagram shown in Figure 10, aiming to capture the essentials of the multifaceted aspects of our collaborative case study. Diary notes and Level 3 taxonomic categories (Human, Natural, and Technological; described in the *Methods* section) were organized by phases and further compared with the perceptual analysis results and with Loudness. We analyzed the text of each Diary note in order to assign it to one of the three categories, where possible. Associations were made based on human sounds, i.e., perceived to be produced by people. This includes voices, footsteps, and laughing (see *Annotations of Perceived Sounds* section for details). Natural sounds include birds, seagulls, rain, and dogs. Technological sounds include traffic, various objects, and noises. Figure 10 places the diary notes on a timeline, by phases. Illustrations visually represent the keywords associated with the corresponding categories in the diary notes, telling the story of each category’s change over time. In particular, the category “human” is characterized in the diary notes with keywords associated with the outdoor presence of people at the beginning of lockdown (Phase 1), while during the more restrictive Phase 2, human sounds are described as coming from indoor through open windows. In Phase 1 and Phase 2 notes, such keywords appear as little as five times and one time, respectively. On the other hand, during the most restrictive Phase 2, Natural sounds (birds, but also meteorological elements such as wind, rain, and thunderstorms) appear 11 times, emerging as the most prominent taxonomic category. As shown in Figure 10, in Phase 2, we can also observe a temporary decrease in Loudness, which, as illustrated in *Results* section, might be interpreted as related to the concurrent decrease in human activity. Perceptual indicators for Eventfulness also sharply decrease during Phase 2, while indicators of Pleasantness do significantly increase. Likewise, Natural sound sources, mainly related to birds, seem to be more dense in the phases leading up to Phase 3. With the progression of the “Plan de Desescalada,” Human sources are described as coming more and more from the street as their frequency increases, with human-related keywords appearing 14 times in the diary notes of Phase 3. A slight, temporary increase in technological sounds during the initial phase of confinement seems to be reflected in the diary notes, with related keywords appearing as much as four times compared with only two mentions of natural sounds. Finally, changes in the diary notes over the whole lockdown period seem to be reflected in the results of qualitative analysis. The Loess curve associated with Human sounds shows a slight decrease throughout Phase 1 and the following Phase 2, while it increases steadily in Phase 3. The increase in Human sounds is mirrored by a sharp increase in perceptual indicators of Eventfulness in Phase 3, while Pleasantness clearly decreases. Overall Loudness increases during the entire period.

## Discussion

The increase in Human-generated sounds appears to be the reason for a perceived increase in Eventfulness within the soundscape of the area and a higher degree of Loudness in Phase 3. In the initial Phase 1, leaving one’s house was allowed only for work-related tasks and essential shopping. This represented a great change in the habits of local residents. As described in *Site of the Case Study* section, locals gather around the plaza in the hours before lunch (between 12:00 and 14:00, the time of the recording). Adults occupy local cafes for drinks, while kids play at the playground (during non-school days). During Phase 2, these habits were forcibly suspended, a fact that might explain the generally higher level of Pleasantness, which increased in this phase, and the generally lower level of Eventfulness, which also increased. Diary notes of Phase 1 capture the presence of human voices as an indicator of “too many people” being “still around,” “enjoying a chat,” and “resonating in the little plaza.” Human voices, perhaps amplified by the architectural features of the location (the arcade, see *Site of the Case Study* section), seem to be the most relevant image in the soundscape. In Phase 2, with additional restrictions to activity, humans seem to finally leave the scene in favor of birds and other animals (mainly dogs), which become prominent in the Diary Notes. Diary notes (“Day 7. Of voices populating windows” and “Day 8. Stay inside but keep your window wide open, especially if it’s Sunday”) remind us that neighbors tended to leave their windows open more often. Voices and human sounds were recorded as emanating from the inside of other apartments, revealing to the author the intimacy of family life (“Day 19. In the silence, someone’s getting ready for lunch”). It is worth noticing that during Phases 1 and 2 (mid-March to mid-April), temperatures in the area were warmer than the average (Agencia Estatal de Meteorología [AEMET], 2020). This might have contributed to residents moving part of their daily activities out onto their balconies and keeping windows open more often, which caused activities to be heard. As illustrated in *Site of the Case Study* section, the habit of sharing indoor life with the world outside is not common in this area of Spain. Diary notes confirm how exceptional such a behavior was: “Day 22. A good thing of these days is that I am finally seeing my neighbors, from window to window. Nobody used to lean out of the window in this *barrio* [neighborhood] of mine, before” and “Day 65. Last night we had the last collective clapping for healthcare workers. What will happen to my neighbors now?” An increased sense of community during and after lockdown has been reported by several sources. One study found that “a substantial proportion of people felt that they had become more involved in neighborhood life following the lockdown” (Jones et al., 2020) and that support among neighbors include “raising morale through humor, creativity and acts of kindness and solidarity” (idem). Another observer wrote that “It’s not just the help and practicalities, the socializing too is vital. Conversations across balconies, news being discussed and sometimes neighbors humming along to music being played next door” (Banerjee, 2020). This apparently new attitude might be connected to a psychological reaction to isolation during lockdown (Henley, 2020) and the need to share with neighbors during such a unique time. The limitations of the present study do not allow for a conclusive interpretation of results. It is difficult to say whether the local residents of the road kept their windows open due to the exceptional meteorological conditions or as the result of psychological reaction to isolation. Note that both Diary Notes and Annotations by the expert group indicate the influence of the indoor soundscape on the outdoor soundscape, which might be due to a reduction of other common sources of outdoor sounds.

In fact, machinery and human interaction sounds decreased during the lockdown. This result needs to be considered within the context of the specific location under study, a pedestrian road where traffic noise even during normal times is within regulatory levels. Restrictions such as Phase 2 total ban of any non-necessary activity and a stop on constructions and home renovations should be taken into account. It is only in the last phase of the “Plan de Desescalada,” in mid-June, that this ban was lifted. It is well known by now (Garcia, 2020) that such restrictions on human activity had a strong impact on the local mobility of people and vehicles. In the whole Basque Country, public transport was reduced by 50% during Phase 1 and only recovered full capacity with the entrance in the so-called “new normality” in late June. Conversely, traffic of light vehicles in the Basque Country decreased by 95% (Dirección General de Tráfico, 2020) during Phases 1 and 2, along with traffic of heavy vehicles, which is a well-known source of noise pollution (Jacyna et al., 2017;, Kulauzović et al., 2020), decreased by more than 50% (Ortega Dolz, 2020). With the ban on mobility and closure of international borders, the local airport was exceptionally quiet, and the reduction in international air traffic was 95% (Alonso, 2020). By contrast, maritime activity at the port of Bilbao fell by only 5% (Alvarez, 2020). The disparity in reduction between these two types of trade and travel (air or sea) reflects not only their influence on the economy but also their very different levels of impact on the acoustic environment. In fact, if the maritime traffic does not affect the acoustic environment of Las Arenas, traffic from the airport can be heard at times over the area, mostly when, due to specific meteorological conditions, aircrafts land from the sea, thus flying over Calle Paulino Mendibil. Interestingly, the acoustic presence of aircrafts is also reflected in the diary notes, on Day 6: “Day 6 of lockdown from my window in Getxo, Basque Country. It sounds someone [*sic*] is still flying out from here. Or in, who knows.”

Looking at our results, the perceptual analysis seems consistent with an urban soundscape where acoustic events are more rarefied during Phases 1 and 2 of the lockdown, while Eventfulness grows in Phase 3, when mobility for leisure is allowed and public as well as private transport is progressively restored. As for human interaction, two factors are worth noticing: the influence of the reopening of cafes and restaurants, with outdoor spaces on the public pedestrian road being allowed extra hours in order to make up for the economic loss of the lockdown weeks; and the reopening of the children playground in the square located opposite the observation point, in a moment (Phase 3) where schools were closed, due to the end of the academic year, which coincided with the end of the “Desescalada.” Toward the end of Phase 3, the increase in Eventfulness is testimony to the progressive return of local habits and behaviors, described in *Site of the Case Study* section.

As for the category of Natural sounds, undoubtedly, the sonic imagery of birds singing and birdsong has grown in importance during lockdown. Media have widely reported an increase in attention toward the singing of birds by both the scientific and artistic communities. From the launch of the first international global soundscape of spring dawn chorus created by artists and scientists (Morss, 2020), to birdsong becoming “more beautiful” (Cockburn, 2020) or “sexier” (Chrobak, 2020) thanks to the absence of human activity, birds seem to have grown to represent the essence of the urban soundscape in lockdown to the point that they could condition political choices (The Economist, 2020). The results of the present studies seem to support these claims at least for Phase 2, when the most restrictive measures were applied to human mobility. In this phase, an increase in the presence of natural sound sources (mainly birds) is observable in the annotations as well as in diary notes, where the word “birds” is often noted down in isolation, as if it could contain by itself the whole imagery of a pleasant soundscape. In our study, Pleasantness increased during Phase 2, while Eventfulness decreased, allowing for relating the sonic image of birdsong with that of pleasantness and calm.

A separate reflection should be dedicated to the consequences of lockdown regulations on light mobility. Findings seem to indicate that a growth in perceived sounds of Wheels, a.k.a. manual (non-electric) PMVs, contribute to a perceived increase in Eventfulness, an increase in Loudness, and decrease of Pleasantness of the soundscape in Phase 3. Rather unexpectedly, scooters and other light vehicles seemed to produce a considerable perceptual annoyance, at least when used on pedestrian non-PMV-specific surfaces and nearby residential dwellings, such as the observation point of this study. During the same time period as the present study, according to a study by the Spanish insurance company Acierto and widely circulated by the media (Gutierrez, 2020), the use of bicycles grew by as much as seven times. Purchases have increased by 30% since Phase 1 (Blanchar, 2020). Usage of manual and electric scooters as well as other light mobility has also grown. Additionally, from Phase 3 onward, children were allowed out and expressly permitted, if not encouraged (Lucas, 2020), to use PMVs such as skateboards, roller skaters, and manual scooters. At the time, extended media coverage was dedicated to the claim that “the pandemic and this crisis is causing a rethinking of many issues in life. It will give much more voice to people who do not use but who suffer from the presence of the car. This element, essential in our culture, will no longer be the privileged element of the city. The post-COVID city will be the post-car city” (Spanish urban planner Jose Ezquiaga interviewed by Mendoza Pérez, 2020). In our analysis, we highlighted how evidence could be found that in Phase 3 there was an increase of perceived sounds of Wheels (light vehicles) vs. perceived sounds from Vehicles (traditional fuel vehicles, cf. Figure 8, third row, right). It is difficult to say whether these changes will be permanent, or whether a return toward private (mainly fuel-based) transport is to be expected, given the risks associated with traveling on public transport while the pandemic is still unresolved. In the months following lockdown, the motorcycle sector in Spain “witnessed a growth above double-digit, still among great economic uncertainty” (Asociación Nacional de Empresas del Sector de Dos Ruedas [ANESDOR], 2020). We can posit though that the environmental presence of light mobility is important and that specific lane/pavements might be considered to be included as urban design criteria. It is indeed expected that in future cities, the traditional prominence of fuel cars will be superseded by a range of other kinds of mobility devices. “The world of vehicles is exploding in thousands of shapes and sizes. We are witnessing our cities being more and more conquered by vehicles of different kinds, dimensions and number of occupants” (Sádaba, 2019). Fumihiko Maki talks about the intangible “linkage” as the glue, “the act by which we unite the different layers of activity and resulting form of the city” (Maki, 1964). Changes in mobility, means of communication, and social interaction will probably soon redefine the eventual shape of cities, a shift that the COVID-19 pandemic seems to have accelerated. On the one hand, the increase in light mobility will have an impact on the width and layout of traffic lanes and on the design and occupation and management of sidewalk curbs (Goffman, 2018). On the other hand, smart working and a consequent decrease in face-to-face meetings might reduce the need for human displacement within cities. Koolhaas’ “event structure” of cities (Kipnis, 1996) will become diverse and multifaceted. The limitations of the present case study do not allow us to generalize results in order to imagine potential scenarios of changes in the urban soundscape in the case of a decrease in fuel cars. Nonetheless, we believe that by measuring and analyzing *intangible* aspects of the city, such as the acoustic environment, we can gather precious insights to optimize the design of appropriate indicators for future quality of life, as well as health, and a better management of finite resources. In our study, we combined what Gehl and Svarre call “Keeping a Diary,” “Photographing,” and “Tracking” (our soundscape recordings) with quantitative and qualitative analyses, to understand the social behavioral changes triggered by the lockdown. Soundscape research provides crucial knowledge, allowing a better understanding of city life. This study contributes to opening up further research on the tangible–intangible duality in order to offer improved urban indicators for city and mobility design.

## Conclusion

When the COVID-19 crisis subsides, we might be able to compare and synthesize results from these varied endeavors and many more. It is yet too early to speculate about what might be learned from the experiences we – all of us – are currently making in exceptional times. Soundscape research provides vital clues to understanding the perception and design of the multimodal environment – how humans are psychologically, physiologically, physically, and socially affected by sound; and also, how other living creatures are likewise affected. It makes a constructive and oftentimes undervalued contribution. Will researchers, sound designers, and architects be part of a discussion with urbanists, policy makers, politicians, businesses, and indeed the general public, in seeking solutions to the mounting challenges to urban living conditions in the future?

We believe that the tangible–intangible duality can be applied as a holistic approach to include both objective and subjective indicators to the evaluation of urban soundscape. At the start of this article, we briefly recalled the origins of the soundscape movement and its influence on urban ecology and urban planning. Still, public endeavors such as the Noise White Paper of Getxo exemplify the fact that “landscape architecture and related disciplines have not fully recognized the possibilities of considering sound issues in design projects” (Cerwén, 2017). The Noise White Paper of Getxo is written in development of the Acoustic Pollution Decree of the Basque Government (Decreto 213/2012, de 16 de octubre, de contaminación acústica de la Comunidad Autónoma del País Vasco) that, undeniably, only focuses on the negative aspects of sound and/or noise in order to detect problematic points in the urban landscape and mitigate the effects and the subsequent discomfort they may cause. Furthermore, the project for the Law for the Protection of Landscape of the Basque Government (1998) does not make any specific mention of soundscape as one of the elements of urban planning. This is all the more remarkable given that for more than two decades the European Commission has had policies in place aimed at reducing noise exposure. Indeed, noise is still “the ignored pollutant” (King and Murphy, 2016), but one way forward is to focus less on noise in general and more on how to promote specific and positively valenced sounds in the environment (e.g., Davies et al., 2013; Aletta et al., 2016a). Our study aims to contribute to an understanding of the relationship between sound sources and holistic soundscape evaluation. Through this study, we employed a mixed methodology that aims to measure both tangible (such as loudness) and intangible (such as perception of quality) aspects of the environment of a specific neighborhood of the city of Getxo. We believe that it would be possible – and advisable – to introduce this approach to the analysis of the urban soundscape with the ultimate goal to include the attention to the audible landscape of the city in the procedures established by the law in terms of landscape protection and ultimately in the local urban planning policies.

Specifically, we seek to continue the development of such a mixed methodology in terms of both qualitative and quantitative research. On the one hand, we will further develop the collection of field notes and direct observations of the soundscape as a complement to the collection of field recordings. In circumstances other than those allowed by the restrictions imposed to mobility during lockdown (that affected the development of this study), we recommend that such field notes and observations are complemented by interviews to residents and other participatory activities.

## Data Availability Statement

The raw data supporting the conclusions of this article will be made available by the authors, without undue reservation.

## Ethics Statement

The collection of diary notes and soundscape recordings did not require an ethics approval from the institution of the first author. The procedures for data colloction for soundscape evaluation and annotation were approved by the Research Ethics Committee of City University of Hong Kong (ref. 13-2020-08-E). The expert group members provided their written informed consent to participate.

## Author Contributions

SL and JS conceived the study. SL collected audio, photos, and diary notes. PL conducted annotations and evaluations by the expert group and analyzed the data. SL, JS, and PL wrote the manuscript. JS made illustrations and drawings. All authors contributed to the article and approved the submitted version.

## Conflict of Interest

The authors declare that the research was conducted in the absence of any commercial or financial relationships that could be construed as a potential conflict of interest.

## Footnotes

1 https://www.google.com/maps/@43.3257696,-3.014469,169m/data=!3m1!1e3

## Supplementary Material

Supplementary Data Sheet 1 Audio Selection.

Supplementary Data Sheet 2 Diary notes.

Supplementary Data Sheet 3 Phases of the lockdown regulations.

Supplementary Data Sheet 4 Instructions to the expert group for soundscape annotations.

Supplementary Data Sheet 5 Pleasantness-Eventfulness plots for all the days in the study.

Supplementary Data Sheet 6 Instructions to the expert group for soundscape evaluations.

Supplementary Data Sheet 7 Annotations and levels of the sound source taxonomy (in pdf format).

Supplementary Data Sheet 8 Annotations and levels of the sound source taxonomy (in csv format).
