# Supplementary material for: Soundscape in Times of Change: Case Study of a City Neighbourhood During the COVID-19 Lockdown
Source: Front Psychol. 2021 Mar 24;12:570741. doi: 10.3389/fpsyg.2021.570741 (PMC8024535; doi:10.3389/fpsyg.2021.570741)
Supplement: Supplementary Data Sheet 2 — Diary notes. [file Data_Sheet_2.PDF]

Diary notes

Day 1	With a good part of humanity in lockdown due to COVID-19 epidemics, our cities have gone quieter. I'm recording a soundscape a day, everyday at the same time, same place as a sonic postcard to the future from these strange days.
Day 2	Sounds like too many people are still around.
Day 3	Noon, in front of my house. Constructions (?), distant music…life that a picture does not show.
Day 4	I never noticed how much human voice resonated in the little plaza in front of our window. Interesting how the still image always look the same, day after day, while soundscape is so varied.
Day 5	It's a public holiday today in Spain, Father's day, San José. And strangely enough, something in the air makes us feel it's a holiday even if nothing really changes holidays or not, these days.
Day 6	Day 6 of lockdown from my window in Getxo, Basque Country. It sounds someone is still flying out from here. Or in, who knows.
Day 7	Of voices populating windows. A good thing of these days is that I am finally seeing my neighbours, from window to window. Nobody used to lean out of the window in this barrio of mine, before.
Day 8	Stay inside but keep your window wide open, especially if it's Sunday.
Day 9	Things are getting quieter, dogs are getting braver, birds might follow.

Day 10 Life goes on, birds get louder, and the church bells disappeared.

Day 11 A sonic postcard from this brave new world.

Day 12 The days of an extended family in lockdown are paying its toll but…here we are, Day 12: humans still enjoy a chat, especially when the sun shines outside.

Day 13 Colder, quieter, almost the week-end.

Day 14 Near silence.

Day 15 12pm, Sunday, first day of Spring time. They say it’s the last year we change the time.

Day 16 Making noise is feeling alive.

Day 17 New restrictions kicked in.

Day 18 Of seagulls and wheels.

Day 19 In the silence, someone’s getting ready for lunch.

Day 20 It’s the eve of the Semana Santa in Spain. Somehow, in the silence, you can tell a festive season is coming.

Day 21 Semana Santa-ish (are those church bells that I hear in the far distance?)

Day 22 They told us we’re staying in twenty more days. And that after that, more days will come.

Day 23 Raindrops on the mike.

Day 24 Erase una vez, un lobito bueno. ["Once upon a time, there was a good little wolf."] - put in a footnote: it's the song played with the harmonica you can hear in the rec :)

Day 25 Confinement is starting taking its toll. Not to the world outside, but to the world inside. Today I managed to record 30 seconds before the family exploded. Will do better tomorrow.

Day 26 You can always count on toddlers.

Day 27 Rarefaction.

Day 28 Birds, birds, birds.

Day 29 Easter. And birds.

Day 30 Day 30. Or in other words, a month of lockdown/locked in.

Day 31 I went on strike with myself and did not record.

Day 32 Wind is back.

Day 33 I start needing a change. A baby girl on the outside/inside border.

Day 34 It’s somebody’s birthday in the air.

Day 35 They say there’s probability of thunderstorms. Birds already know it.

Day 36

Day 37 You cannot hear the queue for the bank. Meanwhile, animals are taking over.

Day 38 The rain erased (nearly) all human sounds. But not birds.

Day 39 Starting Sunday children will be allowed out. Is it excitement I am hearing in the air?

Day 40

Day 41

Day 42

Day 43 They said children can go out for one hour a day, so the little one and I went to see the sea, after 43 days.

Day 44

Day 45

Day 46 The wind is back as usual here, but this time, children are back, too.

Day 47 The wind is gone, children are here to stay. And birds.

Day 48 It’s Labour Day, little by little, people are taking back the streets.

Day 49 Starting today, we are allowed to take the road for sport.

Day 50

Day 51

Day 52 The world seems to be back! And the church bells, timidly, too.

Day 53

Day 54

Day 55

Day 56

Day 57 It’s getting tough (for parents, workers, and those who are both things!) and we really don’t see the end, but children and birds don’t know and don’t care. And it's great.

Day 58

Day 59

Day 60 After two months of (almost) daily recording the soundscape of the little plaza in front of my window, as the Basque Country rolls out what in Spain is called “Phase 1”, with small retailers, hairdressers, hospitality open and people happily and maybe unwisely taking the road, I decided to stop publishing - even though I’ll keep recording as we move towards “the new normality”. Good luck everybody, who knows what a brave new world is awaiting us out there!

Day 61

Day 62

Day 63

Day 64 Kids do not know social distancing, neither their parents.

Day 65 Last night we hade the last collective clapping for healthcare workers. What will happen to my neighbours now?

Day 66 They say from Monday masks will me mandatory, voices will be muffled.

Day 67

Day 68

Day 69 Quietly, trees are growing in front of the window.

Day 70 The rain won't stop our race to Phase 2.

Day 71

Day 72 Cosmo opened today, the world seems back to order, even birds sound happy.
