# Supplementary material for: Soundscape in Times of Change: Case Study of a City Neighbourhood During the COVID-19 Lockdown
Source: Front Psychol. 2021 Mar 24;12:570741. doi: 10.3389/fpsyg.2021.570741 (PMC8024535; doi:10.3389/fpsyg.2021.570741)
Supplement: Supplementary Data Sheet 3 — Phases of the lockdown regulations. [file Data_Sheet_3.PDF]

Phase -3	Phase -2	Phase -1
30-03 to 09-04	15-03 to 29-03;<br>10-04 to 22-04	23-04 to 01-05
**Mobility of people**	**Mobility of people**	**Mobility of people**
• Food and medicine shopping<br>• Emergencies<br>• International and inter-regional borders are closed	• Same as Phase -3<br>• Job - related activity	• Same as Phase -2
**Mobility of vehicles**	**Mobility of vehicles**	**Mobility of vehicles**
• Public transport is reduced by 50.<br>• Leisure driving is forbidden<br>• Goods delivery is permitted	• Same as Phase -3	• Same as Phase -3
**Outdoor activity**	**Outdoor activity**	**Outdoor activity**
• Not permitted	• Same as Phase -3	• Children can go out for 1 hour, once a day
**Social contact**	**Social contact**	**Social contact**
• Not permitted: people allowed out have to be alone	• Same as Phase -3	• Children (up to three siblings) can be outdoor with one adult with whom they live
**Retail**	**Retail**	**Retail**
• Food stores and pharmacies are open<br>• Everything else is closed	• Same as Phase -3	• Same as Phase -3
**Cultural and social activity**	**Cultural and social activity**	**Cultural and social activity**
• Not permitted	• Same as Phase -3	• Same as Phase -3
**Education**	**Education**	**Education**
• Schools of all order, from nursery to university, are closed	• Same as Phase -3	• Same as Phase -3
**Hospitality**	**Hospitality**	**Hospitality**
• Bars and cafés, restaurants and hotels are closed	• Same as Phase -3	• Same as Phase -3

Phase 0	Phase 1	Phase 2
30-05 to 10-05	11-05 to 24-05	25-05 to 07-06
**Mobility of people**	**Mobility of people**	**Mobility of people**
• Same as Phase -2<br>• Permitted outdoor activities are limited to 1km radium from residential address	• Same as Phase 0	• Same as Phase 0<br>• Permitted outdoor activities are limited to the province of residency
**Mobility of vehicles**	**Mobility of vehicles**	**Mobility of vehicles**
• Same as Phase -3<br>• Public transport progressively back to 100% capacity	• Same as Phase 0<br>• Leisure driving is allowed to reach outdoor activities locations or second residency, within the province	• Same as Phase 0<br>• Leisure driving is allowed including for camping
**Outdoor activity**	**Outdoor activity**	**Outdoor activity**
Permitted:<br>• 6AM to 10AM and 8PM to 11PM, individual sport, strolls in the company of others with whom you share domicile (no kids)<br>• 10AM to 12PM and 7PM to 8PM over 65 and other categories at risk<br>• 12PM to 7PM, children with one parent	• Same as Phase 0	• Time slots restrictions are lifted
**Social contact**	**Social contact**	**Social contact**
• Outdoor activities can be performed with people you live with	• Up to 10 people, with social distancing measures	• Up to 15 people, with social distancing measures
**Retail**	**Retail**	**Retail**
• Retail under 400sqm can open, capacity limited to 30%	• Retail up to 400sqm can open, capacity limited to 30%	• Retail above 400sqm can open, capacity is limited to 30%.
**Cultural and social activity**	**Cultural and social activity**	**Cultural and social activity**
• Religious temples can open at 30% capacity	• Same as Phase 0	• Same as Phase 0<br>• Gyms can open with distancing measures and for individual sport, on appointment<br>• Museums, cinemas and theatres can open with capacity 30%

Education	Education	Education
• Same as Phase -3	• Same as Phase -3	• Opened for students between 15 to 19 years old
**Hospitality**	**Hospitality**	**Hospitality**
• Bar, restaurants, cafés can open for take-away only	• Bar, restaurants, cafés can open, only outdoor premises, with 30% capacity<br>• Hotels, holidays apartments can open with 100% capacity but common areas remain closed	• Bar, restaurants, cafés can open also indoor premises, with 50% capacity<br>• Hotels, holidays apartments can open common areas, with capacity limited to 30%
