# Supplementary material for: Soundscape in Times of Change: Case Study of a City Neighbourhood During the COVID-19 Lockdown
Source: Front Psychol. 2021 Mar 24;12:570741. doi: 10.3389/fpsyg.2021.570741 (PMC8024535; doi:10.3389/fpsyg.2021.570741)
Supplement: Supplementary Data Sheet 4 — Instructions to the expert group for soundscape annotations. [file Data_Sheet_4.PDF]

[Link to this document: https://bit.ly/2ZAHLb3.]

How to annotate the soundscape recordings

Naming conventions

Annotators are given a code-name. If you are Annotator “D”, then the sounds you are working with are named “D-01.mp3”, “D-02.mp3”, “D-03.mp3” etc. The annotation files that you will be sending back to us have to be named accordingly, and exactly: “D-01.txt”, “D-02.txt”, “D-03.txt” etc. (So if you are Annotator J, your sounds are named “J-01.mp3” etc, and the files you send back are named “J-01.txt” etc.)

Your task

You are given a set of 50 soundscape recordings. They are taken at the same location but at different points in time. They are presented randomly so you will not know the order in which they were made.

You will be using the “label” tool in Audacity, described in detail below. Listening to each recording, identify as many sound sources as you can – but don’t overwork it – around 8 to 12 annotations for a 2-minute soundscape might be appropriate (see example in Fig. 8). Your tags could be things like “voices”, “church bells”, “car alarm”, “child laughing”, “birdsong”, “footsteps”, “car engine”, and so forth. Use your own words and categories; be concise.

Once you get familiar with the procedure, it should take around 5 minutes to annotate a 2-minute recording. In two hours, you would complete between 20 and 30 soundscapes. Remember that you are not obliged to finish all the soundscapes given to you. After learning the software, we hope that you are able to put in two hours to the task. Each file that you annotate is valuable to our research. We are very grateful for your contribution.

Doing annotations

To do good-quality annotations, you need:

- A quiet, comfortable environment and a computer;
- Good headphones (preferably studio-quality covering the ears fully; avoid earbuds);
- Set the headphones volume at a fixed, comfortable level (don’t change it underways);
- Calm of mind (don’t stress, don’t work for too long; take mini-breaks often).

Audacity

Audacity is a free and open source software. *The best. Ever.* Download and install it by following instructions here: https://www.audacityteam.org/

It is available for Mac, Windows, and Linux platforms. In what follows all screenshots and keyboard shortcuts are for Mac. If you use another system and encounter problems you cannot solve, please contact PerMagnus at pm@permagnus.org.

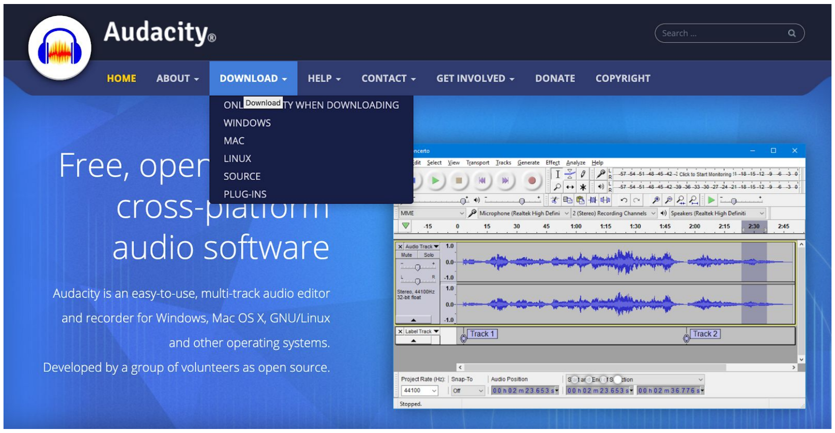

Figure 1.

(Pro tip: If you use Audacity under Mac OS 10.15 (Capitan) there is a known issue regarding audio recordings. There is a workaround which you can find via the Audacity Forum. You don't need to make any recordings right now.)

Audacity settings

Open Audacity and its Preferences, either from the File menu as in Figure 1, or by typing Command-comma (the standard Mac keyboard shortcut for preferences).

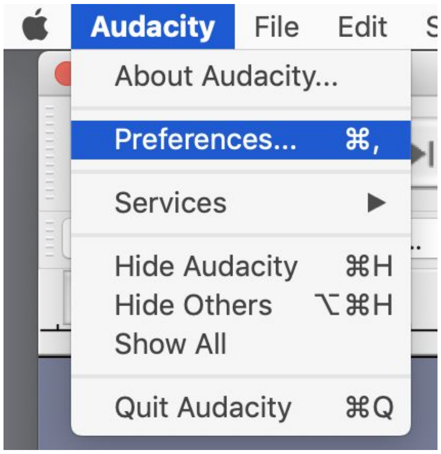

Figure 2.

In the Preferences floating window, click on Tracks >> Spectrograms. Enter all the selections as shown in Figure 3. You only need to make these preference settings once, and all your spectrograms will look good.

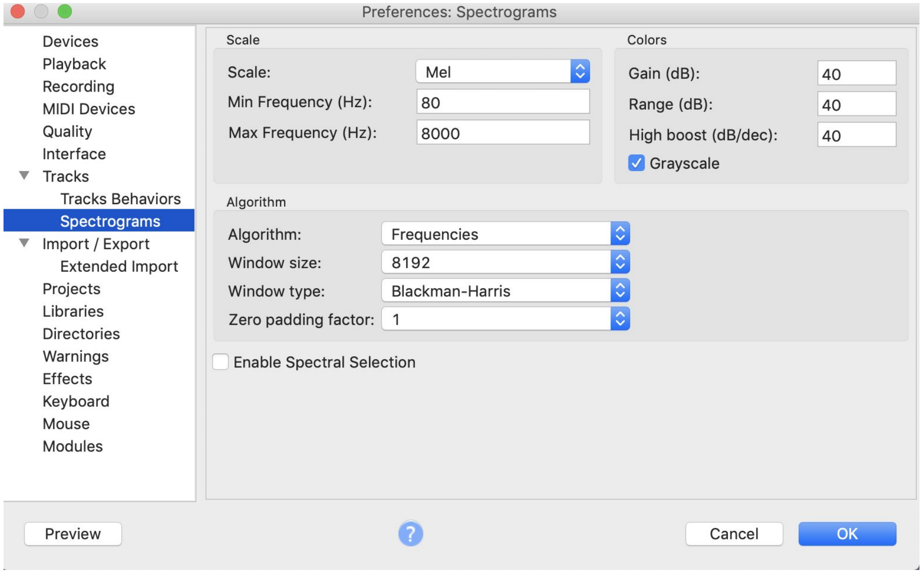

Figure 3. Note that the box "Enable Spectral Selection" should be un-ticked.

(Strictly speaking it is not essential to use the spectrogram settings above, but through experience I have found them to be optimal for this kind of task.)

Files and folders

To work smoothly with the annotations, keep all files and folders organised. You will have received a link to download material for you. Expand the folder and it looks something like Figure 4.

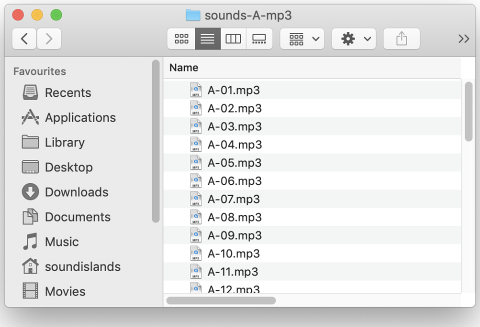

Figure 4. Screenshot of the folder with sounds for Annotator A (example).

Listening and viewing sounds

Open the files in Audacity in numerical order.

1. Maximise the window by clicking on the 'green button' on the left and upper corner (optional);
2. Select the whole file (Command-A); see Figure 5a;
3. Fit width (Command-F), and height (Command-Shift-F); see Figure 5b;

4. Click the track name on the left side (small triangle pointing downwards), and select Spectrogram view, Figure 5c;
5. Adjust to view one channel only by pulling at the dividing line (optional).

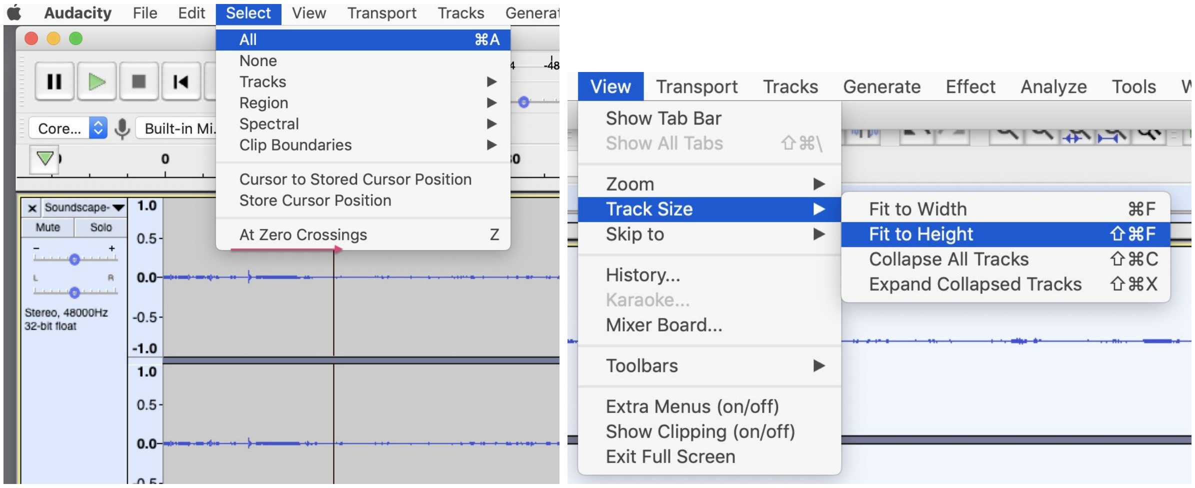

Figure 5a and b.

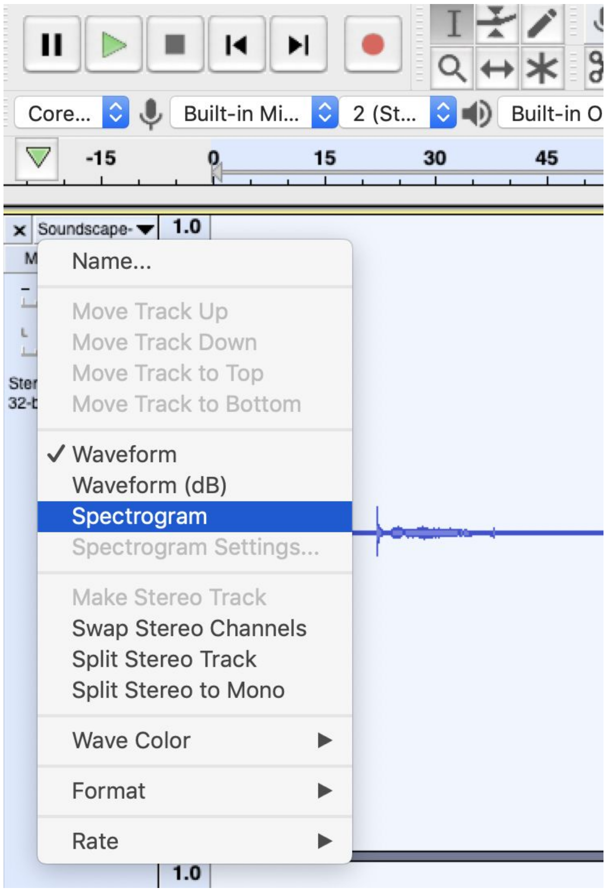

Figure 5c. Choose Spectrogram view.

Having made these Preference settings, you are able to view the audio file as a spectrogram in the optimal way. It should look a bit like in Figure 6.

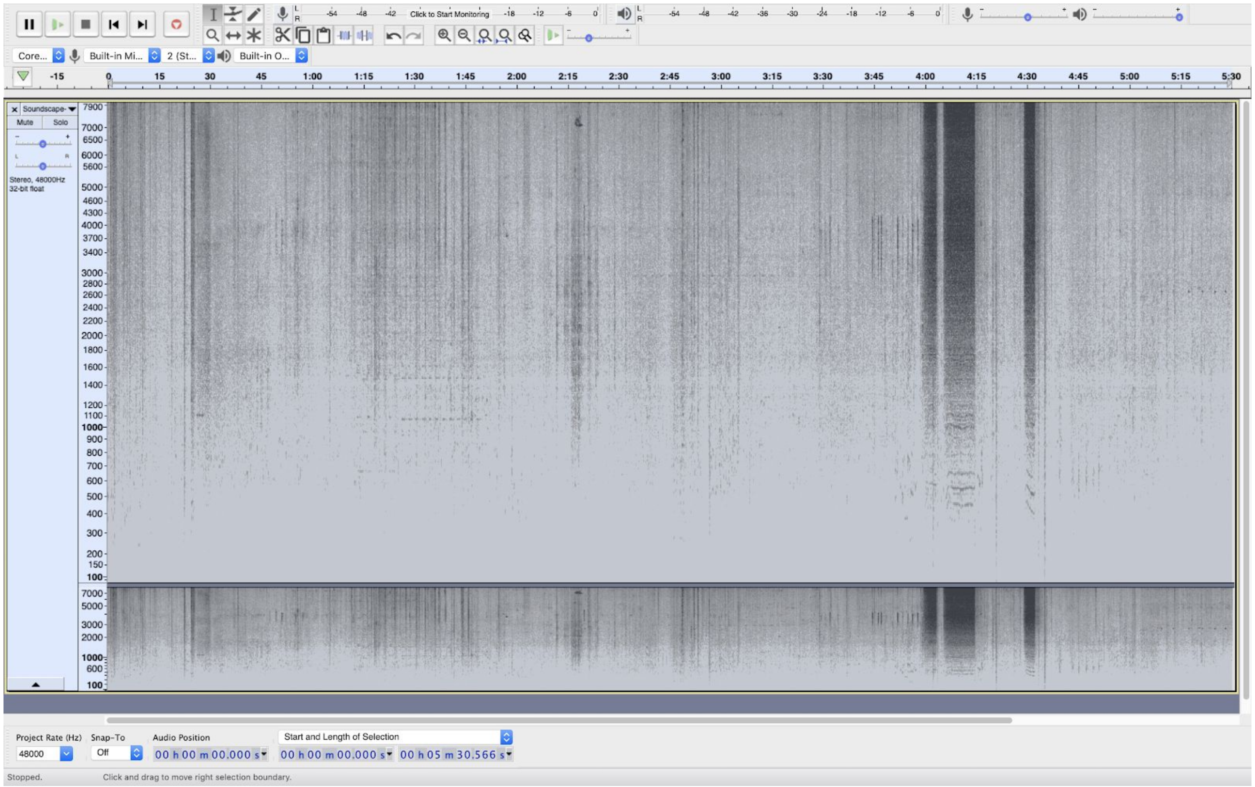

Figure 6.

(Pro tip: Notice that in Figure 6 one stereo channel takes a lot of screen space, and the other channel (sitting below) much less. You can adjust the view to your own taste by pulling the dividing-line, upwards or downwards.)

Play around and listen

Press the space-bar to start playing (or click on the "play" triangle). To start somewhere else in the file, click on the time-ruler above the spectrogram: the play-cursor jumps to and continues playing. When you press space bar again, playing stops. Notice that the spectrogram shows audio frequencies from low to high, and that 'more black' means louder. You will soon get used to 'seeing what you are hearing' – it is a powerful tool.

Workflow tips

Here's recommendations from Juan (beta tester ;) describing a good way to work on the files:

1. *Listen first to the feel without labelling.*
2. *Label approximately in place the sounds that you heard, in a second listening or by heart/hand drawn annotations.*
3. *Adjust the labels on a second/third listening to their appropriate spots. Remember that if you click on the sliders of the labels you move the full label.*

Annotations ==> Labels

Labels and annotations are the same thing. When you hear (and see in the spectrogram) a sound source, make a selection by click-dragging across the time period during which it is active and you can identify it. To create the Label, press Command-B, and the Label will appear on the Label Track, for the selection that you've made. Type a word or two to describe the sound source. The result might look like in Figure 7.

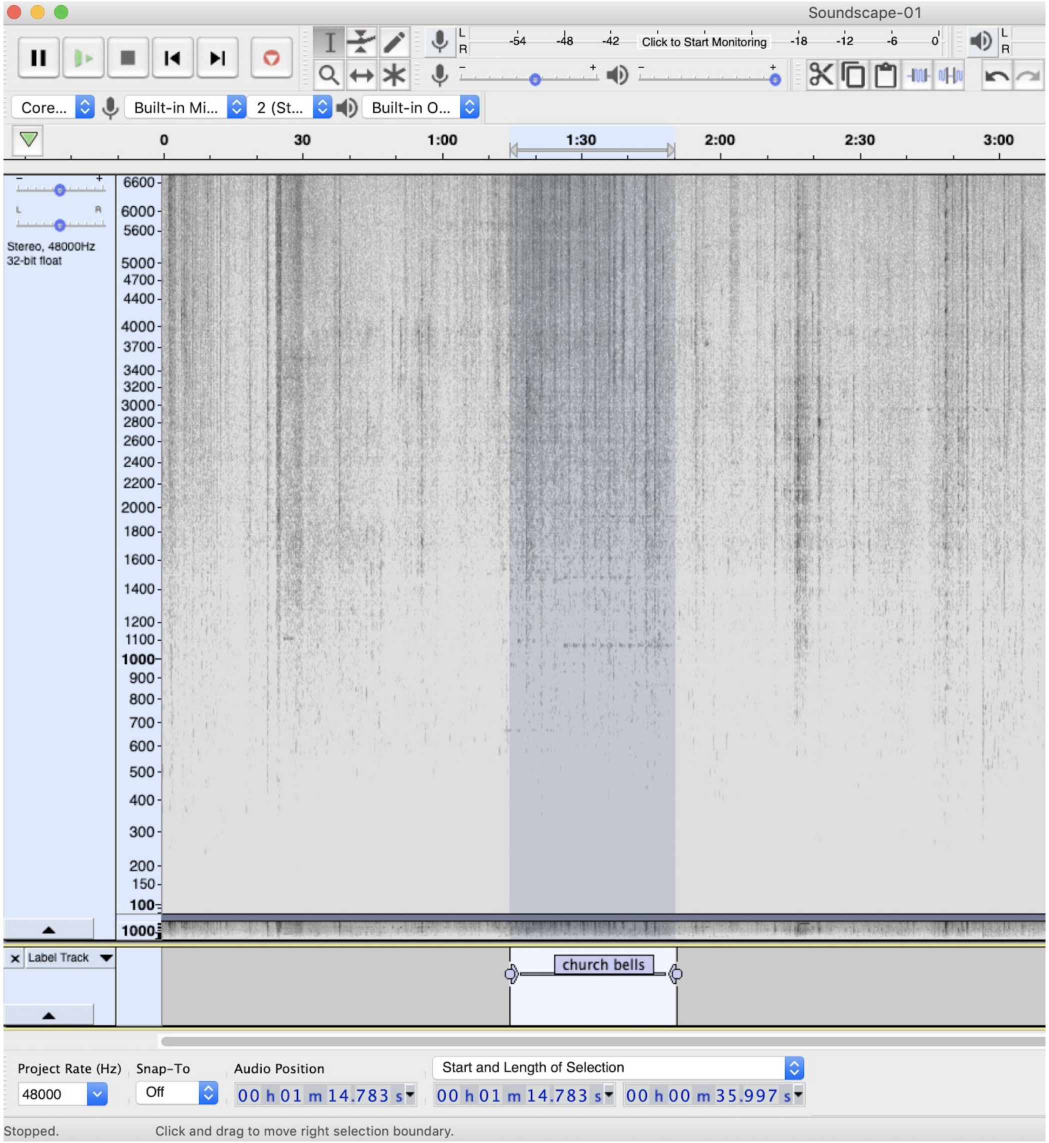

Figure 7. The Label Track with one named label (example).

Pro tips:

- You can adjust the start and end points by dragging the little half-moon shapes – this will make the selection smaller or bigger.
- You can also move the whole selection by dragging either of the little circles.
- You can rename the Label by double-clicking in the text box.
- To delete a Label is a little tricky! You have to open Edit >> Labels, and get the floating window where you can select one of the labels and click 'delete' at the right side. See Figure 8a and b.

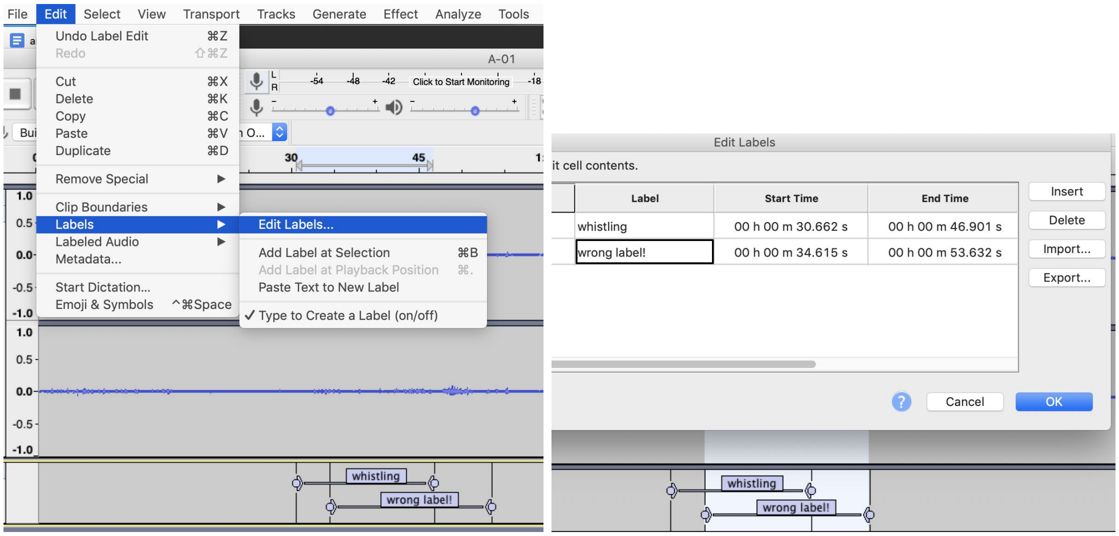

Figure 8a and b. How to delete a label.

More tips and recommendations

- Note that you <u>cannot delete labels with command-X</u>, because this cuts the selection from the sound/spectrogram too!
- *The Label <u>duration for a sustained or repeated sound should be long</u>, marking the whole duration that you can hear the sound (even if it is not necessarily loud). This is often the case for birdsong or people speaking far away.*
- *The Label duration for a brief (and maybe loud) sound, like something being struck or dropped to the ground, should be short.*
- A 'finished' annotation (of a 2-minute recording) might look like Figure 8, but remember that it depends on the soundscape as well as the way you are listening.

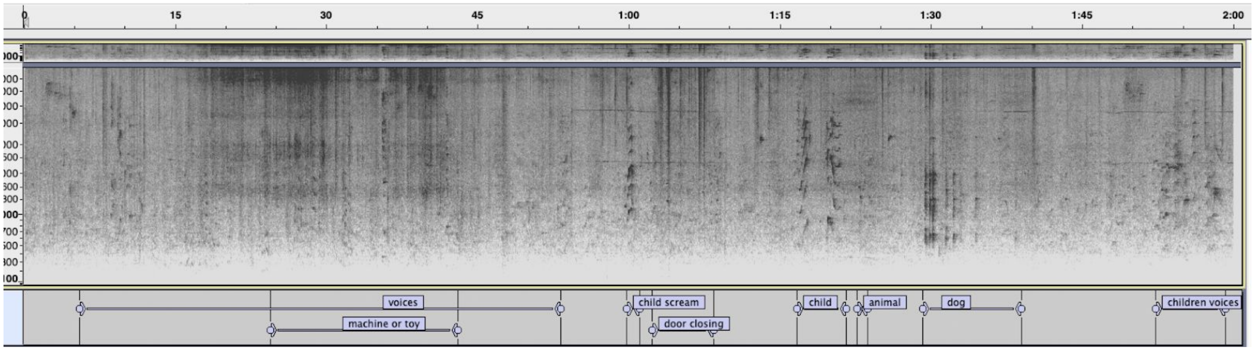

Figure 8. Two-minute soundscape with 12 labels.

Saving your annotations

When finished (don't work on a 2-minute recording more than 6 minutes), save the data file:

1. Go to the Menu bar: File >> Export >> Labels, as in Figure 9a.
2. In the dialogue window, name the file you're exporting exactly like the file you're annotating, strictly following your *'Annotator name'* (e.g. "A-01.txt" if you are annotator A and exporting labels for sound 01). Save it in the subfolder with your code-name; as in Figure 9b.

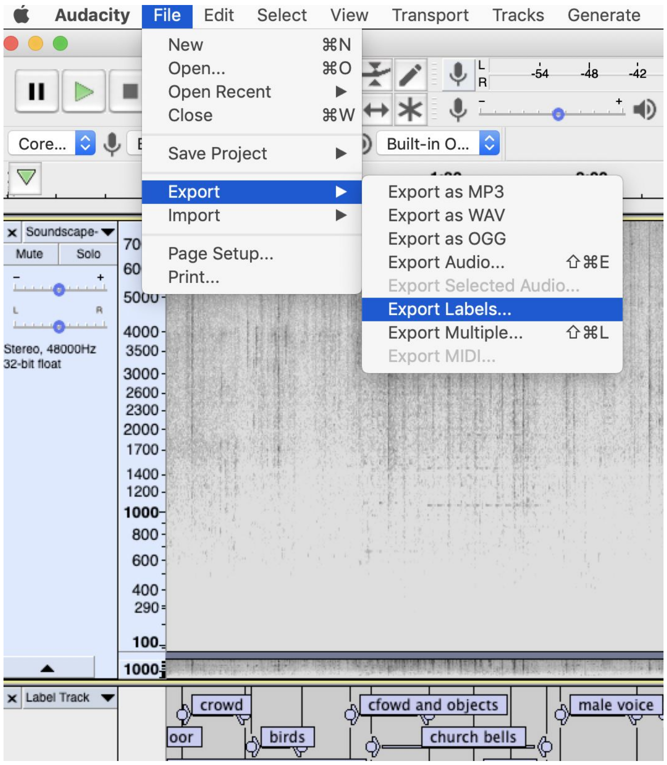

Figure 9a. Export all labels in a text file.

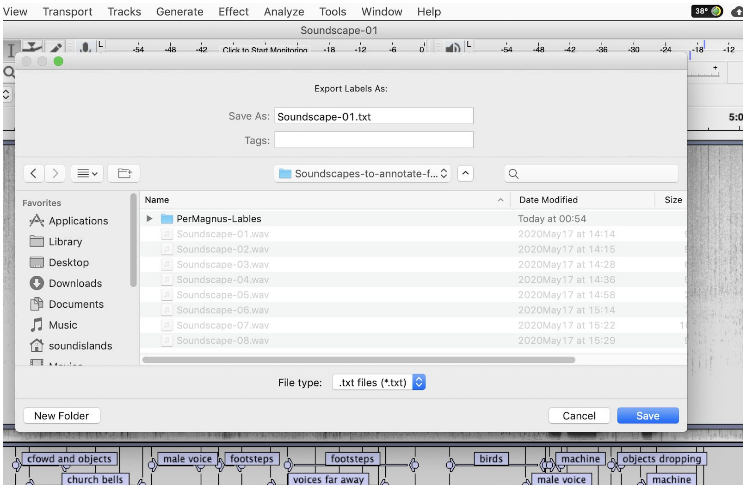

Figure 9b.

Annotations ==> .txt file

If you open the text file, it might look like in Figure 10. If you spot any spelling errors among your labels, kindly fix them here!

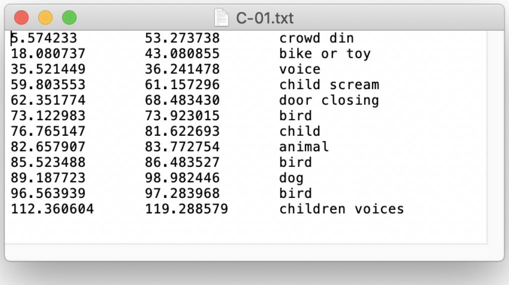

C-01.txt

5.574233	53.273738	crowd din
18.080737	43.080855	bike or toy
35.521449	36.241478	voice
59.803553	61.157296	child scream
62.351774	68.483430	door closing
73.122983	73.923015	bird
76.765147	81.622693	child
82.657907	83.772754	animal
85.523488	86.483527	bird
89.187723	98.982446	dog
96.563939	97.283968	bird
112.360604	119.288579	children voices

Figure 10.

When you are happy with the results, save the text file in a safe place, and go on to the next soundscape recording. As a rule, try to approach all of the sounds with the same kind of listening mode. Ideally your annotation labels should make sense across all your files, and have as little attention bias as possible.

At the very end, having finished a bunch of soundscapes, please return all your text files (in a zip archive) via email.

Again, THANK YOU for contributing to the research project!

PerMagnus Lindborg, pm@permagnus.org
Sara Lenzi, sara.lenzi11@gmail.com
Juan Sadaba, sadaba@ehu.eus
