# Supplementary material for: Soundscape in Times of Change: Case Study of a City Neighbourhood During the COVID-19 Lockdown
Source: Front Psychol. 2021 Mar 24;12:570741. doi: 10.3389/fpsyg.2021.570741 (PMC8024535; doi:10.3389/fpsyg.2021.570741)
Supplement: Supplementary Data Sheet 5 — Pleasantness-Eventfulness plots for all the days in the study. [file Data_Sheet_5.PDF]

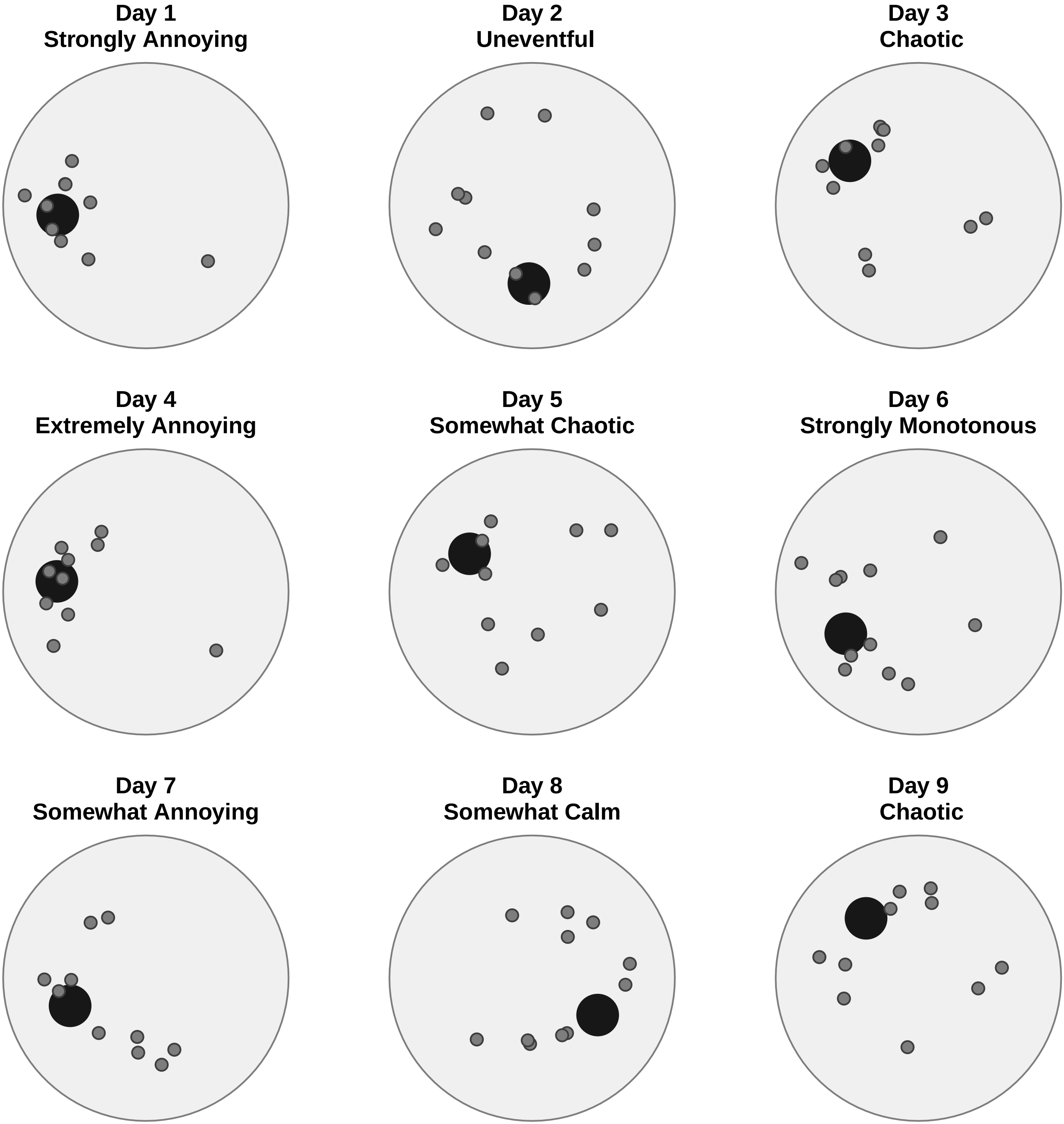
Day 1
Strongly Annoying
Day 2
Uneventful
Day 3
Chaotic
Day 4
Extremely Annoying
Day 5
Somewhat Chaotic
Day 6
Strongly Monotonous
Day 7
Somewhat Annoying
Day 8
Somewhat Calm
Day 9
Chaotic

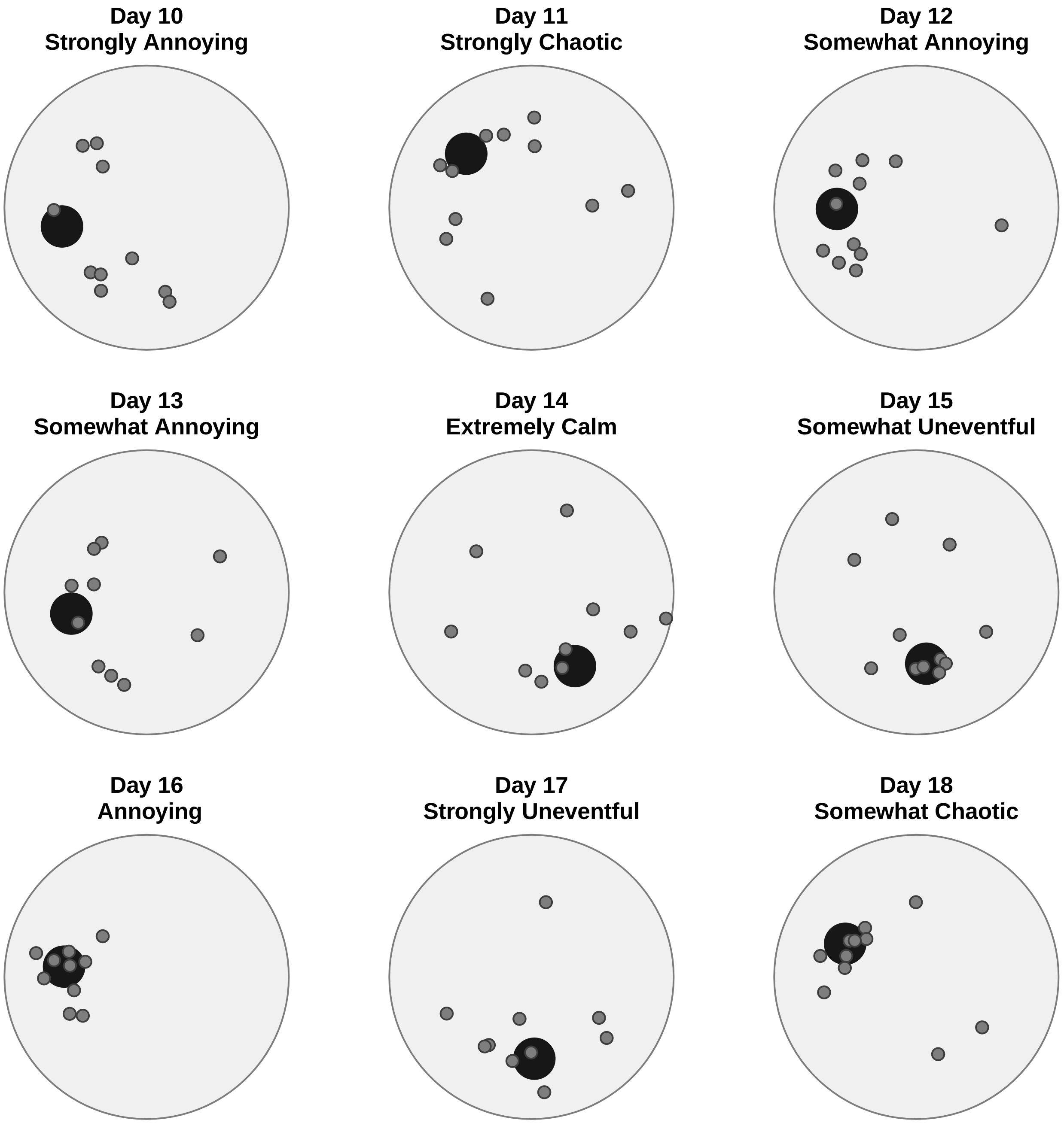
Day 10
Strongly Annoying
Day 11
Strongly Chaotic
Day 12
Somewhat Annoying
Day 13
Somewhat Annoying
Day 14
Extremely Calm
Day 15
Somewhat Uneventful
Day 16
Annoying
Day 17
Strongly Uneventful
Day 18
Somewhat Chaotic

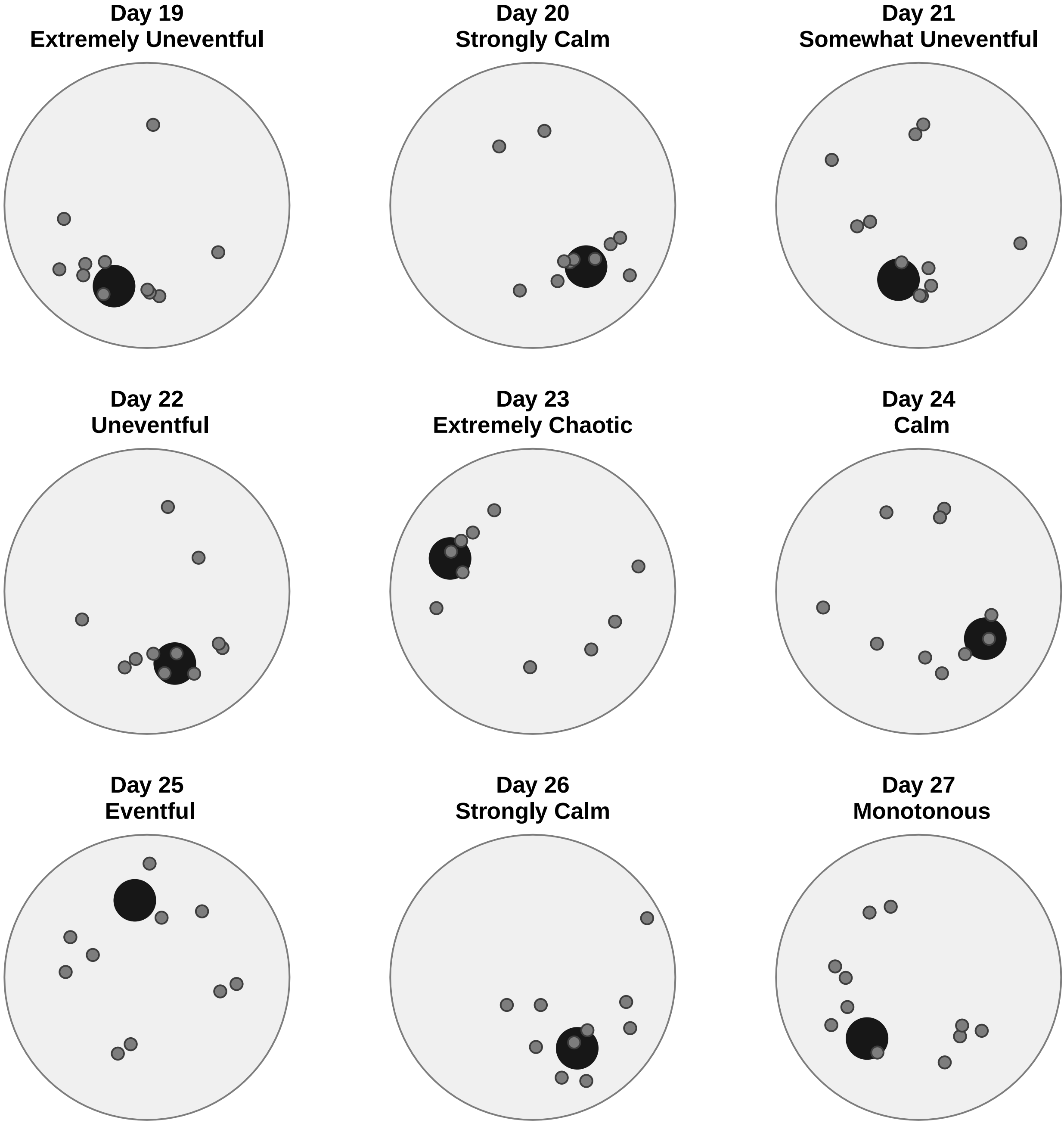
Day 19
Extremely Uneventful
Day 20
Strongly Calm
Day 21
Somewhat Uneventful
Day 22
Uneventful
Day 23
Extremely Chaotic
Day 24
Calm
Day 25
Eventful
Day 26
Strongly Calm
Day 27
Monotonous

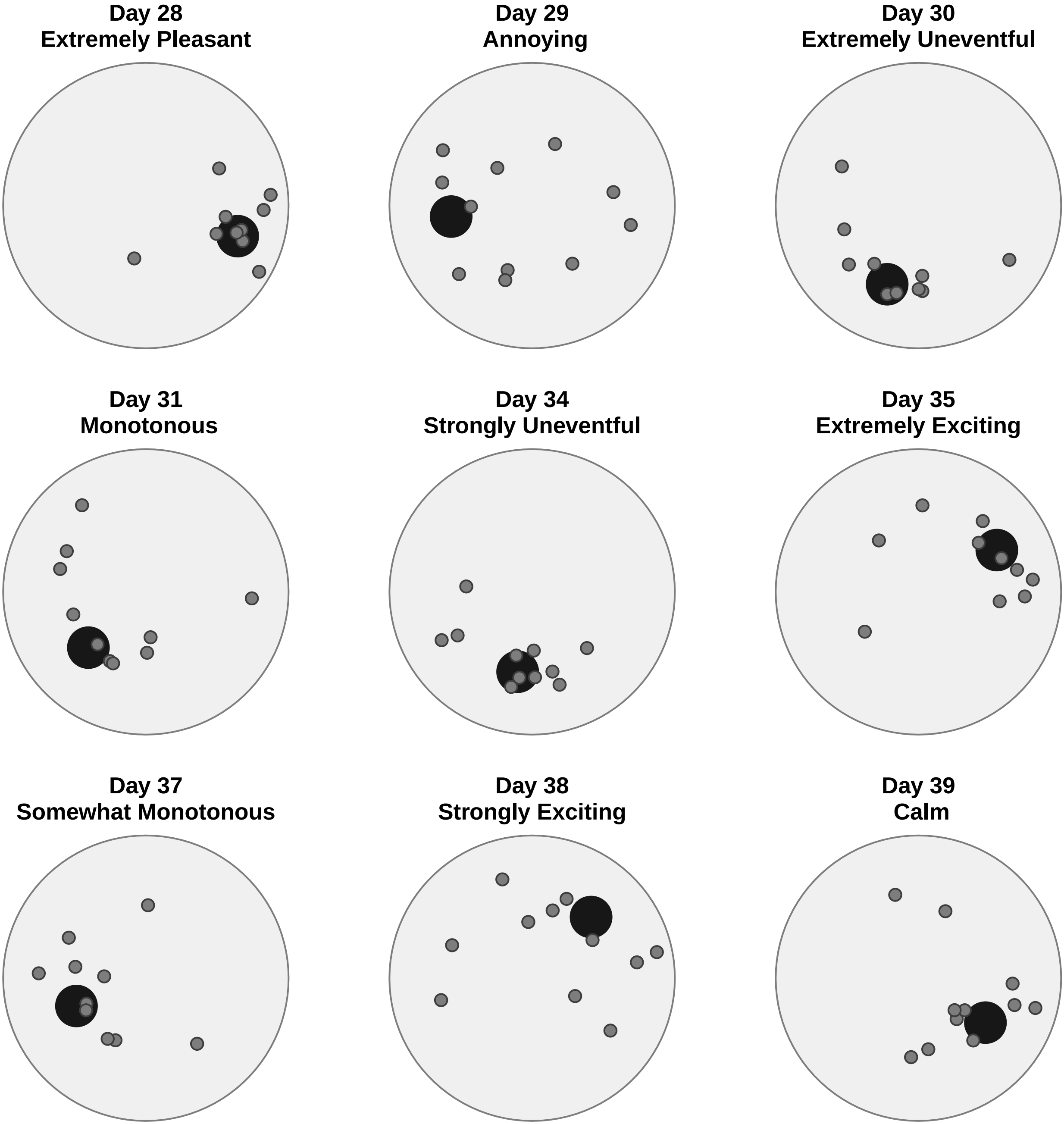
Day 28
Extremely Pleasant
Day 29
Annoying
Day 30
Extremely Uneventful
Day 31
Monotonous
Day 34
Strongly Uneventful
Day 35
Extremely Exciting
Day 37
Somewhat Monotonous
Day 38
Strongly Exciting
Day 39
Calm

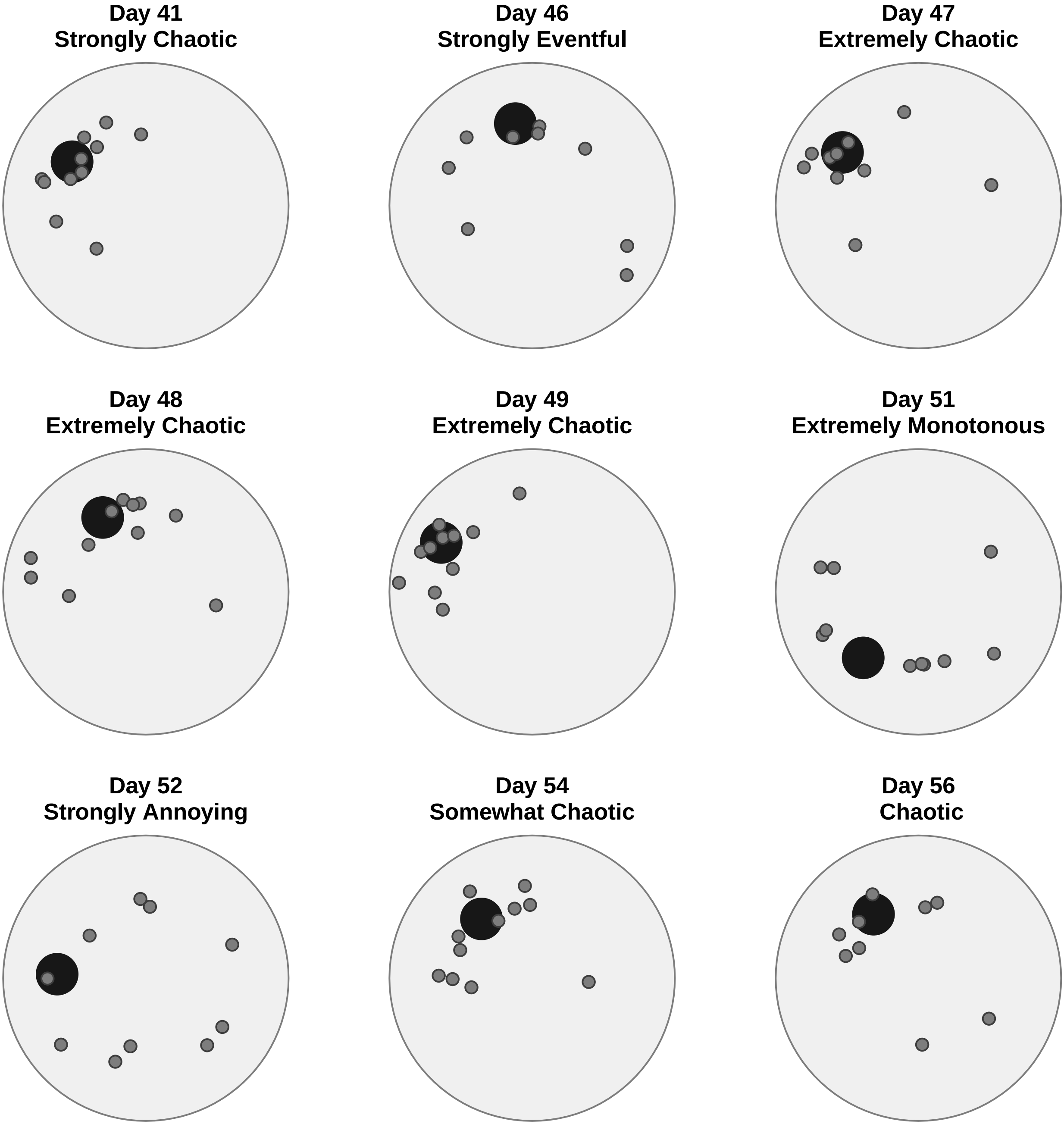
Day 41
Strongly Chaotic
Day 46
Strongly Eventful
Day 47
Extremely Chaotic
Day 48
Extremely Chaotic
Day 49
Extremely Chaotic
Day 51
Extremely Monotonous
Day 52
Strongly Annoying
Day 54
Somewhat Chaotic
Day 56
Chaotic

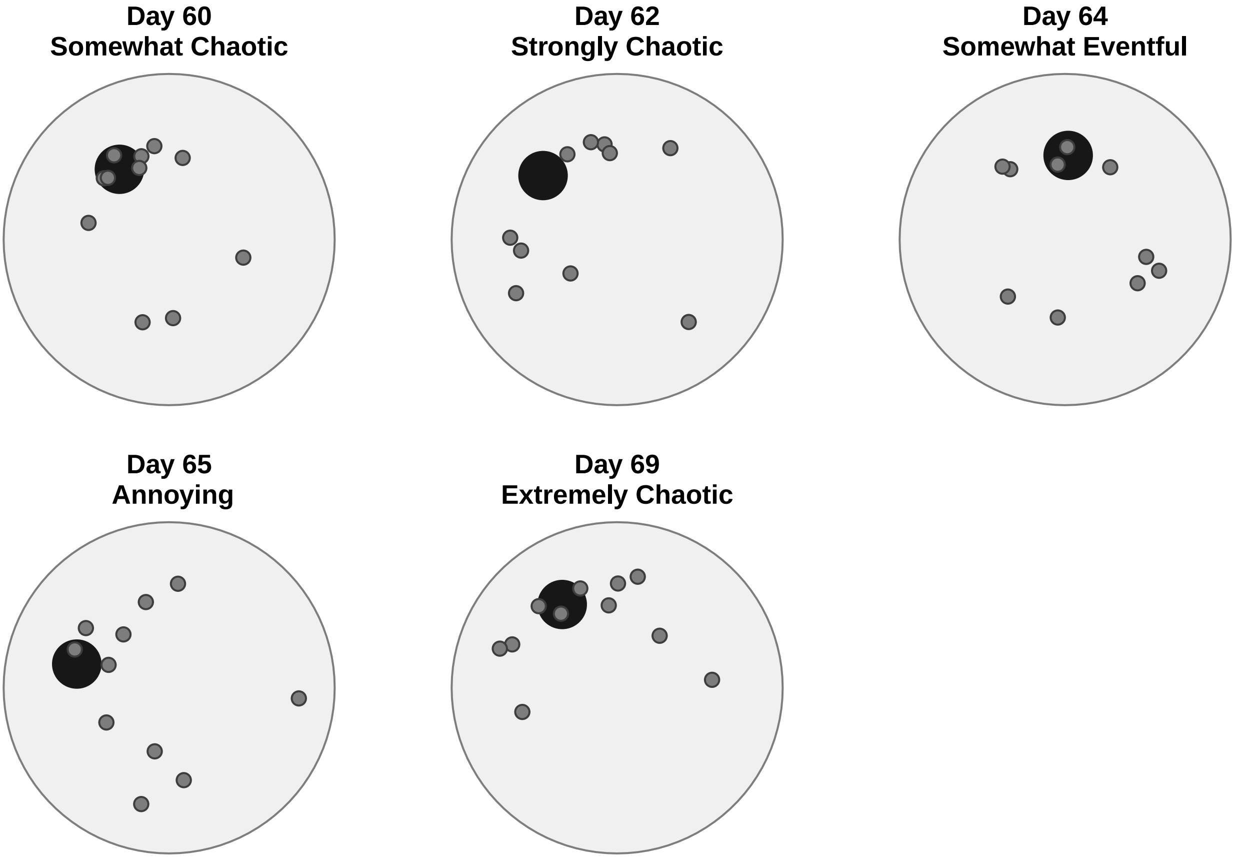
Day 60
Somewhat Chaotic
Day 62
Strongly Chaotic
Day 64
Somewhat Eventful
Day 65
Annoying
Day 69
Extremely Chaotic
