# Supplementary material for: Soundscape in Times of Change: Case Study of a City Neighbourhood During the COVID-19 Lockdown
Source: Front Psychol. 2021 Mar 24;12:570741. doi: 10.3389/fpsyg.2021.570741 (PMC8024535; doi:10.3389/fpsyg.2021.570741)

Evaluation of the soundscapes

Thank you for participating in the Expert Group!

This study was approved by the Research Ethics Committee of City University of Hong Kong, ref. 13-2020-08-E. For details, contact PerMagnus at pm@permagnus.org.

Your ID

You are given an ID. If you are "K", then the sounds you are listening to are named "K-01.mp3", "K-02.mp3" etc. The files that you will be sending back are automatically named: "K-01.txt", "K-02.txt" etc. (So if you are J, your sounds are named "J-01.mp3" etc, and your responses are "J-01.txt" etc.)

The task

You will have a set of 50 soundscape recordings (same as before). They are taken at the same location but at different points in time. They are presented randomly so you will not know the order in which they were made. You will be using a script called "Soundscape-Eval.maxpat" which runs under the software Max. The procedure is very easy this time. You listen with headphones and at the same move your computer mouse to indicate how you feel about the soundscape. You can pause at any time. Doing all 50 soundscapes takes ~100 minutes.

Make sure you have:

- A quiet environment and a computer;
- Good headphones (preferably studio-quality covering the ears fully; avoid earbuds);
- Comfortable volume (don't change it underways);
- Calm of spirit.

Setting up

The software has been tested on Mac with three different computers. You might run into problems if you are using OS 10.15 (Catalina). If you use Windows please contact PerMagnus at pm@permagnus.org.

1. Download and install Max from here: https://cycling74.com/downloads. See Figure 1. It is available for Mac and Windows, and free to use for 30 days.
2. Download the *whole* folder "Soundscape-Eval" from here: https://drive.google.com/drive/folders/17-CzZI8VRcKIigRDxxXNq_svVvL56Ark?usp=sharing. See Figure 2. Put it anywhere on your computer.
3. Download your sub-folder from here (that is, sounds corresponding to your ID): https://drive.google.com/drive/folders/1ErbMahqCIRe80JoCoCV2EcYHyUp_xY_o?usp=sharing You will use the same sounds as before so if you have saved them there's no need to download again :)

4. Put the sound-folder into the evaluation-folder.
5. Your evaluation-folder should now look something like Figure 3.

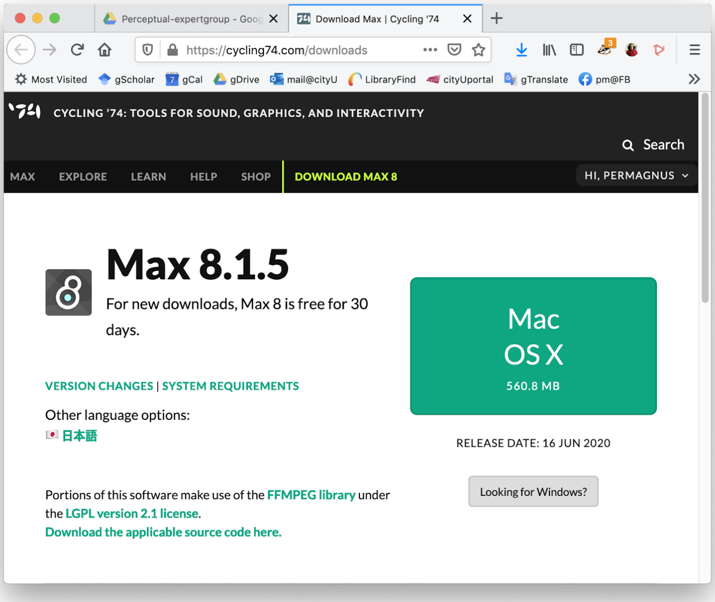

Figure 1. Max download page

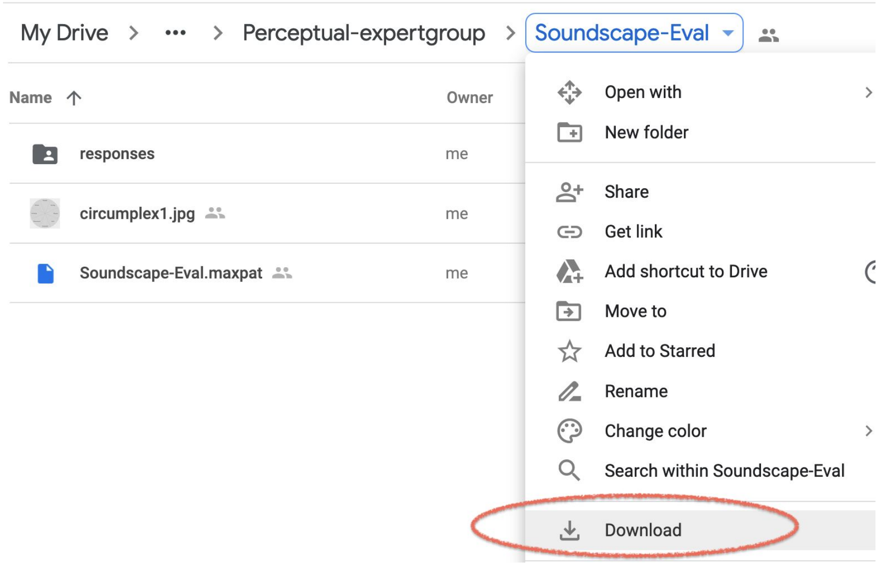

Figure 2. Make sure you download whole folder “Soundscape-Eval”

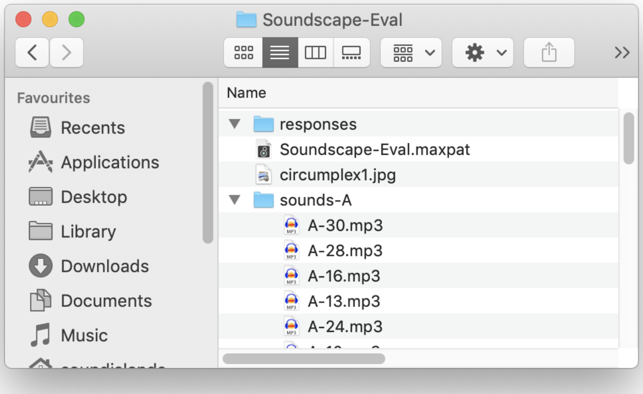

Figure 3. After unzipping, put things together to look something like this. The folder "responses" is empty to start with.

Listening and evaluating

- Double-click on "Soundscape-Eval.maxpat". It should look like in Figure 4.
- Note the 'circumplex' wheel to the left. The words indicate a feeling or mood that may describe how you feel about the soundscape at any point. Move the computer mouse within the wheel, and you see a blue 'dot'. If you click (and drag), it turns yellow.
- Before you start, adjust the volume to a comfortable level, and keep it the same level all the way through.
- Note the 'loudspeaker icon' at the lower right (and the small blue in the lower right corner). They should be 'lit up' to show that Max can play sound.

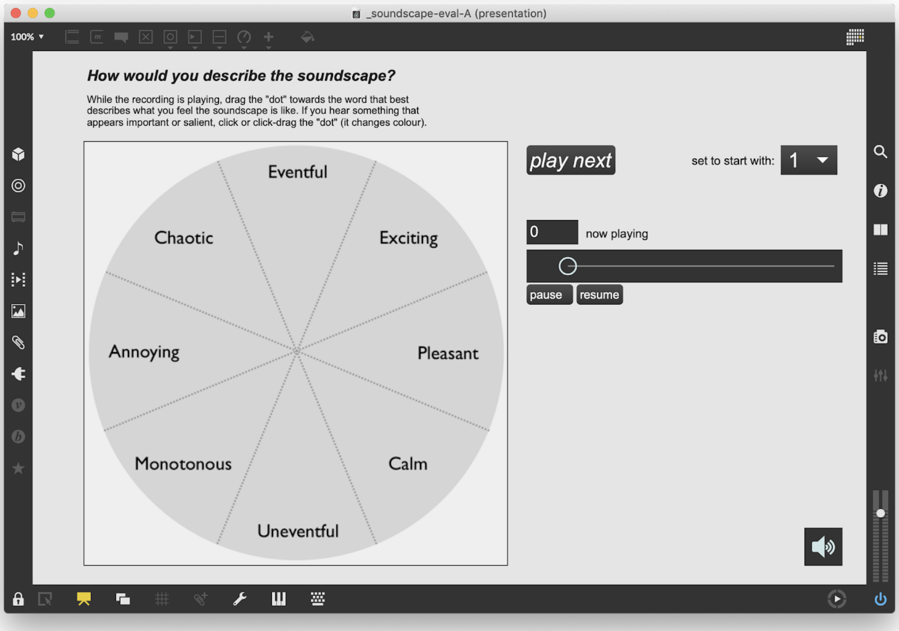

Figure 4. The patcher window.

Doing the evaluation

- To start listening, click the button "play next', and Max will play the first soundscape.
- While listening, indicate with the mouse blue dot which word describes the soundscape best. You can move around gradually when the soundscape changes. If you hear something that stands out, click (and drag) with a yellow dot.
- The recording stops automatically after two minutes. Click 'play next' when you are ready.
- You can click 'pause' and 'resume' at any point.

Stopping and continuing later

- If you want to stop and take a longer break, quit the application (command-Q).
- When coming back later, double-click on "Soundscape-Eval.maxpat" to open as before, and use the "set to start with" pulldown menu to continue from where you left off.
- You might want to use the 'set' feature if you have been trying around (with the first soundscape) and want to start it again properly from the beginning.

Checking your responses

- Your responses are kept in the folder "responses", and the data look like in Figure 5.
- When you have finished, zip the 'responses' folder and return to PerMagnus at pm@permagnus.org.

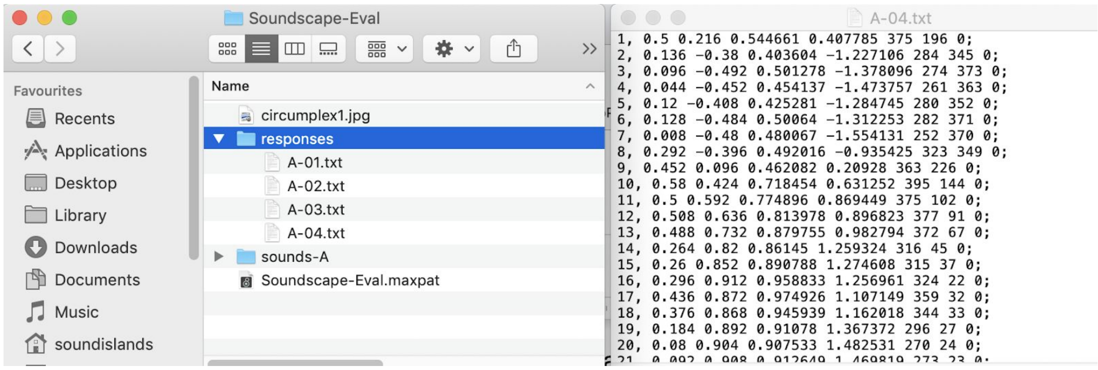

Figure 5. Your responses.

That's all, Folks!

Bravo, you're done!
THANK YOU for contributing to the research project, again!

PerMagnus, pm@permagnus.org
Sara, sara.lenzi11@gmail.com
Juan, sadaba@ehu.eus

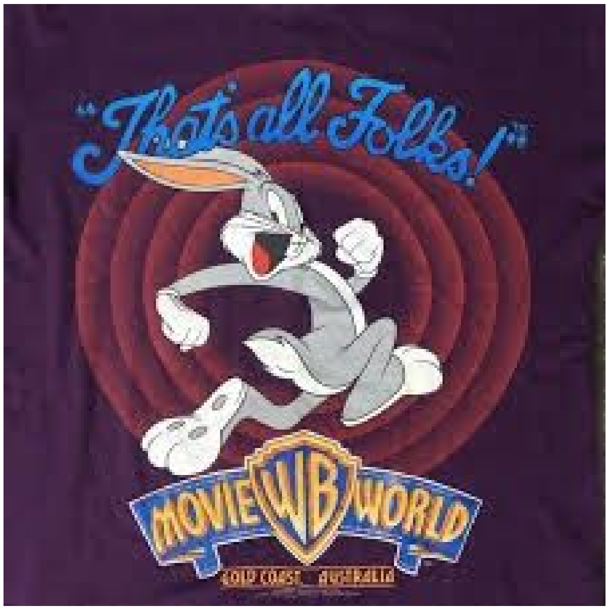

Supplement: Supplementary Data Sheet 6 — Instructions to the expert group for soundscape evaluations. [file Data_Sheet_6.PDF]
