# Supplementary material for: Soundscape in Times of Change: Case Study of a City Neighbourhood During the COVID-19 Lockdown
Source: Front Psychol. 2021 Mar 24;12:570741. doi: 10.3389/fpsyg.2021.570741 (PMC8024535; doi:10.3389/fpsyg.2021.570741)
Supplement: Supplementary Data Sheet 7 — Annotations and levels of the sound source taxonomy (in pdf format). [file Data_Sheet_7.PDF]

Day	Label_raw	Level_0	Level_1	Level_2	Level_3
1	seagull (9), birds (4), man voice, man (3), rattle, siren, voices, car engine (2), car swoosh, door slam, human voices, male voice, man shouting, sea gull, seagulls, distant, whistle, man voice (1), acoustic alarm, alarm (police?), ambulance, another vehicle passing by, bird, bump, chatting, door, engine in neutral, engine rising (van on a ramp), engine running, engine, idle, engine, van, close, female voice, hit, human voices, male female, close, indistinct voices, loud engine noise, men talking, metallic sound, noise of a van, people talking, police alarm, rubbing (someone is sweeping?), seagull, close, seagulls, softer engine noise, step, stuffs hit, thud, van parked with engine running, van parked, engine still running, vehicle speeds up&down, vehicle stops with engine on, very far car, very loud van engine passing close, voices in background, weak chirping in the distance, wind on mike (1),	seagull (9), man voice (5), birds (4), man (3), rattle, siren, voices, car engine (2), car swoosh, door slam, human voices, male voice, man shouting, sea gull, seagulls distant, whistle, acoustic alarm (1), alarm police, ambulance, another vehicle passing by, bird, bump, chatting, door, engine idle, engine in neutral, engine rising van on a ramp, engine running, engine van close, female voice, hit, human voices male female close, indistinct voices, loud engine noise, men talking, metallic sound, noise of a van, people talking, police alarm, rubbing someone is sweeping, seagull close, seagulls, softer engine noise, step, stuffs hit, thud, van parked engine still running, van parked with engine running, vehicle speeds up&down, vehicle stops with engine on, very far car, very loud van engine passing close, voices in background, weak chirping in the distance, wind on mike (1),	bird (21), vehicle (19), communication (15), signal (7), action (6), crowd (5), conversation (3), individual, object, onomatopoeia (2), body (1), geophony, material (1),	traffic (26), nature (22), voice (19), physical (10), people (8), sonic (2),	human (27), technological (26), natural (22),
2	church bells (12), church bell (9), birds (5), car passing (4), Church bells, dog barking, car (3), door closing, hits, soft, voice, chatting (2), church bell), dog, door slam, rain, choral (1), ba-doum loud closing of a door (?), bird, bird song, birds (distant), birds chirping, distant, birds faint, birds presence , boum loud (distant) bash, car (distant), car accelerating away, car brake , Car engine, car on a ramp, car swoosh, car van , choir, choral, chorus, church bell , church bell (single pitch Eb), distantnoise, dog barking (distant), dog yelp, Dogs barking in the distance, door slam , female talking while walking by, female voice chatting at phone?, footsteps, footsteps, soft, gate closing, hit, soft, human voice, close, keys, male voices, maybe it is raining, music, music by the wind, music from afar, music playing, musical instrument, people talking, scream (distant), Singing in the distance, singing voice, someone talking, Someone talking, Something falls, sound of bunch of keys, thuds (near mic), Traffic, truck coming in, truck passing by, truck, distant, truck, medium distance, unload cargos/door shut, woman (1),	church bells (16), church bell (12), birds (5), car passing (4), dog barking, car (3), door closing, door slam, hits soft, voice, chatting (2), choral, dog, rain, someone talking, ba doum loud closing of a door (1), bird, bird song, birds chirping distant, birds distant, birds faint, birds presence, boum loud distant bash, car accelerating away, car brake, car distant, car engine, car on a ramp, car swoosh, car van, choir, chorus, church bell single pitch eb, distantnoise, dog barking distant, dog yelp, dogs barking in the distance, female talking while walking by, female voice chatting at phone, footsteps, footsteps soft, gate closing, hit soft, human voice close, keys, male voices, maybe it is raining, music, music by the wind, music from afar, music playing, musical instrument, people talking, scream distant, singing in the distance, singing voice, something falls, sound of bunch of keys, thuds near mic, traffic, truck coming in, truck distant, truck medium distance, truck passing by, unload cargos door shut, woman (1),	signal (29), vehicle (19), bird (11), animal (9), music, object, conversation (7), action (6), communication (5), crowd (3), geophony, body (2), noise, onomatopoeia, individual (1),	traffic (48), nature (23), physical (15), voice (14), sonic (13), people (4),	technological (48), natural (23), human (18),
3	birds (13), bird (8), bird trill (4), music, birds chirping (3), dishes, door closing, woman voice, bump (2), electric saw, footsteps, hit, metal noise, mixed choir, soft hit, steps, traffic passing, woman, chorale (1), (car) door closing, 2nd woman speaking, alarm (reverse gear), background engine or tools noise, bash, bash car door, bash door, beep, bird chirp, bird chirps , bird song, birds and voices almost muttered, birds, distant, bus swoosh, car door opening/closing, car engine, car in background, car swoosh, car swoosh(`distant), car, distant, cars, distant, chainsaw, chatting, choir, choir females, choir singing, chorale + instrument music, chorus, clash metal objects, click, decreasing sirework noise, door close, drop metal, elder femaile speaking and laughing, electric bass line (or organ), electric saw decay, engine, engine slowing down, far conversations, female voices, female voices and birds, footstep, footsteps , hammer, heavy tool cutting metal, hitting, human chatting in background, human voice, human voices, distant, human voices, female, close, indistinct nature sound weaves, indistinct non human , laugh, low hum, music?, machine, machine drill, machine turned off, melody, nice, like palyng background music, metal clang, metal drop, metal object dropping, metal objects, metal rod, metal scratching, metal shuffle, metal tools, metalic tools , music bass, music in the air, music singing, noise, rattling, saw, saw winding down, senior lady talking, signal sound, singing, singing voices (far), something hit, song, sound of cutlery, starts music, steps and talking, strong mechanic chainsaw cutting , truck, very loud noisy circular saw, walking, woman laughs, woman speaking, woman talking very clearly, woman talking, background music, working noise (grinder) (1),	birds (13), bird (8), bird trill (4), music, birds chirping (3), dishes, door closing, footsteps, woman voice, bump (2), electric saw, hit, metal noise, mixed choir, soft hit, steps, traffic passing, woman, 2nd woman speaking (1), alarm reverse gear, background engine or tools noise, bash, bash car door, bash door, beep, bird chirp, bird chirps, bird song, birds and voices almost muttered, birds distant, bus swoosh, car distant, car door closing, car door opening closing, car engine, car in background, car swoosh, car swoosh distant, cars distant, chainsaw, chatting, choir, choir females, choir singing, chorale, chorale instrument music, chorus, clash metal objects, click, decreasing sirework noise, door close, drop metal, elder femaile speaking and laughing, electric bass line or organ, electric saw decay, engine, engine slowing down, far conversations, female voices, female voices and birds, footstep, hammer, heavy tool cutting metal, hitting, human chatting in background, human voice, human voices distant, human voices female close, indistinct nature sound weaves, indistinct non human, laugh, low hum music, machine, machine drill, machine turned off, melody nice like palyng background music, metal clang, metal drop, metal object dropping, metal objects, metal rod, metal scratching, metal shuffle, metal tools, metalic tools, music bass, music in the air, music singing, noise, rattling, saw, saw winding down, senior lady talking, signal sound, singing, singing voices far, something hit, song, sound of cutlery, starts music, steps and talking, strong mechanic chainsaw cutting, truck, very loud noisy circular saw, walking, woman laughs, woman speaking, woman talking background music, woman talking very clearly, working noise grinder (1),	bird (33), music (20), object (16), vehicle (15), action (12), communication (9), conversation, noise (8), body (7), machine, material (5), crowd (4), onomatopoeia, signal (3), individual (2),	nature (33), physical, sonic (32), traffic (25), voice, people (6),	natural (33), human (31), technological (25),

Day	Label_raw	Level_0	Level_1	Level_2	Level_3
4	birds (6), close loud hitting (3), hammer, whistling, bird (2), bird chirping, bird chirps, bump, Door, footsteps, hammer, higher pitch, Hammering, Hard hammering, people talking, voices, indistinct human and non human noise (1), bash, bird's tweet, birds chirping, bus slowing down, car, car engine, close, car swoosh and engine, cars, Chirping bird, Conversation between man and woman, door close, door open, drone of airplane, electric tool, Heavy vehicle passing nearby, hits and bumps, hitting metallic and people chatting, hitting with a hammer, human conversations, human voices, dialogue, close, Knock, knocking , Light traffic noise, machine, machine , male voice, mumbling, man and woman in conversation, man whistling, man woman talking, metal, metal hit, metal or hard material working sound, metal or hard working sound , metal working sound, more hitting closer, Object rubbing, people conversation, people laughing, People talking and birds, polple talking and laughting, power tool winding down, rattle, rhythmic hitting in distance, scrape door (?), somebody whistling while walking, people talking, steps, strong rattling sounds, sunblinds, thuds, Tram arrival, voices adults, whistle, Whistle, whistle melody, whistling a melody, work tools sound. hammering and alike (1),	birds (6), close loud hitting (3), hammer, whistling, bird (2), bird chirping, bird chirps, bump, door, footsteps, hammer higher pitch, hammering, hard hammering, machine, people talking, voices, whistle, bash (1), birds chirping, birds tweet, bus slowing down, car, car engine close, car swoosh and engine, cars, chirping bird, conversation between man and woman, door close, door open, drone of airplane, electric tool, heavy vehicle passing nearby, hits and bumps, hitting metallic and people chatting, hitting with a hammer, human conversations, human voices dialogue close, indistinct human and non human noise, knock, knocking, light traffic noise, male voice mumbling, man and woman in conversation, man whistling, man woman talking, metal, metal hit, metal or hard material working sound, metal or hard working sound, metal working sound, more hitting closer, object rubbing, people conversation, people laughing, people talking and birds, polple talking and laughting, power tool winding down, rattle, rhythmic hitting in distance, scrape door, somebody whistling while walking people talking, steps, strong rattling sounds, sunblinds, thuds, tram arrival, voices adults, whistle melody, whistling a melody, work tools sound hammering and alike (1),	bird (15), object (14), communication (13), conversation (12), action (9), vehicle, noise (7), onomatopoeia, body (3), machine, material (1), music (1),	voice (28), physical (24), nature (15), sonic, traffic (12),	human (28), natural (15), technological (12),
5	bird (3), door (2), footsteps, wind puffs in mic, Background voices (1), bells, Bells, bird's tweet, Bottel or glassmade object dropping, bump, carillon or similar, chatting, church bell, church bells twice, clicks, clinking, Conversation, Conversation between a man and a woman, conversation continues, dishes, distant church bells, dog barking, door, distant, Far away voices, flute music, gate open, glass/ceramic plates, hit small metal objets, hit wood, hit, distant, hollow vent noise, human voices, dialogue, female, closer, human voices, distant, laugh, Laugh, laughter, laughter, exhalation, Light traffic noise, machine, machinery, something turning, man and woman chatting, man and woman conversation, man and woman conversation (walking passing by, metal scrape noises, music, music whistle, object on the floor, paper bag, People moving, people talking, radio music, rattling (glass dropping), rubber sole on the ground, screech, rubber sole?, shoe rubbing against the floor, shuffling (plastic bag?), Somebody playing txistu music, steps, toy bell sound, toy music, traffic, traffic passing, truck, voice mumbling, walk with plastic bag, wind or air movement, woman laughs, Young Child talking (1),	bird (3), bells (2), door, footsteps, laugh, wind puffs in mic, background voices (1), birds tweet, bottel or glassmade object dropping, bump, carillon or similar, chatting, church bell, church bells twice, clicks, clinking, conversation, conversation between a man and a woman, conversation continues, dishes, distant church bells, dog barking, door distant, far away voices, flute music, gate open, glass ceramic plates, hit distant, hit small metal objets, hit wood, hollow vent noise, human voices dialogue female closer, human voices distant, laughter, laughter exhalation, light traffic noise, machine, machinery something turning, man and woman chatting, man and woman conversation, man and woman conversation walking passing by, metal scrape noises, music, music whistle, object on the floor, paper bag, people moving, people talking, radio music, rattling glass dropping, rubber sole on the ground, screech rubber sole, shoe rubbing against the floor, shuffling plastic bag, somebody playing txistu music, steps, toy bell sound, toy music, traffic, traffic passing, truck, voice mumbling, walk with plastic bag, wind or air movement, woman laughs, young child talking (1),	conversation (10), object (7), action (6), music, vehicle, body (5), communication, signal, bird (4), crowd, geophony (3), noise, onomatopoeia, machine (2), material, animal (1), group (1),	voice (20), physical (15), traffic (13), sonic (12), nature (8), people (5),	human (25), technological (13), natural (8),
6	dog barking (13), bird (6), footsteps, dog (5), steps, door closing (4), birds (3), exotic animal, hit, airplane (2), background conversations, child, child crying, dog bark, high pitched whistle, human voices, keys, man voice, aeroplane (1), airplane passing, loud, Doppler-down, airplane, faint, background rumbling, bird chirping, birds chirping, burds, car, car passing, child cry , child speak-cry, child tantrum, child voice, children, children speaking, dishes, distant and close voices, distant human voices, distant voices, dog yelp, door opening, dropping objects, increasing and decreasing rubling (airplane), jet fly passing, key rattling, keys rattling, kid whining, kids playing, male voice, men, people speaking, people talking, plane, plane passing, scan noise, soft background city traffic noise, traffic, voice, woman talking, woman voice, woman walking (1),	dog barking (13), bird (6), footsteps, dog (5), steps, door closing (4), birds (3), exotic animal, hit, airplane (2), background conversations, child, child crying, dog bark, high pitched whistle, human voices, keys, man voice, aeroplane (1), airplane faint, airplane passing loud doppler down, background rumbling, bird chirping, birds chirping, burds, car, car passing, child cry, child speak cry, child tantrum, child voice, children, children speaking, dishes, distant and close voices, distant human voices, distant voices, dog yelp, door opening, dropping objects, increasing and decreasing rubling airplane, jet fly passing, key rattling, keys rattling, kid whining, kids playing, male voice, men, people speaking, people talking, plane, plane passing, scan noise, soft background city traffic noise, traffic, voice, woman talking, woman voice, woman walking (1),	animal (24), vehicle (14), bird (12), body, communication (11), object (8), action (7), conversation, crowd, individual (3), group (1), noise (1),	nature (36), voice (30), physical (15), traffic (14), people (11), sonic (1),	human (41), natural (36), technological (14),
7	child voice (4), barking (3), beep, bird, birds, dog barking, Dog barking, men talking, bang (2), car, cart, dog bark, footsteps, little dog, male voice, man, men speaking, People talking, Traffic noises, traffic passing, whisper, bashing (1), birds in background, bumps, car in the distance, cart rolling, child cries, child cry, child cry (faint), child hum, child humming, click, Construction noises, delivery cart rolling loudly, distant cars, traffic, distant voice humming, steps, dog bark twice, door, door closing, door faint, door opening, female or child voice, human male voices chatting, human voice, male, close, kids, distant voices, littlr dog, male voices, man speaking loudly, noise cart and door slam, noise cart in background, obects bashing (faint), paper caumbling, thud, voice sings, woman or child humming (1),	dog barking (6), child voice (4), barking (3), beep, bird, birds, men talking, bang (2), car, cart, dog bark, footsteps, little dog, male voice, man, men speaking, people talking, traffic noises, traffic passing, whisper, bashing (1), birds in background, bumps, car in the distance, cart rolling, child cries, child cry, child cry faint, child hum, child humming, click, construction noises, delivery cart rolling loudly, distant cars traffic, distant voice humming steps, dog bark twice, door, door closing, door faint, door opening, female or child voice, human male voices chatting, human voice male close, kids distant voices, littlr dog, male voices, man speaking loudly, noise cart and door slam, noise cart in background, obects bashing faint, paper caumbling, thud, voice sings, woman or child humming (1),	animal (15), communication (13), conversation (11), vehicle (9), bird (7), onomatopoeia (6), object (5), wheels, body (3), crowd, individual, signal, machine (1), material (1),	voice (27), nature (22), traffic (18), people (6), physical, sonic (6),	human (33), natural (22), technological (18),

Day	Label_raw	Level_0	Level_1	Level_2	Level_3
8	birds (4), birds chirping (3), footsteps, male voice, seagull, shaker, voices, child (2), coughing, dog steps, kids, Knock, seagulls, traffic, traffic passing, (blank) (1), backgroudn traffic sound, background conversations, Background murmur, bird chirping, Birds chirping, birds sing all time, boy shouting, bumps, Car, car in back ground, car in background, car passing, car, distant, child screaming, child voice, children, close audible conversations, dog footsteps, dog walking, dog walking , female shouting, footsteps , Girl shouting, girl singing, human steps, human voice, male, close, moving, human voices, distant, kid shouting, loud male voice, male voice , male voice 2, man, man talking, Man talking. Conversation, motorbike, object boum, objects, people talking, Seagull, teeming with domestic voices and sounds from homes, thumps, Traffic noise, vehicle, voice of a little child very far, voices , wind gust, woman (1),	birds (4), birds chirping, footsteps, male voice, seagull, voices, shaker (3), child (2), coughing, dog steps, dog walking, kids, knock, seagulls, traffic, traffic passing, backgroudn traffic sound (1), background conversations, background murmur, bird chirping, birds sing all time, blank, boy shouting, bumps, car, car distant, car in back ground, car in background, car passing, child screaming, child voice, children, close audible conversations, dog footsteps, female shouting, girl shouting, girl singing, human steps, human voice male close moving, human voices distant, kid shouting, loud male voice, male voice 2, man, man talking, man talking conversation, motorbike, object boum, objects, people talking, teeming with domestic voices and sounds from homes, thumps, traffic noise, vehicle, voice of a little child very far, wind gust, woman (1),	bird (16), communication, vehicle (12), body (7), individual, animal (5), conversation, crowd, onomatopoeia (4), action (3), object, geophony (1), noise, rest (1),	voice (28), nature (22), people (12), traffic, physical (6), sonic (5), modifiers (1),	human (40), natural (22), technological (12),
9	dog barking (7), birds (5), door (4), airplane (3), bird, bird chirping, bird singing, bird tweeting, click, door, door closing, footsteps, Bird chirping (2), birds chirping, Birds chirping, birds intense, bump, Dog barking, Door, Door closes, door opening, gate opening and closing, hit, human voices, distant, people chatting, steps, thud, voices, a person walking (1), aeroplane sound, airplane (doppler downwards), Airplane flying overhead, airplane taking off, background conversations, background traffic and doors slamming sounds, barking, Barking dog, beep, birds chirring, Chirping birds, click, door locker, dog, dogs barking, door , Door closing, door locked, door shutting, Doors closing, Far away voice, Footsteps, gate, gate opening, hit, low pitch, hits, indistinct human voice, jet fly pass, laughter, loud door, loudest part of plane flyover, male voice (faint), men talking, micrhophone manipulation, mower (?), Noise, People talking, Plane taking off, radio, soft noise, Something shuts, Steps, steps (someone is running?), steps, movement, Traffic noise (1),	dog barking (9), door (7), bird chirping (5), birds, birds chirping (4), door closing, footsteps, airplane (3), bird, bird singing, bird tweeting, click door, steps, birds intense (2), bump, door closes, door opening, gate opening and closing, hit, human voices distant, people chatting, thud, voices, a person walking (1), aeroplane sound, airplane doppler downwards, airplane flying overhead, airplane taking off, background conversations, background traffic and doors slamming sounds, barking, barking dog, beep, birds chirring, chirping birds, click door locker, dog, dogs barking, door locked, door shutting, doors closing, far away voice, gate, gate opening, hit low pitch, hits, indistinct human voice, jet fly pass, laughter, loud door, loudest part of plane flyover, male voice faint, men talking, micrhophone manipulation, mower, noise, people talking, plane taking off, radio, soft noise, something shuts, steps movement, steps someone is running, traffic noise (1),	bird (27), object (26), animal (13), vehicle (11), body (10), action (7), conversation (5), crowd, onomatopoeia (4), communication (3), noise (2), machine (1), music, rest, signal (1),	nature (40), physical (33), voice (18), traffic (13), sonic (7), people (5), modifiers (1),	natural (40), human (23), technological (13),
10	birds (14), male voice (4), bird (2), birds chirping, cart, circular saw, Construction noises, door closing, electric saw , keys, male voice, closer, metal scratching, People talking, strong electric tool, alarm (1), background human conversations, bash, bashing (plates?), bicycle or cart, bump, car accelleration, car swoosh, away, cart on a different surface, cart rolling, chatting, clack, cling, construction works, distant car, distant human voices, male/female, distant sound of neighbors, door , door (?), door close, door closing , Doors closing, electric saw, electronic beep (ATM?), kid's voice, kids voice (distant), laughter, machine, male voice (smae), metal clang, metal clicking, Metallic clanging, mobile phone (incoming message), music, object on a surface, object sliding, people talking, slams, soft background traffic , soft voice, soldering, Something being dragged, sounds of a construction site, traffic, trolley, undefined soft rattling noise, voice, voices, wheel scratching (1),	birds (14), male voice (4), door closing (3), electric saw, people talking, bird (2), birds chirping, cart, circular saw, construction noises, door, keys, male voice closer, metal scratching, strong electric tool, alarm (1), background human conversations, bash, bashing plates, bicycle or cart, bump, car accelleration, car swoosh away, cart on a different surface, cart rolling, chatting, clack, cling, construction works, distant car, distant human voices male female, distant sound of neighbors, door close, doors closing, electronic beep atm, kids voice, kids voice distant, laughter, machine, male voice smae, metal clang, metal clicking, metallic clanging, mobile phone incoming message, music, object on a surface, object sliding, slams, soft background traffic, soft voice, soldering, something being dragged, sounds of a construction site, traffic, trolley, undefined soft rattling noise, voice, voices, wheel scratching (1),	bird (18), object (14), machine (10), communication (9), wheels (7), conversation (5), crowd, material, onomatopoeia, vehicle, signal (3), action (2), group (1), music, noise (1),	traffic (25), physical (21), nature (18), voice (14), sonic (7), people (6),	technological (25), human (20), natural (18),
11	birds (10), electric bip (6), door closing (5), electronic beep (4), steps, bip (3), bird, distant traffic, door, footsteps, loud thump, slamming doors, traffic, whistle, whistling, alarm (2), alarm sound, birds chirping, can opening, car driving-by, car passing, car, closer, female voice, motorcycle, snap, super loud thump, woman talking, scan sound (1), Strong hit, 2 rattles, a thud, Background murmur, background undefined city traffic sounds, beep, beeps, horns, bicycle wheels, bike kickstand, bird , birds (intense), birds and man who whistles, birds+bruit, bump, bump (door?), car, car in background, car or motorcycle, Car passing, cart, chatting spread in the background, Chirping birds, distant music (TV), distant voices, door opening, door slam, dropping large objects, exhalation, far conversations, finger snaps, friction noise, glass decoration noise, Heavy vehicle passing nearby, hit, hits and bump (car doors?), human voice, female, indistinct people voice, indistinct traffic, indistinct traffic noise, Light steps, loud dropping objects, loud thuds, man whistleing, man whistles, Man whistles, manipulating something in a box, metallic noise, metallic sound, motorbike, motorbike passing faint, object noise, objects, opening can, people talking, Phone sound, rattling, ringtone?, rising engine, scan noise, scan sound, screaming brakes, slaming car door, soft cough, sound of can opening, Strong hit, Strong traffic noise, tailgate slam, tailgate slam , tailgate slams, Telephone sound, thud, thuds, traffic hum faint, traffic passing faint, tyre screeching?, undefined (reload of something?), undetermined sound, Unidentified knocks, Very Strong hits, voices, whisteling, whistles, Woman speaking loudly (1),	birds (10), electric bip (6), door closing (5), bird (4), electronic beep, steps, bip (3), car passing, distant traffic, door, footsteps, loud thump, slamming doors, traffic, whistle, whistling, alarm (2), alarm sound, birds chirping, can opening, car closer, car driving by, female voice, man whistles, motorcycle, scan sound, snap, strong hit, super loud thump, tailgate slam, woman talking, 2 rattles (1), a thud, background murmur, background undefined city traffic sounds, beep, beeps horns, bicycle wheels, bike kickstand, birds and man who whistles, birds bruit, birds intense, bump, bump door, car, car in background, car or motorcycle, cart, chatting spread in the background, chirping birds, distant music tv, distant voices, door opening, door slam, dropping large objects, exhalation, far conversations, finger snaps, friction noise, glass decoration noise, heavy vehicle passing nearby, hit, hits and bump car doors, human voice female, indistinct people voice, indistinct traffic, indistinct traffic noise, light steps, loud dropping objects, loud thuds, man whistleing, manipulating something in a box, metallic noise, metallic sound, motorbike, motorbike passing faint, object noise, objects, opening can, people talking, phone sound, rattling, ringtone, rising engine, scan noise, screaming brakes, slaming car door, soft cough, sound of can opening, strong traffic noise, tailgate slams, telephone sound, thud, thuds, traffic hum faint, traffic passing faint, tyre screeching, undefined reload of something, undetermined sound, unidentified knocks, very strong hits, voices, whisteling, whistles, woman speaking loudly (1),	vehicle (29), signal (22), object (21), bird (19), communication (15), onomatopoeia, body (10), action (8), material (7), noise, conversation (6), crowd (4), wheels (3), music (1),	traffic (54), physical (36), voice (31), sonic (23), nature (19), people (4),	technological (54), human (35), natural (19),

Day	Label_raw	Level_0	Level_1	Level_2	Level_3
12	birds (7), People talking (4), footsteps (3), louder voices, woman, bird (2), birds chirping, car, door shutting, human voice, female, close, kid, knocking, voices, ambulance alarm (1), ambulance siren, background din, background traffic noise, bang (tennis ball on the floor?), bckground conversations, bird song, breath, bump, bus screech, car engine, chatting, child, child voice, children voices, close voice, Distant sirens, door bash, door closing, door knocking, door opening, door slam, door slams, female 'neh!', foot stamping, footstep, Footsteps, hammering, hit, hollow vent noise, human voices, male, kids, distant, kids, kids talking, knock door, knocking door, laugh, loud conversation 3 people, loud door bang, loud female voice, machine beeping, machine buzzing, motorbike, people talking, rather loud female voice, many voices, seagulls, senior lady chatting, siren, siren faint, steps (rubber sole), Steps, rubber sole, talking, talking , thump, thumpting feet, ticking (bike heels?), ticking, bike?, traffic hum, undefined ricking, unloading goods, voice, walking with rubber shoes, wind guts, woman shout, women talking (1),	birds (7), people talking (5), footsteps (4), louder voices (3), woman, bird (2), birds chirping, car, door shutting, human voice female close, kid, knocking, steps rubber sole, talking, voices, ambulance alarm (1), ambulance siren, background din, background traffic noise, bang tennis ball on the floor, bckground conversations, bird song, breath, bump, bus screech, car engine, chatting, child, child voice, children voices, close voice, distant sirens, door bash, door closing, door knocking, door opening, door slam, door slams, female neh!, foot stamping, footstep, hammering, hit, hollow vent noise, human voices male kids distant, kids, kids talking, knock door, knocking door, laugh, loud conversation 3 people, loud door bang, loud female voice, machine beeping, machine buzzing, motorbike, rather loud female voice many voices, seagulls, senior lady chatting, siren, siren faint, thump, thumpting feet, ticking bike, ticking bike heels, traffic hum, undefined ricking, unloading goods, voice, walking with rubber shoes, wind guts, woman shout, women talking (1),	bird (13), conversation, object (12), crowd (11), body (9), communication (8), individual, signal (6), vehicle, onomatopoeia (5), action (3), noise (2), wheels, geophony (1), machine (1),	voice (30), people (19), physical (15), traffic, nature (14), sonic (7),	human (49), technological (15), natural (14),
13	birds (14), bird (6), bird chirping (5), male voice (4), trolley, people talking (3), birds chirping (2), car, cars, cough, door, male voices, adult shouting (1), baby, baby and woman shout, baby shouting, baby voice, background traffic sound, bicycle wheels rolling, bird , bird song, birds and indistinct sound, brids , car engine, car engine , car engine start, car passing by, car passing-by, car revs, car sengine, car starting engine and coming very close, car swoosh, car swoosh distant, car, distant, car, medium distance, car/van approaching a ramp, cars driving-by, cart, cart approaching, cart rolling, jinging, screeches, cart weels, chatting, child, clear conversation, click, coughing, coughing, laughter, door closing, door opening , engine (car) starter, engine idle, engine rising, engine starting, engine switching on , engine, van, close, far conversations, gate, high-pitched bird, hit, hit (car door), hit (door closing?), human voice, female, close, human voice, male, close, human voices, male, close, humans voices and conversations, male laughter, man, man talking, man woman voices crescendo, men talking, metallic noise, motorbike, medium distance, object moved on a surface, ping pong ball, plastic bag, rolling of something, scratch hollow object, scratch hollow object again, shout, small wheels, start engine, traffic passing, trolley again, trolly or alike moving, truck, van, van engine approaching, close and loud, very far car, voices male and female, wheels rolling , woman (1),	birds (14), bird (7), bird chirping (5), male voice (4), trolley, people talking (3), birds chirping (2), car, car engine, car passing by, cars, cough, door, male voices, adult shouting (1), baby, baby and woman shout, baby shouting, baby voice, background traffic sound, bicycle wheels rolling, bird song, birds and indistinct sound, brids, car distant, car engine start, car medium distance, car revs, car sengine, car starting engine and coming very close, car swoosh, car swoosh distant, car van approaching a ramp, cars driving by, cart, cart approaching, cart rolling jinging screeches, cart weels, chatting, child, clear conversation, click, coughing, coughing laughter, door closing, door opening, engine car starter, engine idle, engine rising, engine starting, engine switching on, engine van close, far conversations, gate, high pitched bird, hit, hit car door, hit door closing, human voice female close, human voice male close, human voices male close, humans voices and conversations, male laughter, man, man talking, man woman voices crescendo, men talking, metallic noise, motorbike medium distance, object moved on a surface, ping pong ball, plastic bag, rolling of something, scratch hollow object, scratch hollow object again, shout, small wheels, start engine, traffic passing, trolley again, trolly or alike moving, truck, van, van engine approaching close and loud, very far car, voices male and female, wheels rolling, woman (1),	bird (32), vehicle, communication (15), wheels (13), conversation (9), object, action (4), individual, body (3), crowd, material (2), onomatopoeia (1),	traffic (45), nature (32), voice (27), physical (15), people (7), sonic (1),	technological (45), human (34), natural (32),
14	traffic passing (6), car driving-by (3), bird (2), birds, birds chirping, car engine. loud, car, medium distance, cars, medium distance, dog barking, motorbike, close, voices faint, whistle, wind gut, bird chirping (1), bird chirping, close, birds, distant, car, car engine. medium, car engines. soft/far, car honk, car passing, car passing, closer, car swoosh, car swoosh (faint), car, distant, car? engine noise, click, dog barks, dogs barking, door slam, door thud, gate closing, hit, horn, human voice, female, laugh, human voices, shout, shout far away, squirr bird, thud, voice, laughter (1),	traffic passing (6), car driving by (3), bird (2), birds, birds chirping, car engine loud, car medium distance, cars medium distance, dog barking, motorbike close, voices faint, whistle, wind gut, bird chirping (1), bird chirping close, birds distant, car, car distant, car engine medium, car engine noise, car engines soft far, car honk, car passing, car passing closer, car swoosh, car swoosh faint, click, dog barks, dogs barking, door slam, door thud, gate closing, hit, horn, human voice female laugh, human voices, shout, shout far away, squirr bird, thud, voice laughter (1),	vehicle (26), bird (10), crowd (5), animal (4), communication, object (3), geophony (2), onomatopoeia, signal, action (1),	traffic (28), nature (16), people (5), physical (4), voice, sonic (2),	technological (28), natural (16), human (9),
15	baby (7), birds (6), car, voices, footsteps (5), keys, door closing (4), door slam, male voice, seagulls, traffic passing, undefined, children (3), hit, window shutters are raised, bird (2), bird chirps, blinds, bump, car swoosh, chatting, children voices, distant car, human voices, kid, no-human, gear, plastic bag, scratch, steps, thud, traffic, whistling, van (1), baby crying, baby voice, bang, bird , bird chirp, birds plenty, birds, traffic and childrens, birds' chirps, blinds closing sound, blinds closing sounds, brids chirping, car distant, car sound, child, child laughing, child scream, child sing , children sing, children voice, childrens talking, distant whistle, dog, dog barking, door, door bang, door closing , door locked, door opening, door opening (van or car), female voice, footstep, footsteps, male, heel shoes, hild scream, howling(?), human steps, human voice, woman, human whistle, human whistling, indistinct basic noise, ka-douk thumps, key rattling, keys rattling, kid laughing, kid screams, kid's shout, kids and parents , kids voices, kids' voices, kids' voices (distant), little scream, loud door, loud scratches, man, man and children talking, man speaking, man talking, man voice, man whistleing, men's voices (close), metallic noise, mom, open the door, raffic noise, rolling, rolling , scratching, sea gull, seagull, seagulls (as if scared by the noise), shaker, sifting, something pulled (sunblind?), song, talking, traffic noise, van, voice of a family, window sunblid , window sunblind, women voices (1),	baby (7), birds (6), car, voices, door closing (5), footsteps, keys, door slam (4), male voice, seagulls, traffic passing, undefined, bird (3), children, hit, window shutters are raised, bird chirps (2), blinds, bump, car swoosh, chatting, children voices, distant car, human voices, kid, kids voices, no human gear, plastic bag, rolling, scratch, steps, thud, traffic, van, whistling, baby crying (1), baby voice, bang, bird chirp, birds chirps, birds plenty, birds traffic and childrens, blinds closing sound, blinds closing sounds, brids chirping, car distant, car sound, child, child laughing, child scream, child sing, children sing, children voice, childrens talking, distant whistle, dog, dog barking, door, door bang, door locked, door opening, door opening van or car, female voice, footstep, footsteps male, heel shoes, hild scream, howling, human steps, human voice woman, human whistle, human whistling, indistinct basic noise, ka douk thumps, key rattling, keys rattling, kid laughing, kid screams, kids and parents, kids shout, kids voices distant, little scream, loud door, loud scratches, man, man and children talking, man speaking, man talking, man voice, man whistleing, mens voices close, metallic noise, mom, open the door, raffic noise, scratching, sea gull, seagull, seagulls as if scared by the noise, shaker, sifting, something pulled sunblind, song, talking, traffic noise, voice of a family, window sunblid, window sunblind, women voices (1),	communication (30), vehicle (27), bird (21), object, individual (18), noise (13), action (11), body, onomatopoeia (10), crowd (9), conversation (7), material (3), animal (2), music (1),	voice (48), physical (35), people (27), traffic, sonic (24), nature (23),	human (75), technological (27), natural (23),

Day	Label_raw	Level_0	Level_1	Level_2	Level_3
16	birds (5), trolley, cart (4), beep (2), birds chirping, car, cart rolling, dog bark, hits and bumps, metal object noise, music, stroller wheels, background traffic (1), bashes, bip, bird, birds chirp, birs, bump, car passing, car passing further away, car signal, cart noisy rolling further away, cart or motorcycle, cart scratch, delivery van approaching, dog barking, door, dull sound/whistle, flute. Soft, not continous, hit, indistinct human voice, indistinct non human noise, instrument music (flute?), male voice, man, man talking, man voice, music instrument, music melody, noisy chart, noisy delivery cart rolling, scan sound, sparse flute melody, stroller wheels , three loud bashes (further away), thuds, ticking (or water dripping?), ticking or water dripping, traffic, traffic passing, trumpet 3-note motif, voice, wheels hitting ground (1),	birds (5), trolley, cart (4), stroller wheels (3), beep (2), birds chirping, car, cart rolling, dog bark, hits and bumps, metal object noise, music, ticking or water dripping, background traffic (1), bashes, bip, bird, birds chirp, birs, bump, car passing, car passing further away, car signal, cart noisy rolling further away, cart or motorcycle, cart scratch, delivery van approaching, dog barking, door, dull sound whistle, flute soft not continous, hit, indistinct human voice, indistinct non human noise, instrument music flute, male voice, man, man talking, man voice, music instrument, music melody, noisy chart, noisy delivery cart rolling, scan sound, sparse flute melody, three loud bashes further away, thuds, traffic, traffic passing, trumpet 3 note motif, voice, wheels hitting ground (1),	wheels (20), bird (10), music (8), vehicle, onomatopoeia (6), noise (4), signal, animal (3), communication, crowd (2), material, action (1), conversation, individual, object (1),	traffic (32), sonic (18), nature (13), physical (4), voice, people (3),	technological (32), natural (13), human (7),
17	car signal (9), bip (8), birds, scan noise, tone, electronic beep, ATM (7), Electronic beep (5), car swoosh (4), traffic passing, car, distant (3), machine beeping, birds chirping, distant (2), car driving by, cars, click, drop-like, electronic beep, ATM , man voice, Traffic noises, voice, bids (1), bird, car passing, car, close, chatting, click, click, dops-like, click, drops, click, drops-like, clicks, clicks, drop-like, clicks, drops-like, cough, deep thump, Distant traffic, Electronic beeps, Electronic button beeps, footstep, gate opening, human voices, indistinct non human sound and birds, knock, man, men talking, motor buzzing, motorbike, distant, noise, people chatting, pitched tone (around B), rain pattering, rain pattering , rattle, scan noise (beep), something fallen, swish, ticking, traffic, traffic and birds, voices, water drop, wind gust, wooden noise (1),	car signal (9), electronic beep atm, bip (8), birds, scan noise, tone, electronic beep (5), car swoosh (4), traffic passing, car distant (3), machine beeping, birds chirping distant (2), car driving by, cars, click drop like, man voice, rain pattering, traffic noises, voice, bids (1), bird, car close, car passing, chatting, click, click dops like, click drops, click drops like, clicks, clicks drop like, clicks drops like, cough, deep thump, distant traffic, electronic beeps, electronic button beeps, footstep, gate opening, human voices, indistinct non human sound and birds, knock, man, men talking, motor buzzing, motorbike distant, noise, people chatting, pitched tone around b, rattle, scan noise beep, something fallen, swish, ticking, traffic, traffic and birds, voices, water drop, wind gust, wooden noise (1),	signal (37), vehicle (24), noise (18), bird (13), onomatopoeia, communication (5), action (3), conversation, geophony, body (2), crowd (1), individual, material, object (1),	traffic (61), sonic (31), nature (16), voice (10), physical (5), people (2),	technological (61), natural (16), human (12),
18	seagulls (13), male voice (4), car swoosh (3), door closing, keys, running, bash (2), human voices, keys rattling, male voices, men talking, men voices, People talking, Traffic noises, adult footsteps (1), bashes, bird, bunch of keys, car drving off?, cart, cart dominating, cart receding then stops, cart wheels, church bells, churchbells, distant, distant car, door close, door closing , door locked, door opening , door opening and closing, door slam, door slams and distant alarms, engine increasing and decreasing (chopper motorcycle), Engine noises, engine starting, footstep, footsteps, footsteps running, footsteps, kid running, human conversations, human conversations , human voice, human voice (close), human voices (distant), key rattling, keys jingling, low hum , male voice, loud, man, man voice, man's voice, men, motorbike, motrobike or skateboard wheels, softer, people talking, rhythmic indistinct noise non human, rhythmic noise maybe motorbike, rolling, rolling cart, rolling cart distant (behind corner?), rolling cart, approaching, noisier, same motorcycle far away (?), sea gulls, Seagull calls, seagulls far, steps, strong motorbike engine or skatebaord wheels?, thump, traffic, traffic hum, traffic noise, trolley, trolley lagguage, trolley weels, very far traffic noise, Waves hitting the boat, wheels rolling (1),	seagulls (13), door closing (4), male voice, car swoosh (3), keys, people talking, running, bash (2), human conversations, human voices, keys rattling, male voices, men talking, men voices, traffic noises, adult footsteps (1), bashes, bird, bunch of keys, car drving off, cart, cart dominating, cart receding then stops, cart wheels, church bells, churchbells distant, distant car, door close, door locked, door opening, door opening and closing, door slam, door slams and distant alarms, engine increasing and decreasing chopper motorcycle, engine noises, engine starting, footstep, footsteps, footsteps kid running, footsteps running, human voice, human voice close, human voices distant, key rattling, keys jingling, low hum, male voice loud, man, man voice, mans voice, men, motorbike, motrobike or skateboard wheels softer, rhythmic indistinct noise non human, rhythmic noise maybe motorbike, rolling, rolling cart, rolling cart approaching noisier, rolling cart distant behind corner, same motorcycle far away, sea gulls, seagull calls, seagulls far, steps, strong motorbike engine or skatebaord wheels, thump, traffic, traffic hum, traffic noise, trolley, trolley lagguage, trolley weels, very far traffic noise, waves hitting the boat, wheels rolling (1),	bird (17), vehicle (16), object (14), wheels (12), communication (10), body (9), conversation (7), crowd (6), action (5), onomatopoeia (4), signal (3), noise (2), geophony (1), group, individual, music (1),	traffic (31), voice (26), physical (19), nature (18), people (8), sonic (7),	human (34), technological (31), natural (18),
19	child (3), glass bottles, object on a surface, car (2), child voice, hit (plate), key clicking, metal clang, metal object, metal scratching, traffic passing, baby voices very far (1), Background road , blind , car approaching, further away, car engine, cars (in a tunnel), ceramic cups, child voice followed by adult, children, Children Southing, clacking, clacking , clinking, clinking, sunblind closing?, delivery truck, dishes, door, lock, doorlock again, engine noise, Engine Truck close, faint voice, faint voices, Far away man voice, Far away truck close , Far away voices, Far away woman voice , far kids and some anthropic sounds, footsteps, garbage bag, gate, Hammers, human voices, distant, kid whining , kids shouting, lifting roll door, light metal object, light metal objectchild, light object, machine hum, male voice, man, metal clink, metal door opening, motorbike, movement, indoor, newspaper, object, object mouved, opening of metallic doors, people chatting, plastic bag (?), plate clinking, plates clingin, putting stuff, setting table, sound of a van that starts from afar and gets closer and closer, traffic noise, close car engines , truck engine close, passing by, truck passing by, truck, engine, trunk , undefined, van passing through or leaf blower, vehicles passing, very faint voices, very quiet soundscape, white noise (1),	child (3), glass bottles, object on a surface, car (2), child voice, clacking, hit plate, key clicking, metal clang, metal object, metal scratching, traffic passing, baby voices very far (1), background road, blind, car approaching further away, car engine, cars in a tunnel, ceramic cups, child voice followed by adult, children, children southing, clinking, clinking sunblind closing, delivery truck, dishes, door lock, doorlock again, engine noise, engine truck close, faint voice, faint voices, far away man voice, far away truck close, far away voices, far away woman voice, far kids and some anthropic sounds, footsteps, garbage bag, gate, hammers, human voices distant, kid whining, kids shouting, lifting roll door, light metal object, light metal objectchild, light object, machine hum, male voice, man, metal clink, metal door opening, motorbike, movement indoor, newspaper, object, object mouved, opening of metallic doors, people chatting, plastic bag, plate clinking, plates clingin, putting stuff, setting table, sound of a van that starts from afar and gets closer and closer, traffic noise close car engines, truck engine, truck engine close passing by, truck passing by, trunk, undefined, van passing through or leaf blower, vehicles passing, very faint voices, very quiet soundscape, white noise (1),	object (28), vehicle (19), material (10), crowd (9), communication (6), individual, action (5), noise (3), onomatopoeia, body (1), conversation, machine (1),	physical (43), traffic (20), people (15), voice (8), sonic (6),	human (23), technological (20),

Day	Label_raw	Level_0	Level_1	Level_2	Level_3
20	bird (13), bird trill (12), birds, bird chirping (11), Nightingale (10), seagull (7), woman talking (6), bird chirp (4), car, human conversation (3), car passing by (2), dog barking, hit, laugh, man talking, man voice, rattle, single bird chirping, voices faint, woman, baby crying (1), background engine sound, Background voices, backgrpund engines and some steps, bash, bird chirping , bird chirping, slightly differently, bird chirps, bird song, breathe, car background, car honk, car passing, chatting, Chirping birds, Click, Conversation. Women, crashing metal, very distant, creaing, dishes, distant voices, distant voices, door close , door closing, Door knocking and woman shouting, female voice, female voices far, female voices, distant, gate, hard, distant bashes, heavy wheel downstairs, human voices, female, male, distant, indistinct human noise, indistinct human voice, keys rattle, kids. Far, Laughs, laughter, laughting, male laughing, male sinister laughter, male voice, man, man laugh, man laughing, Man talking nearby, metal chain, metalic door slam, metallic noise, metallic sound (chain?), metals/glass fall down, music, Nightingale , object, Opening door, peolple talking and birds, people talking, person laughing, pladtic bag, scratching, seagul, Seagull, soft bash, swish, thin melodic bird, traffic and people, Traffic sound, Vehicle passing by, very far car, very quite background - neighbors voice very far, water, water noise (?), wheel high pitch, whistle, woman shouting, Woman talking, woman voice, women talking (1),	bird (13), bird chirping (12), bird trill, birds, nightingale (11), seagull (8), woman talking (7), bird chirp (4), car, human conversation (3), car passing by (2), distant voices, dog barking, hit, laugh, man talking, man voice, rattle, single bird chirping, voices faint, woman, baby crying (1), background engine sound, background voices, backgrpund engines and some steps, bash, bird chirping slightly differently, bird chirps, bird song, breathe, car background, car honk, car passing, chatting, chirping birds, click, conversation women, crashing metal very distant, creaing, dishes, door close, door closing, door knocking and woman shouting, female voice, female voices distant, female voices far, gate, hard distant bashes, heavy wheel downstairs, human voices female male distant, indistinct human noise, indistinct human voice, keys rattle, kids far, laughs, laughter, laughting, male laughing, male sinister laughter, male voice, man, man laugh, man laughing, man talking nearby, metal chain, metalic door slam, metallic noise, metallic sound chain, metals glass fall down, music, object, opening door, peolple talking and birds, people talking, person laughing, pladtic bag, scratching, seagul, soft bash, swish, thin melodic bird, traffic and people, traffic sound, vehicle passing by, very far car, very quite background neighbors voice very far, water, water noise, wheel high pitch, whistle, woman shouting, woman voice, women talking (1),	bird (81), communication (20), conversation (18), vehicle (14), crowd (10), object (9), action (6), onomatopoeia, material (5), individual (3), animal (2), noise, wheels, music (1), signal (1),	nature (83), voice (38), physical (20), traffic (17), people (13), sonic (9),	natural (83), human (51), technological (17),
21	birds (13), bird (6), birds chirping (5), door, footsteps (4), voices, chatting (3), door slam, man, bump (2), bump, distant, dishes, Door closing, female voice, Knock, music, noise, steps, Steps, voice, voices , wind gut, ambulance (1), background traffic noise, bird chirping, bird-trill, Birds chirping, buckle clicking, bumps, car, car horn, car passing, car, distant, cart, child's voice, distant, chirp, church bell , classic music, click, click , clinking, crash, door opening, door slam , doors, drag chain, engine, far conversations, footstep, hit (glass), hit (object falling), hits (glass falling), hits and bumps, hitting object, honk, human voice, female, distant, human voices, closer, human voices, distant, keys, male voice, male voice , male voices, metal clang, metal clink , metal scratching, moving something, Murmur , noise on microphone, noise, distant, nose blowing(?), object dropping, object on a surface, objects, opening door, Opening door, scratch, swish, traffic, Traffic noise, traffic passing, tuned sound, distant, wings flapping, Woman talking (1),	birds (13), bird (6), birds chirping, voices, door (5), door slam (4), footsteps, steps, chatting (3), man, bump (2), bump distant, click, dishes, door closing, female voice, knock, male voice, music, noise, opening door, voice, wind gut, ambulance (1), background traffic noise, bird chirping, bird trill, buckle clicking, bumps, car, car distant, car horn, car passing, cart, childs voice distant, chirp, church bell, classic music, clinking, crash, door opening, doors, drag chain, engine, far conversations, footstep, hit glass, hit object falling, hits and bumps, hits glass falling, hitting object, honk, human voice female distant, human voices closer, human voices distant, keys, male voices, metal clang, metal clink, metal scratching, moving something, murmur, noise distant, noise on microphone, nose blowing, object dropping, object on a surface, objects, scratch, swish, traffic, traffic noise, traffic passing, tuned sound distant, wings flapping, woman talking (1),	bird (29), object (21), onomatopoeia (14), communication (13), body (10), vehicle (8), action (7), conversation (5), crowd, noise (4), signal, individual (3), material, music, geophony (2), rest (1), wheels (1),	nature (31), physical, voice (28), sonic (21), traffic (13), people (8), modifiers (1),	human (36), natural (31), technological (13),
22	birds (9), door closing (5), cart (3), airplane (2), baby, cocorita, keys, loud thump, seagull, shaking, the owner calls the dog with a whistle, bird song (whistle) (1), bird song descending, birds chirping, bumps, cat, child, claps very far, distant human voices, dog st, dog's footsteps, door clising, door slam, faint footsteps , footsteps, footsteps (female), footsteps (heels), gate, heavy door, heavy door or object, hit, hum people talking, human + dog steps, human steps, human voice, male, human voices and household sounds, insect-like rattling, keys rattling, knicking, knocking, low machine hum, man, man speaking, man voice, music far away, music that comes from far away , people talking, rattle, rattling, rolling cart, scraping, seagulls, steps, the owner calls the dog, ticking faint, voices, whistle, whistle calls, whistling, woman (1),	birds (9), door closing (5), cart (3), airplane (2), baby, cocorita, keys, loud thump, seagull, shaking, the owner calls the dog with a whistle, bird song descending (1), bird song whistle, birds chirping, bumps, cat, child, claps very far, distant human voices, dog st, dogs footsteps, door clising, door slam, faint footsteps, footsteps, footsteps female, footsteps heels, gate, heavy door, heavy door or object, hit, hum people talking, human dog steps, human steps, human voice male, human voices and household sounds, insect like rattling, keys rattling, knicking, knocking, low machine hum, man, man speaking, man voice, music far away, music that comes from far away, people talking, rattle, rattling, rolling cart, scraping, seagulls, steps, the owner calls the dog, ticking faint, voices, whistle, whistle calls, whistling, woman (1),	bird (17), object (12), action (8), body, communication, individual (5), onomatopoeia, animal (4), wheels, conversation (3), crowd (2), music, vehicle, machine (1), noise (1),	nature (21), physical (20), voice (19), sonic (8), people (7), traffic (7),	human (26), natural (21), technological (7),

Day	Label_raw	Level_0	Level_1	Level_2	Level_3
23	car (6), door (5), bird (3), birds, rain, thud, traffic passing, Brakes (2), bump, car braking, car, medium distance, click, locker?, drop, close, knock, People talking, Rain, screech, voices, wind gust, door opening and closing (1), (blank), adult passing by talking loudly, background conversations, background traffic noise, bash, bell, Bird chirping, bird song, birds chirping, caar door locked, car alarm, Car braking, car door open, car door slammed, car door, closing, car driving by, car passing, car passing by, chatting, clink (spoon in a cup?), clink, distant, Conversation very near between man and woman, cup hit, Different woman taking, distant hit metal, distant hit metal again, distant metal hit, Door closes, door closing, Door closing, door opening, door opening, and closing, door shutting, door slam, dripping running water, female 'byebye', female voice chatting at phone, female voice in backgound, heavy stuff unloaded, high pitched dripping, high pitched shrill noise, high-pitched shrill noise, hit, hit against railing, hit on iron pole, hit plastic bucket, hit sound, horn, human voice, close, moving L/R, human voice, distant, human voice, distant, moving, human voice, female, close, human voices, distant, indistinct voices, iron, loud thud, man, man and woman talking, metal, metal cling, metal hit, metal sound, Metallic clang, Metallic ringing, motorbike, close, nearby conversation, noise, distant, object on a surface, objects, rain and birds sound, raindrops, rattle, metal, rattling, Someone talking, Strong traffic noise, teardrop, thud , thuds, traffic in background, Traffic noise, Trafflic noises, truck passing by, voice, voice close, voice female, woman, woman passing by talking on the phone, woman talking, Woman talking, women chatting, women talking (1),	car (6), door (5), rain, thud (4), bird (3), birds, car braking, traffic passing, brakes (2), bump, car medium distance, click locker, door closing, door opening and closing, drop close, high pitched shrill noise, knock, people talking, screech, voices, wind gust, woman talking, adult passing by talking loudly (1), background conversations, background traffic noise, bash, bell, bird chirping, bird song, birds chirping, blank, caar door locked, car alarm, car door closing, car door open, car door slammed, car driving by, car passing, car passing by, chatting, clink distant, clink spoon in a cup, conversation very near between man and woman, cup hit, different woman taking, distant hit metal, distant hit metal again, distant metal hit, door closes, door opening, door shutting, door slam, dripping running water, female byebye, female voice chatting at phone, female voice in backgound, heavy stuff unloaded, high pitched dripping, hit, hit against railing, hit on iron pole, hit plastic bucket, hit sound, horn, human voice close moving l r, human voice distant, human voice distant moving, human voice female close, human voices distant, indistinct voices, iron, loud thud, man, man and woman talking, metal, metal cling, metal hit, metal sound, metallic clang, metallic ringing, motorbike close, nearby conversation, noise distant, object on a surface, objects, rain and birds sound, raindrops, rattle metal, rattling, someone talking, strong traffic noise, teardrop, thuds, traffic in background, traffic noise, trafflic noises, truck passing by, voice, voice close, voice female, woman, woman passing by talking on the phone, women chatting, women talking (1),	vehicle (26), object (19), onomatopoeia, action (15), conversation, bird (10), geophony (8), material (7), crowd (6), communication (5), noise (4), individual (3), signal, acoustic (1), rest (1),	physical (41), traffic (29), sonic (23), voice (20), nature (18), people (9), modifiers (2),	human (29), technological, natural (18),
24	harmonica (12), dog barking (5), birds (4), music, siren, bird (3), car, dog, male voice, man talking, thump, ambulance (2), bus, cough, human voices, distant, keys rattling, musical instrument, seagulls, traffic passing, voices, agitated voice (1), alarm (ambulance?), Background conversation, background indistinct conversasions, bids and people talking, Bird , bird chirps, bird song, bus whistling breaks(?), car engine closer, chatting, child, Chirping Bird , chuckle, clear throat, clearing throat, click, conversation , coughing, distant cars, distant hum, dog barking, brathign heavily, dog breathing, dog roaming, door, faint voices, far siren, far traffic sound, female voice, harmonica playing, hawking, hit, human voice, male, close, human voice, male, closer, increasing engine, indistinct human and non human vioces, keys, Laughs, laughter, loud barking small dog, man, melodica continues, melodica continues (to tonic), melodica playing, metal clips, metal noise - saw, metal sound (spoon in a cup?), Music , musical instrument (harmonica?), object, ok, Open bottle, people, police alarm, police siren, rattling, road, screeching (tires? seagull??), shaking, someone coughing, sparse bird chirps, Things moving, thud, thuds, traffic, Voice , whistle, whistling, Woman laughs, woman speaking, woman talking (1),	harmonica (12), dog barking (5), music, bird (4), birds, siren, car (3), dog, male voice, man talking, thump, ambulance (2), bus, cough, human voices distant, keys rattling, musical instrument, seagulls, traffic passing, voices, agitated voice (1), alarm ambulance, background conversation, background indistinct conversasions, bids and people talking, bird chirps, bird song, bus whistling breaks, car engine closer, chatting, child, chirping bird, chuckle, clear throat, clearing throat, click, conversation, coughing, distant cars, distant hum, dog barking brathign heavily, dog breathing, dog roaming, door, faint voices, far siren, far traffic sound, female voice, harmonica playing, hawking, hit, human voice male close, human voice male closer, increasing engine, indistinct human and non human vioces, keys, laughs, laughter, loud barking small dog, man, melodica continues, melodica continues to tonic, melodica playing, metal clips, metal noise saw, metal sound spoon in a cup, musical instrument harmonica, object, ok, open bottle, people, police alarm, police siren, rattling, road, screeching tires seagull, shaking, someone coughing, sparse bird chirps, things moving, thud, thuds, traffic, voice, whistle, whistling, woman laughs, woman speaking, woman talking (1),	music (24), bird (15), communication (14), vehicle, animal (12), conversation, signal (11), action (6), body, crowd, onomatopoeia, noise (3), object, individual (2), group (1), material (1),	sonic (33), voice (32), nature (27), traffic (25), physical (10), people (9),	human (41), natural (27), technological (25),
25	dog (19), birds (9), dog barking (7), male voice (4), seagull, click (3), dog barks, footsteps (2), humans speaking, Knock, man talking, man voice, object, sbam, seagulls, voice, male, close, 1 dog bark (1), Barking dog, bird, bird chirping, birds chirping, bump (door?), bumps, chatting, child, child voice, Chirping birds, Distant murmur, dog barking up, dogs barking, door, door closing, door shutting, door slam, door thud, Far away traffic noise, heavy door, man, Man and child talking, men talking, Men talking nearby, object on a surface, Opening door, people talking, sea gull, Seagull, shaking, some brid chirping, some city noise (traffic backgournd, low volume, some far voice in background, steps, thud , voice, male, close (indoor?) (1),	dog (19), birds (9), dog barking (7), seagull (5), male voice (4), click (3), dog barks, footsteps (2), humans speaking, knock, man talking, man voice, object, sbam, seagulls, voice male close, 1 dog bark (1), barking dog, bird, bird chirping, birds chirping, bump door, bumps, chatting, child, child voice, chirping birds, distant murmur, dog barking up, dogs barking, door, door closing, door shutting, door slam, door thud, far away traffic noise, heavy door, man, man and child talking, men talking, men talking nearby, object on a surface, opening door, people talking, sea gull, shaking, some brid chirping, some city noise traffic backgournd low volume, some far voice in background, steps, thud, voice male close indoor (1),	animal (33), bird (22), object (10), conversation (9), onomatopoeia, communication (7), crowd (5), body (3), action (2), individual, vehicle (2),	nature (55), voice (19), physical (12), sonic (9), people (7), traffic (2),	natural (55), human (26), technological (2),
26	birds (24), bird (11), child (4), bees (3), child talking, dishes, whir, child voice (2), chirp, whistle, animal (1), around eight beats, regular on a pitched drum, bird warning, bird warning call, bird whistling, car swoosh faint, chatting, children talking, classic music, cling, cling, plates, cutlery, distant birds, distant human voices, distant music, distant voices, door, door close, fly, glass, indistinct humam voice, kid's voice, closer, male voice, metallic sound or bell, mic handling noise, music, people talking faint, rubbing, scrapping, single voice, songbird, melodic, three chirps, traffic passing, very far sound of guitar, voices in background, whistling, woman talking, woman voice (1),	birds (24), bird (11), child (4), bees (3), child talking, dishes, whir, child voice (2), chirp, whistle, animal (1), around eight beats regular on a pitched drum, bird warning, bird warning call, bird whistling, car swoosh faint, chatting, children talking, classic music, cling, cling plates, cutlery, distant birds, distant human voices, distant music, distant voices, door, door close, fly, glass, indistinct humam voice, kids voice closer, male voice, metallic sound or bell, mic handling noise, music, people talking faint, rubbing, scrapping, single voice, songbird melodic, three chirps, traffic passing, very far sound of guitar, voices in background, whistling, woman talking, woman voice (1),	bird (43), communication (8), conversation (7), object (6), animal (5), crowd, individual (4), music, onomatopoeia, action (3), vehicle (2), acoustic (1), material, rest, signal (1),	nature (48), voice (15), physical (10), people (9), sonic (8), traffic (3), modifiers (2),	natural (48), human (24), technological (3),

Day	Label_raw	Level_0	Level_1	Level_2	Level_3
27	birds (5), dog barking (4), motorbike (3), birds (sparse) (2), dog, footsteps, man and woman talking, man singing, rattling, keys?, steps, traffic, battery car (1), bird, bird song, birds chirping , bump on mike, car, car driving by, car moving away, chatting, children, coin, different women voices, distant church bells (?), distant vehicle, dog barking loud , door bang, door closing, door shutting, drag container, female soft voice, female talking (distant), female voices, fluttering, footstep, gate, human voice, singing, close, human voices, close, human voices, distant, increasing engine, keys, keys jinging, machine, male singing, male voice passing by, man and woman speaking, man talking, metal, motor bike passing by, motor bike speeding up, motorbike passing, motorbike quite loud, other female talking (c loser, softer), people greeting each other, people talking, people talking (faint) , plastic/paper noise, roll door, roller shutter, scraping noise, maybe sliding door to van being opened, scratch noise, soft male voice, someone is singing, someone talking in a low voice, sunblind, woman speaking, woman talking (1),	birds (5), dog barking (4), motorbike (3), birds sparse (2), dog, footsteps, man and woman talking, man singing, rattling keys, steps, traffic, battery car (1), bird, bird song, birds chirping, bump on mike, car, car driving by, car moving away, chatting, children, coin, different women voices, distant church bells, distant vehicle, dog barking loud, door bang, door closing, door shutting, drag container, female soft voice, female talking distant, female voices, fluttering, footstep, gate, human voice singing close, human voices close, human voices distant, increasing engine, keys, keys jinging, machine, male singing, male voice passing by, man and woman speaking, man talking, metal, motor bike passing by, motor bike speeding up, motorbike passing, motorbike quite loud, other female talking c loser softer, people greeting each other, people talking, people talking faint, plastic paper noise, roll door, roller shutter, scraping noise maybe sliding door to van being opened, scratch noise, soft male voice, someone is singing, someone talking in a low voice, sunblind, woman speaking, woman talking (1),	conversation (13), bird (10), object, vehicle (9), action (7), animal, communication (6), body (5), crowd, material (3), wheels (2), group (1), individual, machine, noise, onomatopoeia, rest, signal (1),	voice (24), physical (20), nature (17), traffic (13), people (7), sonic (2), modifiers (1),	human (31), natural (17), technological (13),
28	child (5), male voice (4), voices, bird (3), birds, birds chirping, child voice, human voices, distant, bus (2), children, kid shouting, music, people talking, adult voices (1), airplane or big truck (faraway), animal, baby cry, background light noise and voices from neighbors , bids chirping, bird + exotic, bird fluttering, bird tweeting, closer, bird's wings flapping, birds (intense), birds and childrens talking with adults, chatting, child voice , child's voice, classic music, croak/fart, female, female voice, female voice in background, human voice, kid, closer, human voice, male, closer, larger bird, man, man sings, melodic, pigeon flying away, plane, plane fly pass, sound by TV , sound from a car, squeeking door, traffic pass, wings flapping, wooden noise (1),	child (5), child voice (4), male voice, voices, bird (3), birds, birds chirping, human voices distant, bus (2), children, kid shouting, music, people talking, adult voices (1), airplane or big truck faraway, animal, baby cry, background light noise and voices from neighbors, bids chirping, bird exotic, bird fluttering, bird tweeting closer, birds and childrens talking with adults, birds intense, birds wings flapping, chatting, childs voice, classic music, croak fart, female, female voice, female voice in background, human voice kid closer, human voice male closer, larger bird, man, man sings, melodic, pigeon flying away, plane, plane fly pass, sound by tv, sound from a car, squeeking door, traffic pass, wings flapping, wooden noise (1),	bird (18), communication, individual (10), crowd (7), vehicle, music (5), conversation (4), animal (1), material, object, spatial (1),	voice (22), nature (19), people (17), traffic (7), sonic (5), physical (2), modifiers (1),	human (39), natural (19), technological (7),
29	birds (5), footsteps (4), glass bottles (3), car in background (2), car, distant, female voice, key chain, keys rattling, male voice, steps, thump, traffic passing, woman, woman speaking, baby (1), bird song, birds chirping, birds chirping, distant, birds in background very far, birds, steps and woman talking, breaking glass, bump and hit, car, car door shut, car engine, car passing by, car very far, car, medium distance, car, medium distant, cars passing, cars, distant, door opening, indoor, door thud, engine motor low pitch, female steps, female voice in background, female voice more distant, foosteps, footsteps, female, footstes, glass bottles , glass thrown in bin, glass thrown into the differentiated bins for the glass, glass/ceramic, high pitch sound(far), human steps , human voice, female, close, human voice, male, close, moving , human voice, male, distancing, human voices, distant, loud male voice, loud metal bashing or glass crashing, low rumble, male voice in the foreground, man, man passing by talking, man speaking , man talking, manipulation on microphone, metal or glass crashing (as before), metallic noise, mixing chat, move a wooden chair, people talking, rattle, rattling, rattling, glass falling, rattling, glass falling and breaking, sea waves, seagull, sit on a wooden chair, steps people passing by, traffic, two men speaking, van engine low-pitch, voices, walk down the iron stairs, wings flapping, woman shoes walking, woman talking, woman talking/making a phone call, women talking and same man continues talking (1),	birds (5), footsteps (4), glass bottles, car distant (2), car in background, female voice, key chain, keys rattling, male voice, steps, thump, traffic passing, woman, woman speaking, baby (1), bird song, birds chirping, birds chirping distant, birds in background very far, birds steps and woman talking, breaking glass, bump and hit, car, car door shut, car engine, car medium distance, car medium distant, car passing by, car very far, cars distant, cars passing, door opening indoor, door thud, engine motor low pitch, female steps, female voice in background, female voice more distant, foosteps, footsteps female, footstes, glass ceramic, glass thrown in bin, glass thrown into the differentiated bins for the glass, high pitch soundfar, human steps, human voice female close, human voice male close moving, human voice male distancing, human voices distant, loud male voice, loud metal bashing or glass crashing, low rumble, male voice in the foreground, man, man passing by talking, man speaking, man talking, manipulation on microphone, metal or glass crashing as before, metallic noise, mixing chat, move a wooden chair, people talking, rattle, rattling, rattling glass falling, rattling glass falling and breaking, sea waves, seagull, sit on a wooden chair, steps people passing by, traffic, two men speaking, van engine low pitch, voices, walk down the iron stairs, wings flapping, woman shoes walking, woman talking, woman talking making a phone call, women talking and same man continues talking (1),	vehicle (17), body (13), conversation (12), action (10), bird, material (9), crowd (7), object (6), communication (5), individual (4), onomatopoeia, noise (2), geophony (1), machine, rest (1),	voice (30), physical (25), traffic (18), nature (11), people, sonic (6), modifiers (1),	human (41), technological (18), natural (11),
30	birds (5), rattling (4), car (3), cutlery plates, door, door closing, footsteps, birds chirping (2), birds, distant, children, distant traffic, hit, human activity sounds, keys rattling, kid, male voice, noise, scratch, thuds, woman, background distant human conversations (1), background soft contunous noise, beeps (distant, beeps, distant, bird, bird , bird fluttering, birds warning call, bump, car signal, car, distance, cat miaow, chain, child voice, click, cough, dishes and actvity, distant car/traffic, door bash loud, door locking, door shutting, door slam, door slams and noise, door thump, female voice, footstep, hit (door closing?), hit (plates?), hollow humming (vent?), human activity sounds/dishes, human activity/dishes, human voices, distant, jingling keys, keys, kids, louder bird chirp, machine buzzing, machine hum, man talking and other voices, metal crashing , metal scrape, metal scratching, metallic sound, mid-pitched humming, music, people talking, plates, rattle, rattling, glass, shuffling, sound of dishes, human activity and door slams, talking, traffic, traffic passing, van, low-freq engine, very loud door slam, voice, female, woman talking (1),	birds (5), rattling (4), car (3), cutlery plates, door, door closing, footsteps, beeps distant (2), bird, birds chirping, birds distant, children, distant traffic, hit, human activity sounds, keys rattling, kid, male voice, noise, scratch, thuds, woman, background distant human conversations (1), background soft contunous noise, bird fluttering, birds warning call, bump, car distance, car signal, cat miaow, chain, child voice, click, cough, dishes and actvity, distant car traffic, door bash loud, door locking, door shutting, door slam, door slams and noise, door thump, female voice, footstep, hit door closing, hit plates, hollow humming vent, human activity dishes, human activity sounds dishes, human voices distant, jingling keys, keys, kids, louder bird chirp, machine buzzing, machine hum, man talking and other voices, metal crashing, metal scrape, metal scratching, metallic sound, mid pitched humming, music, people talking, plates, rattle, rattling glass, shuffling, sound of dishes human activity and door slams, talking, traffic, traffic passing, van low freq engine, very loud door slam, voice female, woman talking (1),	action (19), object (16), bird (14), vehicle (11), individual (7), noise, onomatopoeia (6), body (5), communication, conversation, crowd (3), material, signal, machine (2), animal (1), music (1),	physical (38), traffic (16), nature (15), voice, sonic (14), people (10),	human (25), technological (16), natural (15),

Day	Label_raw	Level_0	Level_1	Level_2	Level_3
31	child (7), birds (5), dog (4), dog barking, door closing, kid's voice, cough (3), door, wind gust, bird (2), bump, child voice, dog bark, door closed, door slam, electronic beep, female voice, object drop, whistle, motorbike far away (1), background indstinct conversations, background traffic/city sound, beep/alarm, bike in water puddle?, birds chirping, birds chirping, closer, birds chirping, distant, brids chirping, car horn, chatting, clicking, cough , coughing, dishes, dog's barking, door shutting, gate opening, gate opening and closing, glass, human voice, female, close, kids, male voice, man, man clears throat, man coughing, man voice, man whistles, metal clink, mn whistleing, motorcycle, noise, rubbing (plastic?), scan noise, scan noise , steps, traffic noise in background, birds and chatting people very softly, traffic, distant, voice, voices, voices background, whisteling, whistling, woman, woman voice (1),	child (7), birds (5), cough (4), dog, dog barking, door closing, kids voice, door (3), wind gust, bird (2), bump, child voice, dog bark, door closed, door slam, electronic beep, female voice, object drop, scan noise, whistle, background indstinct conversations (1), background traffic city sound, beep alarm, bike in water puddle, birds chirping, birds chirping closer, birds chirping distant, brids chirping, car horn, chatting, clicking, coughing, dishes, dogs barking, door shutting, gate opening, gate opening and closing, glass, human voice female close, kids, male voice, man, man clears throat, man coughing, man voice, man whistles, metal clink, mn whistleing, motorbike far away, motorcycle, noise, rubbing plastic, steps, traffic distant, traffic noise in background birds and chatting people very softly, voice, voices, voices background, whisteling, whistling, woman, woman voice (1),	communication (19), object (14), animal (11), bird, individual (10), body (8), vehicle (5), action (4), signal, geophony (3), noise, onomatopoeia, conversation (2), crowd, material, wheels (1),	voice (29), nature (25), physical (20), people (12), traffic (10), sonic (6),	human (41), natural (25), technological (10),
34	dog barking (8), door closing, bird (4), birds, bump, dog, Barking Dog (3), baby (2), children voices, dog bark, dog bark , door, kid, Knock, traffic hum, van engine, voice, 3 thuds (1), baby crying, Background bustle, background traffic noise, Bird, bird , bird chirping, bird communication, bird song, birds animated conversation, birds chirping , birds chirping, close, birds chirping, distant, car, car , car in background, child, child voice, children , children voice, Children voice, Children voice , click sound, clinking, delivery truck engine, dishes, Distant bird , distant traffic, door slam, door slamped, door thud , engine (car?), faint voices, Gate closing , high pitch noise, hit, hit, indoor, household object , human voice, kids, distant, human voices, dis+tant, indistinct human voices and non human noises, indistinct woman voice, instrument note?, irregular high pitched rattling, keys, kid's voice (faint), light noisy background with sounds from birds and from houses of people and traffic very far , loading goods, machine click, making table, many birds, metal clink, mouse clicked/pressed/released, music, music, distant, noise, shaking, non human noise, object bashing, objects bashing, rattling, schreech, shaking, slam, someone getting out of the door, squeaking (scissor?), thud, thud (door?), Traffic, traffic in background, traffic passing, truck, Truck engine, voice , voices, wind gust, Woman Voice, Woman Voice (1),	dog barking (8), door closing, bird (6), birds (4), bump, dog, dog bark, barking dog (3), children voice, voice, baby (2), car, children voices, door, kid, knock, traffic hum, van engine, woman voice, 3 thuds (1), baby crying, background bustle, background traffic noise, bird chirping, bird communication, bird song, birds animated conversation, birds chirping, birds chirping close, birds chirping distant, car in background, child, child voice, children, click sound, clinking, delivery truck engine, dishes, distant bird, distant traffic, door slam, door slamped, door thud, engine car, faint voices, gate closing, high pitch noise, hit, hit indoor, household object, human voice kids distant, human voices dis tant, indistinct human voices and non human noises, indistinct woman voice, instrument note, irregular high pitched rattling, keys, kids voice faint, light noisy background with sounds from birds and from houses of people and traffic very far, loading goods, machine click, making table, many birds, metal clink, mouse clicked pressed released, music, music distant, noise shaking, non human noise, object bashing, objects bashing, rattling, schreech, shaking, slam, someone getting out of the door, squeaking scissor, thud, thud door, traffic, traffic in background, traffic passing, truck, truck engine, voices, wind gust (1),	object (22), animal (19), bird, vehicle (18), communication (13), onomatopoeia, crowd (7), action (6), individual, music (3), noise, geophony (1), machine, material (1),	nature (39), physical (29), sonic (19), traffic, people (13), voice (13),	natural (39), human (26), technological (19),
35	baby crying (6), baby (4), dog barking, wind gust, baby coo (3), baby giggling, dishes, female voice, baby babbling (2), bump, child voice, children, Crying child, Cutlery sounds, dog, dog bark, hits and bumps, kitchen noise, hits, kitchen noise, hits, plates, human voices, distant, acordion music (1), baby , Background music, Barking dog, bird chirp, bird fluttering, birds, birds chirping, Birds chirping, birds sing all the time, breaking branch, car driving-by, car passing, car, distant, chatting, child coughing, child singing, click, cups, plates, cutlery, cutlery plates, Dishes, distant motocycle, engine passing, Far away voices, female voice distant, female voices, flapping of wings, folk song, high pitched noise, hit, hitting hollow object, Knocs, male voices, motorbike, medium distance, music - accordion carrousel, music children choir, music played by an accordion, music, accordeon, folk, music, choir, object dropping, people talking, plates, radio music, scream (distant), scream, distant, thuds, thump, traffic passing, Vehicle passing by, very quite background - crockery sounds from from neighboring houses, Voices, voices singing, wings flapping, wings flutter, woman, woman voice, Women talking (1),	baby crying (6), baby (5), dishes (4), dog barking, wind gust, baby coo (3), baby giggling, female voice, baby babbling (2), birds chirping, bump, child voice, children, crying child, cutlery sounds, dog, dog bark, hits and bumps kitchen noise, hits kitchen noise, hits plates, human voices distant, scream distant, acordion music (1), background music, barking dog, bird chirp, bird fluttering, birds, birds sing all the time, breaking branch, car distant, car driving by, car passing, chatting, child coughing, child singing, click, cups plates, cutlery, cutlery plates, distant motocycle, engine passing, far away voices, female voice distant, female voices, flapping of wings, folk song, high pitched noise, hit, hitting hollow object, knocs, male voices, motorbike medium distance, music accordion carrousel, music accordeon folk, music children choir, music choir, music played by an accordion, object dropping, people talking, plates, radio music, thuds, thump, traffic passing, vehicle passing by, very quite background crockery sounds from from neighboring houses, voices, voices singing, wings flapping, wings flutter, woman, woman voice, women talking (1),	communication (25), individual (10), animal (9), bird, music, action (8), object, vehicle, crowd (6), noise, onomatopoeia, geophony (4), conversation (3), body (1),	voice (29), nature (22), sonic (21), people (16), physical, traffic (8),	human (45), natural (22), technological (8),

Day	Label_raw	Level_0	Level_1	Level_2	Level_3
37	birds (9), steps (5), cart (4), footsteps, people talking, bip (3), cart rolling, hit, trolley, car (2), car engine, car swoosh, keys, screech, Something being dragged, Traffic noises, voices, alarm, distant, six times (1), alarm/recurring jingle, background city noise, bang door, bash, bash thud, bird, birds chirping, Birds chirping, bus screech, car signals faint, cars, distant, cart rolling noisily, chair, chatting, distant beep, distant human voices, distant traffic, distant whitle, door , door closing, door opening, door slam very far, dragged object, engine, car?, engine, truck, Footsteps, gentle noise in background - iindistinct voices, hit plastic, hits and bumps, human voices, female and male, closer, indistinct conversations getting closer, indistinct human , indistinct human voice, loud van, motor starting, low-frequency motor revving, man + woman voices, man screams, metal grinding screech, metal object, metallic sound/keys?, mumbling, noise, People talking, revving car engine, scratching of something on the ground and , shopping stroller wheels rolling or alike, Something falls, Something scrapes, thump, traffic, traffic hum, traffic passing, van engine, vent hollow noise, voices, male quite loud, woman and man talking, woman man talking, woman talking with man (1),	birds (9), footsteps (5), people talking, steps, cart (4), bip (3), cart rolling, hit, trolley, birds chirping (2), car, car engine, car swoosh, keys, screech, something being dragged, traffic noises, voices, alarm distant six times (1), alarm recurring jingle, background city noise, bang door, bash, bash thud, bird, bus screech, car signals faint, cars distant, cart rolling noisily, chair, chatting, distant beep, distant human voices, distant traffic, distant whitle, door, door closing, door opening, door slam very far, dragged object, engine car, engine truck, gentle noise in background iindistinct voices, hit plastic, hits and bumps, human voices female and male closer, indistinct conversations getting closer, indistinct human, indistinct human voice, loud van motor starting, low frequency motor revving, man woman voices, man screams, metal grinding screech, metal object, metallic sound keys, mumbling, noise, revving car engine, scratching of something on the ground and, shopping stroller wheels rolling or alike, something falls, something scrapes, thump, traffic, traffic hum, traffic passing, van engine, vent hollow noise, voices male quite loud, woman and man talking, woman man talking, woman talking with man (1),	vehicle (22), bird (12), wheels, action (10), body, conversation, object, signal (7), onomatopoeia (6), crowd (5), communication (4), noise, material (1),	traffic (41), voice (24), physical (21), nature (12), sonic (10), people (5),	technological (41), human (29), natural (12),
38	birds (3), traffic, ambulance (2), car, close, cars engine noise, rattling, traffic passing, two men talking, wind on mike, People talking (1), bird chirping, birds (intense), bus, car, car engine starting, car passing, cars, close, cars, distant, Chirping Bir, Conversation , dog, engine, Equipment moving, Far away siren, female voices, human voices, male, incessant birdcalls, indistinct human voice, male voices, men talking, music, distant, scraping object, scraping objects, siren, distant, traffic hum, Train, Train arrival , Train departure, Truck engine, vehicles, woman talking, woman voice , women talking faint, women voices (1),	birds (3), traffic, ambulance (2), car close, cars engine noise, rattling, traffic passing, two men talking, wind on mike, bird chirping (1), birds intense, bus, car, car engine starting, car passing, cars close, cars distant, chirping bir, conversation, dog, engine, equipment moving, far away siren, female voices, human voices male, incessant birdcalls, indistinct human voice, male voices, men talking, music distant, people talking, scraping object, scraping objects, siren distant, traffic hum, train, train arrival, train departure, truck engine, vehicles, woman talking, woman voice, women talking faint, women voices (1),	vehicle (22), bird (7), conversation, action (5), communication (4), signal, crowd (2), geophony, animal (1), music (1),	traffic (26), voice (11), nature (10), physical (5), people (2), sonic (1),	technological (26), human (13), natural (10),
39	birds (6), bird, chirping, close (3), vehicle, bird-like rattling (2), birds chirping, blind opening, Children talking, human voices, distant, people talking faint, traffic, Traffic noise (1), Traffic noise far away, Voices, (blank), bird, bird like rattling, bird tweet, close, birds chirping faint, bump, door shutting, car, distant, cars, child, child voice, child's voice faint, children voice, Chirping birds, Clacking, crowd, delivery truck, dog, dog , door, door opening, Door opening, door opening and closing, door squeek, doorbell, Doorbell, footsteps, heavy object, high-pitched bird chirps, hit, human voices, medium distance, indistinct children voice, indistinct human voice, indistinct non human , insect-like fluttering, kick ball, large bird, low pitch noise, shuffling, low pitch noise, suffling, male laughter, male voices, man laughing, metal scratching, microphone manipulation, noise on microphone, object, old person voice, people talking, People talking nearby, seagull, shouting/seagulls, distant, shuffle, low pitch, Shutting door, small bird warning, steel like noise, Steps, traffic passing, van, engine, close, voices, whining/seagulls, distant, whistling, wind gust (1),	birds (6), bird chirping close (3), bird like rattling, vehicle, birds chirping (2), blind opening, children talking, dog, door opening, doorbell, human voices distant, people talking faint, traffic, voices, bird (1), bird tweet close, birds chirping faint, blank, bump door shutting, car distant, cars, child, child voice, children voice, childs voice faint, chirping birds, clacking, crowd, delivery truck, door, door opening and closing, door squeek, footsteps, heavy object, high pitched bird chirps, hit, human voices medium distance, indistinct children voice, indistinct human voice, indistinct non human, insect like fluttering, kick ball, large bird, low pitch noise shuffling, low pitch noise suffling, male laughter, male voices, man laughing, metal scratching, microphone manipulation, noise on microphone, object, old person voice, people talking, people talking nearby, seagull, shouting seagulls distant, shuffle low pitch, shutting door, small bird warning, steel like noise, steps, traffic noise, traffic noise far away, traffic passing, van engine close, whining seagulls distant, whistling, wind gust (1),	bird (22), vehicle (12), object (10), communication (9), crowd, conversation (6), action (4), animal (3), noise, rest, body (2), material, signal, geophony (1), individual, onomatopoeia (1),	nature (26), voice (17), physical (16), traffic (14), people (10), sonic (4), modifiers (3),	human (27), natural (26), technological (14),
41	birds (12), car (5), footsteps, voices (4), baby (3), door, cart (2), door closing, door slam, indistinct non human noise, Shutting door, steps, truck door slam, baby voice (1), birds in background, bump, car , car door, car in background, car passing rumbling , car very far, cart rolling, cart rolling away, Children playing, Chirping birds, construction machinery, Conversation between a man and a woman, cough, door bang, door closes, Door closes, electronic beep, Engine noises, female voice, female voice in background, female voices, Footsteps, Heavy traffic noise, Heavy vehicle passing nearby and stopping, huan voice, male, closer, human voice, male and female, closer, human voices chatting, indistinct human voice, indistinct human voices, Keys, loud cart rolling, loud truck passing by, low freq engine, male voice, male voices, man, man and woman passing by talking, man and woman talking, man complaining, Man talks, People talking, Seagulls, skate, Something swipes, Steps, Strong rattle, trolley, Trolley rattling, truck, truck arriving, truck door, voice, voices chatting - baby and birds, woman, woman and man speaking and walking (1),	birds (12), car (6), footsteps, voices (4), baby (3), door, steps, cart (2), door closes, door closing, door slam, indistinct non human noise, shutting door, truck door slam, baby voice (1), birds in background, bump, car door, car in background, car passing rumbling, car very far, cart rolling, cart rolling away, children playing, chirping birds, construction machinery, conversation between a man and a woman, cough, door bang, electronic beep, engine noises, female voice, female voice in background, female voices, heavy traffic noise, heavy vehicle passing nearby and stopping, huan voice male closer, human voice male and female closer, human voices chatting, indistinct human voice, indistinct human voices, keys, loud cart rolling, loud truck passing by, low freq engine, male voice, male voices, man, man and woman passing by talking, man and woman talking, man complaining, man talks, people talking, seagulls, skate, something swipes, strong rattle, trolley, trolley rattling, truck, truck arriving, truck door, voice, voices chatting baby and birds, woman, woman and man speaking and walking (1),	vehicle (16), bird (15), object, body (10), communication, conversation (9), wheels (8), action (5), crowd, individual, noise (2), machine (1), onomatopoeia, signal (1),	voice (29), traffic (26), physical (20), nature (15), people (10), sonic (3),	human (39), technological (26), natural (15),

Day	Label_raw	Level_0	Level_1	Level_2	Level_3
46	birds (20), child (8), dog (6), dog bark, dog barking, bird (5), child try to count (3), traffic passing, cart rolling (2), door closing, kid counting, man, woman, woman talking, 2nd woman talking (1), 3 dog barks, baby, baby voice, bashing objects dropping, bird , bird song, birds chirping throughout, boy talking, car, car door shut, car swoosh, chatting + children voices, child counting, child talking and playing, child voice, dog bark farther away, Dog barking , dog barks 3 times, door loud bash, door shutting, door slam, female and male voices, female voice, female voice close, footsteps, gate closing, happy mather voice, hit, distant, human voice, female, close, human voices, indistinct human voices, kid counting aloud, kid talking, kid's voice playing, kid's voice, playful, kids' voices, playing, machine whistle, man ululating, object on a surface, other birds, people talking, rattling, scratch, like wheels in gravel, scratching noises, something passing by, something slammed on the ground, squeel, thud, thump, vehicle sound, very quiet background - mather and child voices all the time, voice, woohoo (1),	birds (20), child (8), dog barking (7), bird (6), dog, dog bark, child try to count (3), traffic passing, cart rolling (2), door closing, kid counting, man, woman, woman talking, 2nd woman talking (1), 3 dog barks, baby, baby voice, bashing objects dropping, bird song, birds chirping throughout, boy talking, car, car door shut, car swoosh, chatting children voices, child counting, child talking and playing, child voice, dog bark farther away, dog barks 3 times, door loud bash, door shutting, door slam, female and male voices, female voice, female voice close, footsteps, gate closing, happy mather voice, hit distant, human voice female close, human voices, indistinct human voices, kid counting aloud, kid talking, kids voice playful, kids voice playing, kids voices playing, machine whistle, man ululating, object on a surface, other birds, people talking, rattling, scratch like wheels in gravel, scratching noises, something passing by, something slammed on the ground, squeel, thud, thump, vehicle sound, very quiet background mather and child voices all the time, voice, woohoo (1),	bird (29), animal (22), communication (16), individual (13), conversation (9), object, crowd (6), vehicle, action (4), onomatopoeia, wheels (3), body (1), machine (1),	nature (51), voice (26), people (19), physical (13), traffic (10), sonic (4),	natural (51), human (45), technological (10),
47	dog (7), ball (5), cart (4), cart rolling (3), dog barking, Metallic rattling, baby (2), ball bouncing, ball kicked, birds, child voice, loud male voice, male voice, playing footbal, adult playing with child (1), around 10 loud ball bounces, baby voice, baby's voice, bag, manipulated, ball , Ball bounces, Ball bounces, ball bouncing, kids kicking it around, bird chirps (masked by noises), breath, car, car passing, child, Child talking, children, children talking, crowd , distant hum, distant voices, Dog barking, dog communication, door, female singing, female voice, Footsteps, hit, skateboard, human voices chatting, human voices, kids and adult, male, female, mid distance, human voices, male, close, kid talking to parent, kid's voice, kid's voice, close, kids, kids talking, loud rattling toy cart, male talking, male voices, man, man and woman conversation, metal scratching, parents talking, people talking, People talking, plastic package, playing football, rattling, footsteps of kids, scooter, skateboard hit on the ground, skating, stomp, stomping, toys etc, trolley, voices and kids (1),	dog (7), ball (6), cart (4), dog barking, cart rolling (3), metallic rattling, baby (2), ball bounces, ball bouncing, ball kicked, birds, child voice, loud male voice, male voice, people talking, playing footbal, adult playing with child (1), around 10 loud ball bounces, baby voice, babys voice, bag manipulated, ball bouncing kids kicking it around, bird chirps masked by noises, breath, car, car passing, child, child talking, children, children talking, crowd, distant hum, distant voices, dog communication, door, female singing, female voice, footsteps, hit skateboard, human voices chatting, human voices kids and adult male female mid distance, human voices male close, kid talking to parent, kids, kids talking, kids voice, kids voice close, loud rattling toy cart, male talking, male voices, man, man and woman conversation, metal scratching, parents talking, plastic package, playing football, rattling footsteps of kids, scooter, skateboard hit on the ground, skating, stomp, stomping, toys etc, trolley, voices and kids (1),	action (14), wheels (13), animal (12), communication, conversation (10), object (8), crowd (7), individual, body (5), bird (3), material (2), vehicle, noise (1),	voice (27), physical (24), nature (15), traffic, people (14), sonic (1),	human (41), natural (15), technological (15),
48	birds (8), older woman, bird (6), female, bird chirping (5), footsteps, child (4), talking, Children shouting (3), children voices, noise, plastic bag, child's voice (2), male voice, man voice, people talking, rollerskaters, very loud female 1, voices, woman, women conversation, (blank) (1), background: steps, birds, and noise of traffic very far, bang, bash, bird chirps, Birdcall, birds chirping faint, boom, ballon exploding, boy voice, car swoosh, child exclaims Adieu, child shout, child shrieks, child voice said ok, child whining, children, children loud shouting 'adios', Children talking, chirp, chorus Adieu, family voice chatting very quite, hits, toys?, human voices, conversation, kids and adults, human voices, kids, human voices, women, conversation, very close, key ratlling, key rattling, keys, kid shouting, kids shouting 'adios', laughter, loud kids conversation, loud thud, loud woman, male voice in reply, male voice in response, man, many voices, mixed chat among female 1 female 2 and child voices, mixing chat female voices say goodbye, older woman Adieu, Ongoing conversation, rubbing, shot, shot, loud bang, soft sneeze, steps, very loud female 2, very loud voice female 2, very lound voice faemale 1, woman laughing loudly, woman voice, women speaking loudly, women voices, young child, younger child, younger child exclaims Adieu (1),	birds (8), older woman, bird (6), female, bird chirping (5), footsteps, child (4), talking, children shouting (3), children voices, noise plastic bag, childs voice (2), male voice, man voice, people talking, rollerskaters, very loud female 1, voices, woman, women conversation, background steps birds and noise of traffic very far (1), bang, bash, bird chirps, birdcall, birds chirping faint, blank, boom ballon exploding, boy voice, car swoosh, child exclaims adieu, child shout, child shrieks, child voice said ok, child whining, children, children loud shouting adios, children talking, chirp, chorus adieu, family voice chatting very quite, hits toys, human voices conversation kids and adults, human voices kids, human voices women conversation very close, key ratlling, key rattling, keys, kid shouting, kids shouting adios, laughter, loud kids conversation, loud thud, loud woman, male voice in reply, male voice in response, man, many voices, mixed chat among female 1 female 2 and child voices, mixing chat female voices say goodbye, older woman adieu, ongoing conversation, rubbing, shot, shot loud bang, soft sneeze, steps, very loud female 2, very loud voice female 2, very lound voice faemale 1, woman laughing loudly, woman voice, women speaking loudly, women voices, young child, younger child, younger child exclaims adieu (1),	communication (31), individual (24), bird (23), conversation (20), body (7), crowd (5), onomatopoeia (4), action (3), material, object, vehicle (2), wheels, music (1), noise, rest (1),	voice (58), people (29), nature (23), physical (9), sonic (6), traffic (4), modifiers (1),	human (87), natural (23), technological (4),

Day	Label_raw	Level_0	Level_1	Level_2	Level_3
49	child (10), birds (6), man, footsteps (5), bump (4), bash (3), bumps, loud clack, male voice, car (2), cart, children, clack, hit (skateboard), hits, human voices, kids, keys rattling, loud thud, man voice, metal object noise, skateboard, straps being cut, trolley, 4 knocks (1), adult female scoling, baby screem, bicycle-cart rolling, bird, bird chirp, bird chirping, boards hit the wall, bunch of keys, Buzz, cart rolling, cart rolling passing by, cart rolling, scraping, chid and woman speaking, child's shriek, children , children speaking, children's voices, childrens talking with mother, click, door bell, door closing, door slam faraway, dragging, electronic beep, father and son , going upstairs, heavy door closing, heavy object falling noise, heavy objet falling noise, hits (skateboard), hits (woond), hits and bumps (skateboard), human voice, male, close, human voices, close, human voices, kid, male, female, close, indistinct human voices, indistinct metal noise, key, keys clicking, keys jingling, kid, kid shouting, kid talking, kid voice, kid's voice, kids, kids and parents, Knock, loud burst, loud shot, low machine, mam speak with her children, man speaking, man talking, ball rebounding, metal object falling, motor bike passing by, motorbike, motrobike whosh, near and far hit , noisy, people talking, playing with skate, pluck, Rattle , Rattle from soft to loud, roller skates or scooters, slamming and rolling, running, scraping, senior woman scolding, sharp hits, skate, skate passing by, skateboarding, skates and other wheels, probably, very close, steel pole falling down, swish, thuds, traffic passing, undetermined urban noise, Voices of children and adults in the background, walking with slipper, woman speaking (1),	child (10), birds (6), man, footsteps (5), bump (4), bash (3), bumps, children, loud clack, male voice, car (2), cart, clack, hit skateboard, hits, human voices kids, keys rattling, loud thud, man voice, metal object noise, skateboard, straps being cut, trolley, 4 knocks (1), adult female scoling, baby screem, bicycle cart rolling, bird, bird chirp, bird chirping, boards hit the wall, bunch of keys, buzz, cart rolling, cart rolling passing by, cart rolling scraping, chid and woman speaking, children speaking, childrens talking with mother, childrens voices, childs shriek, click, door bell, door closing, door slam faraway, dragging, electronic beep, father and son, going upstairs, heavy door closing, heavy object falling noise, heavy objet falling noise, hits and bumps skateboard, hits skateboard, hits woond, human voice male close, human voices close, human voices kid male female close, indistinct human voices, indistinct metal noise, key, keys clicking, keys jingling, kid, kid shouting, kid talking, kid voice, kids, kids and parents, kids voice, knock, loud burst, loud shot, low machine, mam speak with her children, man speaking, man talking ball rebounding, metal object falling, motor bike passing by, motorbike, motrobike whosh, near and far hit, noisy, people talking, playing with skate, pluck, rattle, rattle from soft to loud, roller skates or scooters slamming and rolling, running, scraping, senior woman scolding, sharp hits, skate, skate passing by, skateboarding, skates and other wheels probably very close, steel pole falling down, swish, thuds, traffic passing, undetermined urban noise, voices of children and adults in the background, walking with slipper, woman speaking (1),	individual (25), onomatopoeia (24), wheels (22), action (18), communication (11), bird (9), conversation, object (8), body (7), crowd, noise (5), vehicle (4), signal (2), acoustic (1), machine, material (1),	people (32), sonic (29), traffic, physical (27), voice, nature (9), modifiers (1),	human (59), technological (29), natural (9),
51	hammer blows (6), hammering, metal knocking (5), door closing (4), hitting, voices, birds (3), cough, hit, metal object falling, high pitch, metal clang, (blank) (2), big metal item falling, bird, bump, car, door, door slam, footsteps, hammer hitting, hit, loud thud, people talking, shuffling, sound of iron/man at work, thud, baby (1), Background conversetion , birds chirping, distant, bumps, car distant, car engine, car starting, chatting, child voice, clapping, claps of a child, clinking, creaking, crowd din, door bash, engine increasing and decreasing (car on a ramp), exotic animal, female voice, footsole screech, footstep, Hammer, Hammer , hammer hitting metal, hit, mid pitch , hitting again, hitting metal/stone repeatedly, human voices, crowd, distance, indistinct metal noise, keys or metal object, keys/metal obj, kid scream (?) distant, knock, Knock, loud car, close, loud door bash, male voice, male voices, man, man talking, Man Voice , metal crash, metal scratch, motorbike, noise, object, open door , people chatting very busy, people talking in the street, plastic hitting, rattle, rattling, high pitch, rattling, metal objects, rubbing (rubber sole), screech, shout, high pitch , sound of iron / man at work, steps, Tools, traffic, traffic passing, Truck accelerates, undistinct human voices, voices , workers- metallic noise (1),	hammer blows (6), hammering, metal knocking (5), voices, door closing (4), hitting, birds (3), cough, hit metal object falling high pitch, metal clang, big metal item falling (2), bird, blank, bump, car, door, door slam, footsteps, hammer, hammer hitting, hit, knock, loud thud, people talking, shuffling, sound of iron man at work, thud, baby (1), background conversetion, birds chirping distant, bumps, car distant, car engine, car starting, chatting, child voice, clapping, claps of a child, clinking, creaking, crowd din, door bash, engine increasing and decreasing car on a ramp, exotic animal, female voice, footsole screech, footstep, hammer hitting metal, hit mid pitch, hitting again, hitting metal stone repeatedly, human voices crowd distance, indistinct metal noise, keys metal obj, keys or metal object, kid scream distant, loud car close, loud door bash, male voice, male voices, man, man talking, man voice, metal crash, metal scratch, motorbike, noise, object, open door, people chatting very busy, people talking in the street, plastic hitting, rattle, rattling high pitch, rattling metal objects, rubbing rubber sole, screech, shout high pitch, sound of iron man at work, steps, tools, traffic, traffic passing, truck accelerates, undistinct human voices, workers metallic noise (1),	object (30), action (22), onomatopoeia (12), body (11), material, communication (10), vehicle, conversation (7), bird (6), crowd (5), machine (3), noise, individual (2), rest, animal (1),	physical (63), voice (28), sonic (15), traffic (13), nature (7), people, modifiers (2),	human (35), technological (13), natural (7),

Day	Label_raw	Level_0	Level_1	Level_2	Level_3
52	bird (7), bird call (5), Chirping bird (4), church bells, people talking, birds (3), thud, bird chirping (2), car door shut, car engine, cart, hit, male voice, scratch, 12 bells (1), acoustic signal, alarm (van reverse?), background buzz, bash boum (faint), bell tower, bells and trolley passing by, bids, bird communication, bird song, bird's tweet, birds and people chatting, birds, distant, bump, car door open, car door opening, car going away, car leaving, chatting, chirp birds, Chirping Birds , Church bells, church bells (major second), clicks, Construction noises, cough, distant voices, door, door closing, Door closing, door open, Engine noises, engine starting, engine switches on, rear gear beep, enginee starter and increasing, Equipment noises, exotic animal, foot stamp, glass decoration noise, hammer, hammer blows, hammer blows (construction working), Hammer hitting, Hammering, heavy door (van), hitting like a carpenter, hitting wood, human voice, male, close, human voice, male, closer, human voices, distant, hummer blows, insect, jingle/whistle, kid scream (faint), machine hum, man , man at phone, Man talking, metal , metal hit, metal object, metallic noise, Movement of objects, objects, objects scratch, thuds, opening and closing of a small door, People bustle, People talking, people talking faint, rattle, rattling, rattling sound, rubbing, scratch, drop, signal, Someone talking, sound of hammer, sounds of work in progress very far, start car engine, start engine, stick hit, three beeps, trolley down slope, truck door closes, Truck enging starts, Truck starting engine and going, van door, van drives away, vehicle motor, voice, din, voices, faint, Woman talking, wood? hits, work in progress (1),	bird (7), bird call (5), church bells, people talking, chirping bird (4), birds (3), thud, bird chirping (2), car door shut, car engine, cart, door closing, hit, male voice, scratch, 12 bells (1), acoustic signal, alarm van reverse, background buzz, bash boum faint, bell tower, bells and trolley passing by, bids, bird communication, bird song, birds and people chatting, birds distant, birds tweet, bump, car door open, car door opening, car going away, car leaving, chatting, chirp birds, chirping birds, church bells major second, clicks, construction noises, cough, distant voices, door, door open, engine noises, engine starting, engine switches on rear gear beep, enginee starter and increasing, equipment noises, exotic animal, foot stamp, glass decoration noise, hammer, hammer blows, hammer blows construction working, hammer hitting, hammering, heavy door van, hitting like a carpenter, hitting wood, human voice male close, human voice male closer, human voices distant, hummer blows, insect, jingle whistle, kid scream faint, machine hum, man, man at phone, man talking, metal, metal hit, metal object, metallic noise, movement of objects, objects, objects scratch thuds, opening and closing of a small door, people bustle, people talking faint, rattle, rattling, rattling sound, rubbing, scratch drop, signal, someone talking, sound of hammer, sounds of work in progress very far, start car engine, start engine, stick hit, three beeps, trolley down slope, truck door closes, truck enging starts, truck starting engine and going, van door, van drives away, vehicle motor, voice din, voices faint, woman talking, wood hits, work in progress (1),	bird (28), object (17), vehicle (15), signal (14), action (12), conversation (11), onomatopoeia (10), crowd (9), noise (4), communication (3), machine, material, wheels, animal (2), body, individual (1), music (1),	traffic (35), physical (32), nature (30), voice (16), sonic (15), people (10),	technological (35), natural (30), human (26),
54	church bells (5), dog barking, bird chirping (3), bump, soft, car horn, female voice, people talking, bell (2), bird, bird communication, Car honking, car horns, car whistling, claxon, cough, distant voices, hit, door, horn, object hit, voices, 2 loud thuds (1), angry dog, backpack zipper sound, bash, bell and woman talking, bird chirp, bird chirps, bird tweeting, bird warning chirps, bird warning class, birds, Birds chattering, birds chirping, birds warning chirps, car, car approaching, then accelerating away, Car engine, car horn sustained, car leaving, car rhythmic h onk, car rhythmic honks, chatting, child, church bell twice major second, clacson, clacson loud, clincking keys , Crowd murmurs, dog, dog , dog bark loudly, Dog barks, door closing, door shut, door slam, Electronic bell plays, engine revving, engine switching on, entrance door, female voice close, front door opens, lock sound, gate, hit, indoor, human voice chatting, human voice, female, close, human voices, human voices, close, human voices, distant, indistinct background conversations, keys, keys rattling, kid, loud car horn, loud honking car, loud thud, luggage case with wheel, motorbike, object, people passing by talking, People talking, pjoff, rattling, rattling noise, remote unlock car, scraping, Something drops, Something slams, steps, thuds, traffic passing, uncork a bottle, undefined, unlocking, van rising or air blower, van, close, moving, vehicle, voices loud, woman talking, women talking with children (1),	church bells (5), dog barking, people talking (4), bird chirping (3), bump soft, car horn, female voice, bell (2), bird, bird communication, car honking, car horns, car whistling, claxon, cough, distant voices, dog, hit door, horn, object hit, voices, 2 loud thuds (1), angry dog, backpack zipper sound, bash, bell and woman talking, bird chirp, bird chirps, bird tweeting, bird warning chirps, bird warning class, birds, birds chattering, birds chirping, birds warning chirps, car, car approaching then accelerating away, car engine, car horn sustained, car leaving, car rhythmic h onk, car rhythmic honks, chatting, child, church bell twice major second, clacson, clacson loud, clincking keys, crowd murmurs, dog bark loudly, dog barks, door closing, door shut, door slam, electronic bell plays, engine revving, engine switching on, entrance door, female voice close, front door opens lock sound, gate, hit indoor, human voice chatting, human voice female close, human voices, human voices close, human voices distant, indistinct background conversations, keys, keys rattling, kid, loud car horn, loud honking car, loud thud, luggage case with wheel, motorbike, object, people passing by talking, pjoff, rattling, rattling noise, remote unlock car, scraping, something drops, something slams, steps, thuds, traffic passing, uncork a bottle, undefined, unlocking, van close moving, van rising or air blower, vehicle, voices loud, woman talking, women talking with children (1),	signal (26), bird (16), vehicle (13), action (12), animal (10), conversation, crowd (9), object, onomatopoeia (8), communication (5), body (3), noise, individual (2), group (1), material, music, wheels (1),	traffic (40), nature (26), physical (22), voice (18), people (12), sonic (12),	technological (40), human (30), natural (26),
56	church bells (4), cough (3), thud, bell (2), bicycle, bicycle rolling, bird, child voice, children, Children shouting, children voices, children's laughters, crowd, distant crowd, footsole screech, hit, kids shouting, people talking, thuds, alarm (1), bike, bird chirps, birds chirping, bottle crashing, car lock beep, chatting + children voices, child bike, child laughing, child laughter playing, child scream, child screaming, child's shriek, children (faint), children chasing, children footsteps running, children laughing while playing, children laughters, children playing, children running, children shriek, church bell, church bells , coughin, door, Door closes, door closing, door slam, Electronic hum, Footsteps (running), gear, glass, glass bottles, Glasses clinking, high pitch child laughter, hit hollow object, hits, human voices (closer), indistinct voices / busy people chatting, laugh, machine, man and woman conversation, man coughs, man's voice (closer), rattling (bottle?), rubber sole, rubber sole in the ground (?), running footsteps (kids), skater, skater/trolley, someone is running, three bashes, trolley weels, undefined (bicycle?), undefined like before (bike?), voices crowd, whistle, women talking (1),	church bells (5), cough (3), thud, bell (2), bicycle, bicycle rolling, bird, child voice, children, children shouting, children voices, childrens laughters, crowd, distant crowd, footsole screech, hit, kids shouting, people talking, thuds, alarm (1), bike, bird chirps, birds chirping, bottle crashing, car lock beep, chatting children voices, child bike, child laughing, child laughter playing, child scream, child screaming, children chasing, children faint, children footsteps running, children laughing while playing, children laughters, children playing, children running, children shriek, childs shriek, church bell, coughin, door, door closes, door closing, door slam, electronic hum, footsteps running, gear, glass, glass bottles, glasses clinking, high pitch child laughter, hit hollow object, hits, human voices closer, indistinct voices busy people chatting, laugh, machine, man and woman conversation, man coughs, mans voice closer, rattling bottle, rubber sole, rubber sole in the ground, running footsteps kids, skater, skater trolley, someone is running, three bashes, trolley weels, undefined bicycle, undefined like before bike, voices crowd, whistle, women talking (1),	communication (21), body (14), signal (11), wheels, crowd (8), action (6), onomatopoeia, conversation (5), bird (4), material, object, individual (3), vehicle (2), machine (1),	voice (40), traffic (25), physical (14), people (11), sonic (6), nature (4),	human (51), technological (25), natural (4),

Day	Label_raw	Level_0	Level_1	Level_2	Level_3
60	bird (8), child (6), Bird chirping (5), children, Children shouting, kids shouting, closer (4), kids' scooter (3), baby (2), birds, birds, closer, children voice, distant voices, human voices, squirr (bell?), Wheels rattling, woman, amplified female (1), baby crying, background voices, bell, bike, birds chirping, birds, distant, boum, broadcast, car, car honk , cart, cart rolling, cart rolling wheels, passing by, child shout, child's shriek, Children screaming, Children talking, children voices, door, female voice , footstep, footsteps, Footsteps, footsteps , footsteps , high pitched squirr (bell?), hit object, hit wood, hitting, kid squeel, kid's voice close, kids, kids shouting, kids voices (playing, shouting), kids' shouting, knocking, many birds, men, motorbike, music, nose blowing, people talking, people talking , recorded human voice, female (PA?), rolling , rolling over of something (dices?), rumble (motorcycle one?), speaker voice, steps, tapping noise, toy, toy or child bike, Traffic noises, voices, wheel, wheels , wooden object (1),	bird (8), child (6), bird chirping (5), children, children shouting, footsteps (4), kids shouting closer, kids scooter (3), baby (2), birds, birds closer, children voice, distant voices, human voices, kids shouting, people talking, squirr bell, wheels rattling, woman, amplified female (1), baby crying, background voices, bell, bike, birds chirping, birds distant, boum, broadcast, car, car honk, cart, cart rolling, cart rolling wheels passing by, child shout, children screaming, children talking, children voices, childs shriek, door, female voice, footstep, high pitched squirr bell, hit object, hit wood, hitting, kid squeel, kids, kids voice close, kids voices playing shouting, knocking, many birds, men, motorbike, music, nose blowing, recorded human voice female pa, rolling, rolling over of something dices, rumble motorcycle one, speaker voice, steps, tapping noise, toy, toy or child bike, traffic noises, voices, wheel, wheels, wooden object (1),	bird (20), communication (18), individual (17), wheels (12), crowd (11), body (7), action (5), signal, conversation (4), object, vehicle, onomatopoeia (2), group (1), music, noise (1),	people (29), voice, traffic (21), nature (20), physical (9), sonic (4),	human (58), technological (21), natural (20),
62	birds (6), footsteps (5), dog bark (3), bike (2), Birdcalls, car, child, children, dog barking, dog barks, door closing, hit wood, male voice, Someone laughing, steps, traffic passing, voices, animal (1), animal (dog?), audible conversation, audible conversations, background din, background street activity sound (families), bicycle wheel, bird, bird song, bird's tweet, birds chirping, bump, Children shouting, children voice, Crowd murmurs, distant bird chirps, dog, dog?, door, door creaking, female laughing, female voice, footstep, Footsteps, footsteps, soft, gate closing, high-heel, hit, hit (object falling?), human voices, crowd, distant, human voices, female, close, kid scream, kids, kids, laughing, playing, laugh, laughter again, laughter, female, loud door shutters , loud laugh, machine buzzing, metal blind closing, metallic noise, metallic shutter, noise/twirl, people, people talking faint, people talking in the distance, pulling down shop roll door, rattle, rolling door, rope skipping, scratch, sea gull, senior lady chatting, senior lady laughing, Shutters closing, Someone talking, Something knocks, squeek, squoink noise, timer, two loud bashes, sliding door, two women conversation, woman, woman laughs, woman laught close and talk while leaving , woman shoes walking, women talking while coming (1),	birds (6), footsteps, dog bark (3), bike (2), birdcalls, car, child, children, dog, dog barking, dog barks, door closing, hit wood, male voice, someone laughing, steps, traffic passing, voices, animal (1), animal dog, audible conversation, audible conversations, background din, background street activity sound families, bicycle wheel, bird, bird song, birds chirping, birds tweet, bump, children shouting, children voice, crowd murmurs, distant bird chirps, door, door creaking, female laughing, female voice, footstep, footsteps soft, gate closing, high heel, hit, hit object falling, human voices crowd distant, human voices female close, kid scream, kids, kids laughing playing, laugh, laughter again, laughter female, loud door shutters, loud laugh, machine buzzing, metal blind closing, metallic noise, metallic shutter, noise twirl, people, people talking faint, people talking in the distance, pulling down shop roll door, rattle, rolling door, rope skipping, scratch, sea gull, senior lady chatting, senior lady laughing, shutters closing, someone talking, something knocks, squeek, squoink noise, timer, two loud bashes sliding door, two women conversation, woman, woman laughs, woman laught close and talk while leaving, woman shoes walking, women talking while coming (1),	communication (17), bird (14), body (12), animal (11), action (10), conversation (9), object (8), individual (6), crowd (5), onomatopoeia, vehicle, wheels (3), group (1), machine, material, noise, signal (1),	voice (38), nature (25), physical (19), people (12), traffic (10), sonic (6),	human (50), natural (25), technological (10),
64	birds (10), dog barking (7), birds, distant (5), child (4), bird (3), child voice, exotic animal, car (2), dishes noise, dog, dog bark, door, footsteps, keys rattling, man, man voice, People talking, women talking, bird communication (1), Birdcalls, birds chirping, bunch of keys, car starting, car, distant, chid speaking, chidren talking with adult, child and adult, child and man speking, children, children and women talking, Children talking, din, Dishes clashing, door bashing, door close, door closing, door opening, door shutting, dropping metal objects, father and daughter, female voices, glass bottle, glass bottles, glass, hit, glass, rattling, human voices, closer, dialogue adult/kid, human voices, crowd, distant, human voices, dialogue, close, indistinct human voices, key, key chain, keys jingle, Keys jingling, kid shouting, kid talking, kid's voice, kids, machine, male voice, male voice (diffuse), male voice loud/close, man speaking, man talking to himself, men talking, mosquito, paper, paper crackling, people, people talking, plastic bag, plates, Shutter rattles, Someone humming, traffic hum, Traffic noises, traffic passing, truck, two female adults conversation, passing by, undefined, undistinguished chatting, voice, women speaking, Wrapper crinkles, young lady (1),	birds (10), dog barking (7), birds distant (5), child (4), bird (3), child voice, exotic animal, people talking, car (2), dishes noise, dog, dog bark, door, footsteps, keys rattling, man, man voice, women talking, bird communication (1), birdcalls, birds chirping, bunch of keys, car distant, car starting, chid speaking, chidren talking with adult, child and adult, child and man speking, children, children and women talking, children talking, din, dishes clashing, door bashing, door close, door closing, door opening, door shutting, dropping metal objects, father and daughter, female voices, glass bottle, glass bottles, glass hit, glass rattling, human voices closer dialogue adult kid, human voices crowd distant, human voices dialogue close, indistinct human voices, key, key chain, keys jingle, keys jingling, kid shouting, kid talking, kids, kids voice, machine, male voice, male voice diffuse, male voice loud close, man speaking, man talking to himself, men talking, mosquito, paper, paper crackling, people, plastic bag, plates, shutter rattles, someone humming, traffic hum, traffic noises, traffic passing, truck, two female adults conversation passing by, undefined, undistinguished chatting, voice, women speaking, wrapper crinkles, young lady (1),	bird (21), conversation (19), animal (15), object, communication (12), individual (11), vehicle (8), action (7), crowd (4), material, noise (3), body (2), group (1), machine (1),	nature (36), voice (33), physical (26), people (16), traffic (9), sonic (3),	human (49), natural (36), technological (9),

Day	Label_raw	Level_0	Level_1	Level_2	Level_3
65	baby (5), beep (3), birds, bumps, kids, bird chirping (2), bird, pigeon, bump, cart, child voice, footsteps, hit, male voice, muffled voices, object movement, Something drops, thud, a slam (1), airflow, airplane, airplane passing, alarm, Baby babbles, baby coo, baby voice , background city traffic noise, not too loud, background conversations, beep sound, beeps, bird, bird chirping, soft, birds chirping, birds, poeple chating, trolley passing by, can opening, car, car engine, car passing by, car woosh, cart bumping over an obstacle, cart on a step, cart on a step 2, cart rolling, chatting, child, child cry, child talking, children, Children talking, crowd, distant motorbike revving?, door, Electronic beeping, electronic keyboard sound, footstep, Footsteps, heavy thud, high pitch dragging noise, hit and bump, hits, hits and bumps, distant, hits hollow plastic, human voice, male, close, human voice, male, closer, infant, instrumental music, kid, loud male voice, machine, machine beeping, machine periodic , male humming, man , man speaking clearly, men talking, metal scratching, Metallic grinding, music, people, people speaking faint , People talking, pigeon, pigeons cooing , pingeon, childrens talking, plates, rattle, moving things, rattling, scrape metal, shuffle, singing, Someone mumbling, Something metallic being dragged, squeaking, starting of melody, steps, talking, thuds, thump, trolley, two persons talking, van driving by, vehicle motor and children, walking, whistle (1),	baby (5), beep (3), birds, bumps, footsteps, kids, bird chirping (2), bird pigeon, bump, cart, child voice, hit, male voice, muffled voices, object movement, something drops, thud, a slam (1), airflow, airplane, airplane passing, alarm, baby babbles, baby coo, baby voice, background city traffic noise not too loud, background conversations, beep sound, beeps, bird, bird chirping soft, birds chirping, birds poeple chating trolley passing by, can opening, car, car engine, car passing by, car woosh, cart bumping over an obstacle, cart on a step, cart on a step 2, cart rolling, chatting, child, child cry, child talking, children, children talking, crowd, distant motorbike revving, door, electronic beeping, electronic keyboard sound, footstep, heavy thud, high pitch dragging noise, hit and bump, hits, hits and bumps distant, hits hollow plastic, human voice male close, human voice male closer, infant, instrumental music, kid, loud male voice, machine, machine beeping, machine periodic, male humming, man, man speaking clearly, men talking, metal scratching, metallic grinding, music, people, people speaking faint, people talking, pigeon, pigeons cooing, pingeon childrens talking, plates, rattle moving things, rattling, scrape metal, shuffle, singing, someone mumbling, something metallic being dragged, squeaking, starting of melody, steps, talking, thuds, thump, trolley, two persons talking, van driving by, vehicle motor and children, walking, whistle (1),	individual (15), onomatopoeia (14), bird (12), action (11), conversation, communication (10), vehicle, signal (9), wheels (8), body (6), crowd, object (4), material (3), music, machine (2), noise, group (1),	traffic (29), voice (27), people (22), sonic (19), physical (18), nature (12),	human (49), technological (29), natural (12),
69	dog barking (7), child (6), bird (3), children, woman, alarm/whistle (2), children voices, door hinge, engine, human voice, kids, close, man, screeching, steps, animal (1), baby, background conversations parents kids, Barking dog, bike or toy, bird tweeting, swallow, bird tweeting, swallow , birds, birds chirping, birds chirping, distant, birds tweeting, birds, swallows, bump, car, car traffic sounds, chatting (overcrowded space), child , child scream, child speaking, child voice, child's cry, child's descending shriek, child's shriek, Children revelry, Clicking, clink, close voice, close voices, crowd din, dog, door closing, Door closing, Far barking dog, footsteps, Girl shout, Girl shouting, high pitch whistle, high-pitched machine, human voice, close, human voice, kid, close, human voice, kids, shouting, human voice, male, close, human voices, crowd, male female kids, human, crowd in a market , keys, kid shouting, kids, kids playing loud, kids running, shouting, stomping, Light traffic noise, Man voice, market, metallic door opening, motorike, noise, manipulating, people talking, rattling, rattling sounds, rattling, metal, rubbing (rubber sole), running, scooter, step+bike wheel, stpes, sustained shrill, ticking, trolley little wheels - metallic, Unidentified noise, voice, wheels rattling, wheels, stroller, skate…? (1),	child (7), dog barking, bird (3), children, woman, alarm whistle (2), bird tweeting swallow, children voices, door closing, door hinge, engine, human voice kids close, man, screeching, steps, animal (1), baby, background conversations parents kids, barking dog, bike or toy, birds, birds chirping, birds chirping distant, birds swallows, birds tweeting, bump, car, car traffic sounds, chatting overcrowded space, child scream, child speaking, child voice, children revelry, childs cry, childs descending shriek, childs shriek, clicking, clink, close voice, close voices, crowd din, dog, far barking dog, footsteps, girl shout, girl shouting, high pitch whistle, high pitched machine, human crowd in a market, human voice close, human voice kid close, human voice kids shouting, human voice male close, human voices crowd male female kids, keys, kid shouting, kids, kids playing loud, kids running shouting stomping, light traffic noise, man voice, market, metallic door opening, motorike, noise manipulating, people talking, rattling, rattling metal, rattling sounds, rubbing rubber sole, running, scooter, step bike wheel, stpes, sustained shrill, ticking, trolley little wheels metallic, unidentified noise, voice, wheels rattling, wheels stroller skate (1),	individual (18), communication (13), crowd (12), animal (11), bird (10), body (7), onomatopoeia, object (6), vehicle, wheels, conversation (4), action (3), noise, signal (2), machine (1),	people (30), voice (24), nature (21), traffic (15), sonic (10), physical (9),	human (54), natural (21), technological (15),
